# 4polar-STORM polarized super-resolution imaging of actin filament organization in cells

**DOI:** 10.1038/s41467-022-27966-w

**Published:** 2022-01-13

**Authors:** Caio Vaz Rimoli, Cesar Augusto Valades-Cruz, Valentina Curcio, Manos Mavrakis, Sophie Brasselet

**Affiliations:** 1 Aix Marseille Univ, CNRS, Centrale Marseille, Institut Fresnel, F-13013 Marseille, France; 2 Institut Curie, PSL Research University, UMR144 CNRS, Space-Time imaging of organelles and Endomembranes Dynamics Team, F-75005 Paris, France; 3 Inria Centre Rennes-Bretagne Atlantique, SERPICO Project Team, F-35042 Rennes, France

**Keywords:** Super-resolution microscopy, Biological fluorescence, Image processing

## Abstract

Single-molecule localization microscopy provides insights into the nanometer-scale spatial organization of proteins in cells, however it does not provide information on their conformation and orientation, which are key functional signatures. Detecting single molecules’ orientation in addition to their localization in cells is still a challenging task, in particular in dense cell samples. Here, we present a polarization-splitting scheme which combines Stochastic Optical Reconstruction Microscopy (STORM) with single molecule 2D orientation and wobbling measurements, without requiring a strong deformation of the imaged point spread function. This method called 4polar-STORM allows, thanks to a control of its detection numerical aperture, to determine both single molecules’ localization and orientation in 2D and to infer their 3D orientation. 4polar-STORM is compatible with relatively high densities of diffraction-limited spots in an image, and is thus ideally placed for the investigation of dense protein assemblies in cells. We demonstrate the potential of this method in dense actin filament organizations driving cell adhesion and motility.

## Abstract (web summary)

Single-molecule localisation microscopy does not give orientation information. Here the authors combine Stochastic Optical Reconstruction Microscopy (STORM) with single molecule orientation and wobbling measurements using four-polarisation image splitting, 4polar-STORM.

## Introduction

**Fig. 1:**
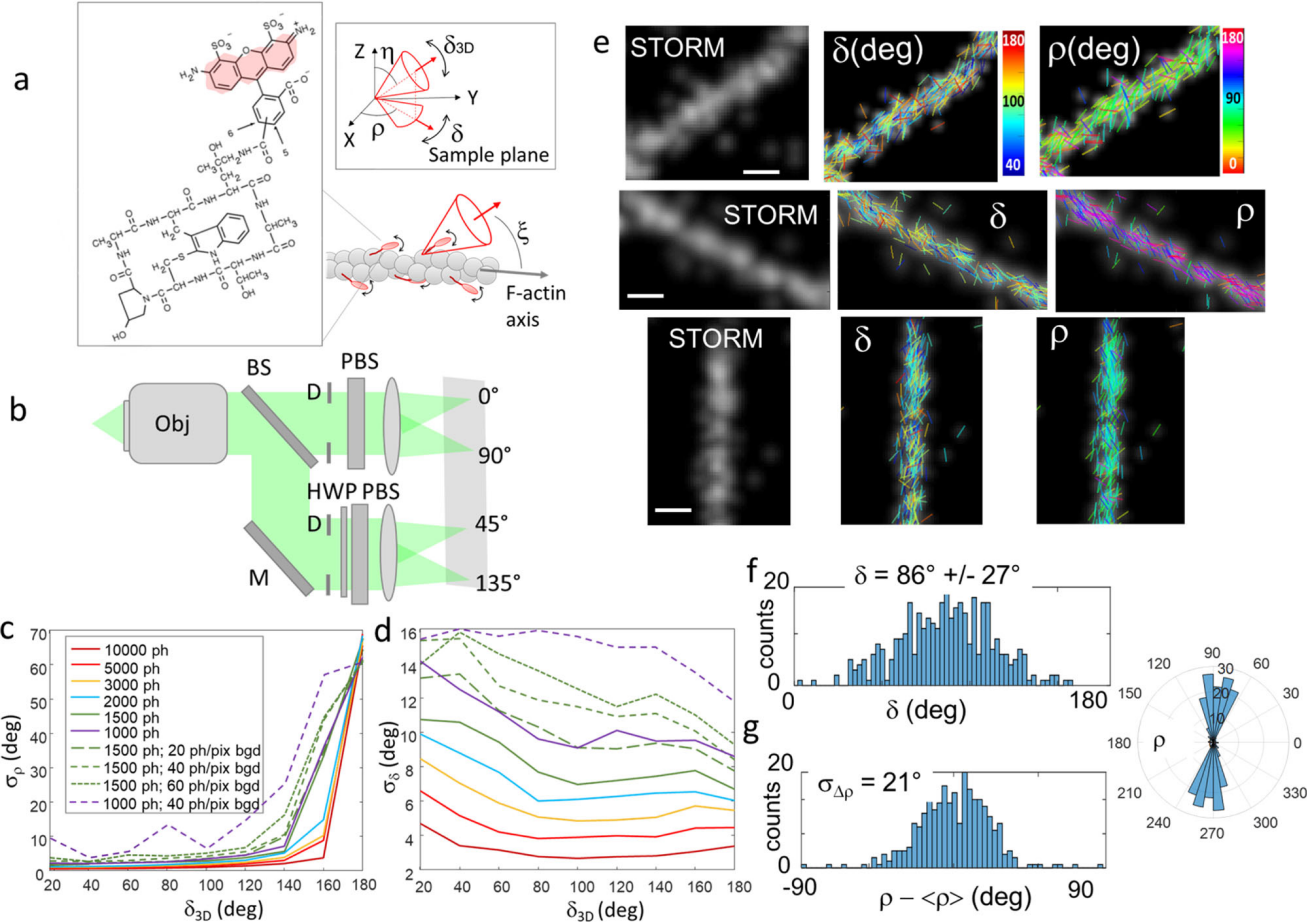
4polar-STORM allows single molecule orientational parameters retrieval in STORM imaging. **a** Schematic representation of a wobbling Alexa Fluor 488 (AF488)-phalloidin conjugate labeling an actin filament (F-actin) (the fluorophore moiety is highlighted in red). (ρ,η): mean orientation of a single fluorophore in 3D; δ_3D_: wobbling cone angle of the fluorophore in 3D; δ: projection in the sample plane; ξ: mean orientation of the fluorophore relative to the actin filament axis. **b** Schematic optical setup of 4polar-STORM imaging. BS, beam splitter; M, mirror; D, diaphragm; HWP, half-wave plate; PBS, polarizing beam splitter. **c** Monte Carlo simulations (see Methods) of the expected precision on ρ for various δ (η = 90°, ρ = 30°). Total intensities (ph, photons) and background levels (ph/pix, photons per pixel) are summed over all four polarized channels. **d** Corresponding simulations for the δ precision at different δ values (η = 90°) (the results do not depend on ρ). Same color code as in (**c**). **e** 4polar-STORM images of single AF488-phalloidin molecules labeling single actin filaments. Left panels depict single-molecule localization STORM images (blurred using a Gaussian filter width 0.3 pixel (=39 nm)). Middle and right panels depict single-molecule wobbling (δ) and orientation (ρ) measurements overlaid with the STORM image in grayscale. Each single molecule is represented as a stick whose orientation is ρ relative to the horizontal axis and whose color is the measured parameter (ρ or δ). Scale bars, 170 nm. Similar results were obtained on 5 different samples (about 5–10 filaments per sample). **f** Experimental δ histogram obtained on a straight region (see (**e**)) for thresholded intensity and localization precision (see Text). **g** Corresponding ρ histogram in both standard and polar-plot representations, relative to the average within the measured region of interest, Δρ = ρ − <ρ>. σ_Δρ_ is the standard deviation of Δρ. Source data are provided as a Source Data file.

Protein conformation and the precise way in which proteins arrange in space to form higher-order macromolecular assemblies are key elements of biological functions in cells and tissues. Adhesion of animal cells to the extracellular matrix as well as actin remodelling in space and time are determinant for cell mechanics driving essential biological processes, including immune responses and tissue development. Understanding the function of a protein and its interaction with its partners necessitates that we observe its organization at the nanometer scale, both in position and orientation. Current methods reporting protein organization such as electron microscopy or X-ray diffraction are however not yet applicable to a live imaging context. Single-molecule localization microscopy (SMLM) has brought considerable progress towards this goal, enabling imaging with a resolution down to tens of nanometers even in live cells^1–4^. However, while these methods report the localization of single molecules with high precision, they do not measure their orientation. If fluorophores are linked to the proteins of interest rigidly enough^5^, reporting their orientation could provide precious information on the structural organization of the attached proteins and on their conformational behaviors, which is inherently missing in localization-based optical imaging methods. While measuring fluorophore orientation precisely and accurately is a challenge that is of high interest in the field of SMLM imaging, it is however a delicate task. First, it is necessary to not only measure their 3D orientation, averaged over the imaging time, but also the extent of their orientation fluctuations, which naturally occurs when fluorophores wobble at fast time scales^5^ (Fig. 1a). A failure to uncouple their mean orientation from their fluctuations makes it impossible to determine accurately how the fluorophore-conjugated molecules are organized and can lead to misleading interpretations^5^. Second, orientation and spatial position are difficult to disentangle in SMLM, because of their intrinsic coupling in the process of the formation of their PSF image in a microscope^6,7^. The development of an optimal method to disentangle spatial position, mean orientation and orientation fluctuations is still an ongoing research^8^. One approach is to encode the orientation and wobbling information into the shape of the single molecules’ point spread function (PSF), using custom-designed phase or birefringent masks^9–13^. This strategy has led to recent successful demonstrations in in vitro samples^10–13^. PSF engineering however induces an increase in the PSF size of two to four times its initial size, and necessitates up to three times larger pixel sampling than in regular SMLM imaging. This limits its use in complex and densely-labeled structures such as in cells^14^ where PSFs are likely to overlap at spot densities above 1 molecule/μm^2^ (here spot-density defines the density of single-molecule diffraction-limited PSFs present at the same time in an image). PSF engineering also involves the implementation of complex phase/birefringent masks, complex data processing as well as stringent calibrations to avoid sources of bias such as optical aberrations. Along similar lines, exploiting PSF shape changes due to image defocusing^15,16^ or to the proximity of the molecule to the coverslip interface^17^ has been proposed, however with similar limitations as encountered in PSF engineering, and the need to know the distance of the molecule to the coverslip for the latter. Another approach is to use polarization projections of the image plane, and perform ratiometric intensity measurements between different polarization channels. Two-orthogonal polarization splitting has allowed fluorescence anisotropy measurements in isotropic environments^18^ and used to quantify actin filament alignment in 2D^5^, however with the inconvenience of an estimation ambiguity for fluorophore orientations symmetric relative to the polarization axes. Additionally, two-orthogonal polarization splitting does not efficiently decouple orientation from wobbling. Both ambiguities have been waived by the use of a four-polarization projection scheme^19–22^. A strong limitation still present in currently reported four-polarization split approaches is however that due to the high numerical apertures used, the intensities measured are strongly influenced by the off-plane 3D orientation of the fluorophores, resulting in large inaccuracies in the determination of their wobbling^23^. The failure to provide accurate measurements of fluorophore wobbling even for 2D organizations lying in the sample plane has precluded its use as an additional readout for protein organization. Importantly, single-molecule studies using four polarizations projections have not yet been applied to super-resolution imaging and have so far been limited to situations employing sparse labeling or/and photobleaching to obtain single fluorophores^19–22^. In this work, we make this approach applicable to quantitative 2D orientational super-resolution imaging in cells. We combine Stochastic Optical Reconstruction Microscopy (STORM) with single-molecule orientation and wobbling measurements using four-polarization image splitting in a method called 4polar-STORM. 4polar-STORM imaging is based on a relatively simple implementation of integrated intensity measurements. It keeps diffraction-limited PSFs close to Gaussian shapes, of sizes of a few pixels as in regular SMLM, and is thus ideally placed for the determination of the organization of dense protein assemblies in cells. To decrease bias sources and make 4polar-STORM imaging quantitative, we use a slightly reduced detection numerical aperture. This allows not only to determine the 2D mean orientation and wobbling of fluorophores in a reliable way for single molecules lying in the sample plane, but also to infer indirectly the 3D orientation of molecules that are tilted off plane.

With this approach, we reveal the nanometric-scale structural organization of actin filaments inside dense actin filament-based structures involved in the adhesion and motility of cells, notably stress fibers (SFs) and lamellipodia. Actin is at the center of interest in the development of super-resolution imaging methods where so far, only localization-based images have been exploited^24–29^. Here we use 4polar-STORM imaging to quantify the orientational behavior of fluorophores in single actin filaments used as a reference, and in fixed cells. We show that actin filaments are highly aligned in all types of SFs in cells, in line with EM studies^30,31^, but also include populations of actin filaments oriented off-plane. We evidence the perturbation of actin organization by mild pharmacological inhibition of myosin II activity and image actin organization in the dense meshwork of the lamellipodium at the leading edge of motile cells. Such organizations, generally studied by EM imaging^32–36^, have not yet been reported with super-resolution light microscopy methods to our knowledge, due to the high actin density in this area^24^.

## Results

### 4polar-STORM imaging applied to actin filaments

Fluorophores attached to a protein act as emission dipoles. While their position is directly defined as the center of their PSF image, their orientation is not directly extractable. A fluorophore is represented by its mean orientation (ρ,η) averaged over the imaging integration time, and its wobbling angle (δ_3D_) explored during this integration time (Fig. 1a). The goal of 4polar-STORM is to measure the fluorophore’s orientation and wobbling projected in the sample plane (ρ,δ) (Fig. 1a), based on the projection of the fluorescence signal on four polarizations channels along the directions 0°, 45°, 90°, and 135° respectively (0° corresponding here to the horizontal direction of the sample) (Fig. 1b), under total internal reflection with close to isotropic excitation in the sample plane. Theoretical developments of Supplementary Note 1 show that (ρ,δ) can be retrieved independently from two simple ratiometric factors, ${P}_{0}=\left({I}_{0}-{I}_{90}\right)/\left({I}_{0}+{I}_{90}\right)$ and ${P}_{45}=\left({I}_{45}-{I}_{135}\right)/\left({I}_{45}+{I}_{135}\right)$ (Supplementary Figs. S2, S3), with $\left({I}_{0},{I}_{45},{I}_{90},{I}_{135}\right)$ the single molecules’ intensities integrated over their PSFs along the four polarized channels. We note that δ is the sample-plane 2D projection of the wobbling cone angle δ_3D_, it therefore differs from the real 3D wobbling value δ_3D_. At large off-plane tilt angles in particular (small η angle in Fig. 1a), the projection of the wobbling cone angle is biased and δ is an overestimation of δ_3D_, as pointed out in earlier works^5,23^. Theoretical calculations accounting for the detection numerical aperture (NA) and the tilt angle of the fluorophore, show that a solution for minimizing this bias is to lower the detection NA of the microscope to a value close to 1.2 (Supplementary Note 1 and Fig. S2). Even though both SNR and PSF size are expected to be slightly degraded for this lower NA, this permits to give a low-bias estimate of δ_3D_ with a reasonable compromise on the loss of signal, as long as the tilt angle of the fluorophore off-plane orientation η does not surpass 45° (Supplementary Note 1 and Fig. S2). The use of a lower NA in 4polar-STORM offers two other important advantages. First, it provides a higher sensitivity of the (*P*_0_, *P*_45_) ratiometric factors to the *δ* parameter (Supplementary Fig. S2). Second, the use of a NA lower than the critical angle numerical aperture (typically around 1.3) makes the *δ* measurement independent from the distance between the emission dipole and the coverslip interface (Supplementary Fig. S2). At distances lower than the emission wavelength λ, the emission coupling to super-critical angles influences the intensity pattern at the back focal plane of the microscope^37^ but also its polarization. This distance is however generally not known. In this work, to provide a sensitive and minimally biased measurement of the orientation parameters (*ρ*, *δ*), a detection NA of 1.2 is used by decreasing the size of the back focal diameter in the intermediate Fourier plane of the detection path of the microscope (see Methods).

In practice, the retrieval of the orientation parameters (ρ,δ) consists in detecting first the 2D position of single molecules in each of the polarization channels, previously corrected for polarization split efficiency and polarization leakages of the four channels (Supplementary Note 2) and registered (Supplementary Note 3). Each molecule is associated with its pairs in all polarization channels, where polarized PSF amplitudes and sizes are deduced from a Gaussian fit in order to calculate its intensities $\left({I}_{0},{I}_{45},{I}_{90},{I}_{135}\right)$ (Supplementary Note 3). We note that if molecules have a well-defined orientation (low wobbling) and are oriented off-plane, their PSF will enlarge and deform towards a donut-shape^6^. Even though this effect is minimized when molecules wobble^38^, the PSF fit has to allow for large ranges of sizes, since the measured PSF size for tilted molecules can be larger than for in-plane molecules. Once intensities are determined in the four-polarization channels, the (*P*_0_, *P*_45_) ratiometric factors are deduced and used to extract both ρ and δ parameters per molecule (Supplementary Note 1). This calculation uses a relatively simple model, for computational speed reasons, which is shown to be very close to a complete model characterization accounting for the inversion of the propagation equations (Supplementary Note 1 and Figs. S2, S3).

To estimate the expected precision on both ρ and δ parameters in 4polar-STORM detection conditions, we ran Monte Carlo simulations over generated images (see Methods and Supplementary Fig. S4a–c). Different signal-to-noise situations were considered accounting for the camera noise and the presence of background, using as a signal level the total intensity *I*_*T*_, i.e. the integrated signal over the whole single molecule’s PSF as estimated in the 4polar-STORM detection algorithm, summed over all four channels. Gaussian-shape PSFs were considered for this simulation, which is close to the expected situations studied in this work (Supplementary Fig. S4d). We first consider molecules lying in the sample plane (η = 90°). At relatively high-intensity conditions (*I*_*T*_ > 3000 photons) and in the absence of background, we expect a precision of a few degrees on ρ for δ < 140° (Fig. 1c). This error increases for very large wobbling values as expected from the increased uncertainty in determining the mean orientation under conditions of close-to-isotropic orientation fluctuations. The precision on δ stays below 10° for high intensity conditions (Fig. 1d). At lower intensities and in the presence of background, the precision on ρ and δ decreases as expected (Fig. 1c, d), reaching similar performance to other polarized microscopy methods in the context of in-plane oriented molecules^9,10,17,39^. The presence of background also decreases the accuracy of the retrieved parameters, especially at extreme (low or high) δ values (Supplementary Fig. S10). At a signal of 1500 photons with no background, an accuracy of δ better than 10° is ensured over the whole δ range. If the background increases to 60 photons/pixels, an accuracy better than 10° is ensured only for δ values between 60° and 160°. Note that as soon as molecules are tilted off-plane, both error and bias on (ρ,δ) increase more dramatically at high tilt (η < 45°) and extreme δ values, an effect which is exacerbated at low intensity/high background conditions (Supplementary Figs. S11, S12). This effect and its quantitative extent are in line with previous theoretical analyses that showed an intrinsic dependence of this bias on the signal to background ratio^23^. Our simulations show overall that within the range of δ between 80° and 150°, typical detection conditions of 1500 photons and a background level of 60 photons/pixel, ensure precise and accurate evaluation of the orientational parameters (ρ,δ) (within a ~10° limit at maximum) for molecules lying close to the sample plane (η > 45°). As expected, the presence of background is also associated with a degradation of the localization precision: under 4polar-STORM detection conditions, a background of 60 photons/pixel leads to a localization precision below 20 nm (Supplementary Fig. S13).

We also tested the capacity of the 4polar-STORM method to retrieve orientational parameters for molecules which are in close proximity in an image. Monte Carlo simulations show that in detection conditions of 1500 photons and a background of 60 photons/pixel, orientational parameters can still be retrieved with low bias (a few degrees) for molecules as close as five pixels apart (650 nm) (Supplementary Fig. S14), which corresponds to a density of 2.4 PSFs/μm^2^. At higher intensity levels, this distance can decrease down to 4 pixels (520 nm) (Supplementary Fig. S14). These high spot-density conditions can be reached thanks to the relatively reduced PSF size of the polarization-splitting method. At last, we tested the robustness of the 4polar-STORM method to deformations of the single molecules’ PSFs, possibly due to strong off-plane orientations, aberrations, or defocusing. Deformations can produce a slight enlargement of the PSF size or anisotropic shapes of the PSFs with two different radii along two orthogonal directions, which can potentially alter intensities estimation. Both effects, tested up to a radius expansion from 1.3 pixels (169 nm) in one direction to 1.7 pixels (221 nm) to its perpendicular direction, induce no variation on the ρ retrieval and tend to slightly bias the retrieval of δ (with variations <10°), even in the presence of noise and background (Supplementary Fig. S15).

Using this theoretical framework, we aimed at using 4polar-STORM imaging to measure the nanometer-scale actin filament organization in complex assemblies in cells labeled with fluorophore-conjugated phalloidin molecules^40,41^. To evaluate the fluorophore orientation behavior in a flat, single actin filament with a well-defined direction, we started by reconstituting single actin filaments immobilized on a glass surface (see “Methods”), as schematically represented in Fig. 1a. This provides a reference for later deciphering actin filament organization in unknown, more complex assemblies. Figure 1e shows the results obtained on isolated actin filaments labeled with Alexa Fluor 488 (AF488)-phalloidin, represented as sticks whose color is the wobbling angle δ and whose orientation, relative to the horizontal axis, is the angle ρ. AF488 molecules are visibly oriented along the actin filament axis, and exhibit a non-negligible degree of orientational flexibility δ. In order to provide high precision estimates, we systematically threshold the experimental data to a minimum intensity of 1100 photons, and to a maximum localization precision value σ_loc_ (estimated from lower bounds) of 0.15 pixels (20 nm). Avoiding thresholding is possible if necessary to increase the number of analyzed molecules, at the expense of a lower precision on the retrieved parameters. Typical statistics obtained on δ and ρ in straight segments of actin filaments are plotted in Fig. 1f, g respectively. The distribution of Δρ = ρ − <ρ> (e.g. ρ relative to its average <ρ> in the considered region) is characterized by a standard deviation σ_Δρ_ which represents the range of orientations explored by single AF488 molecules with respect to the actin filament; σ_Δρ_ also gives a measurement of the fluorophore tilt angle with respect to the filament axis ($\xi$ angle in Fig. 1a). Figure 1f, g shows that AF488 labels are, on average, oriented along the actin filament direction with a tilt angle of about $\xi$ = 20° with respect to the actin filament axis, and a large wobbling angle of δ = 85°–90°. This is consistent with the fact that the phalloidin-fluorophore conjugate exhibits a very small size (on the order of 1 nm) and a structure that fits in the groove formed by three neighboring G-actin monomers^42^, while leaving some space for mobility for the fluorophore. As previous studies have suggested, this degree of mobility is expected to originate mostly from the structure of the fluorophore itself and its precise conjugation to the phalloidin moiety, AF488 being among the least wobbly fluorophores among STORM dyes^5^. Despite the existence of this wobbling, the distribution of Δρ is a key information for exploring molecular organization along actin filaments.

**Fig. 2:**
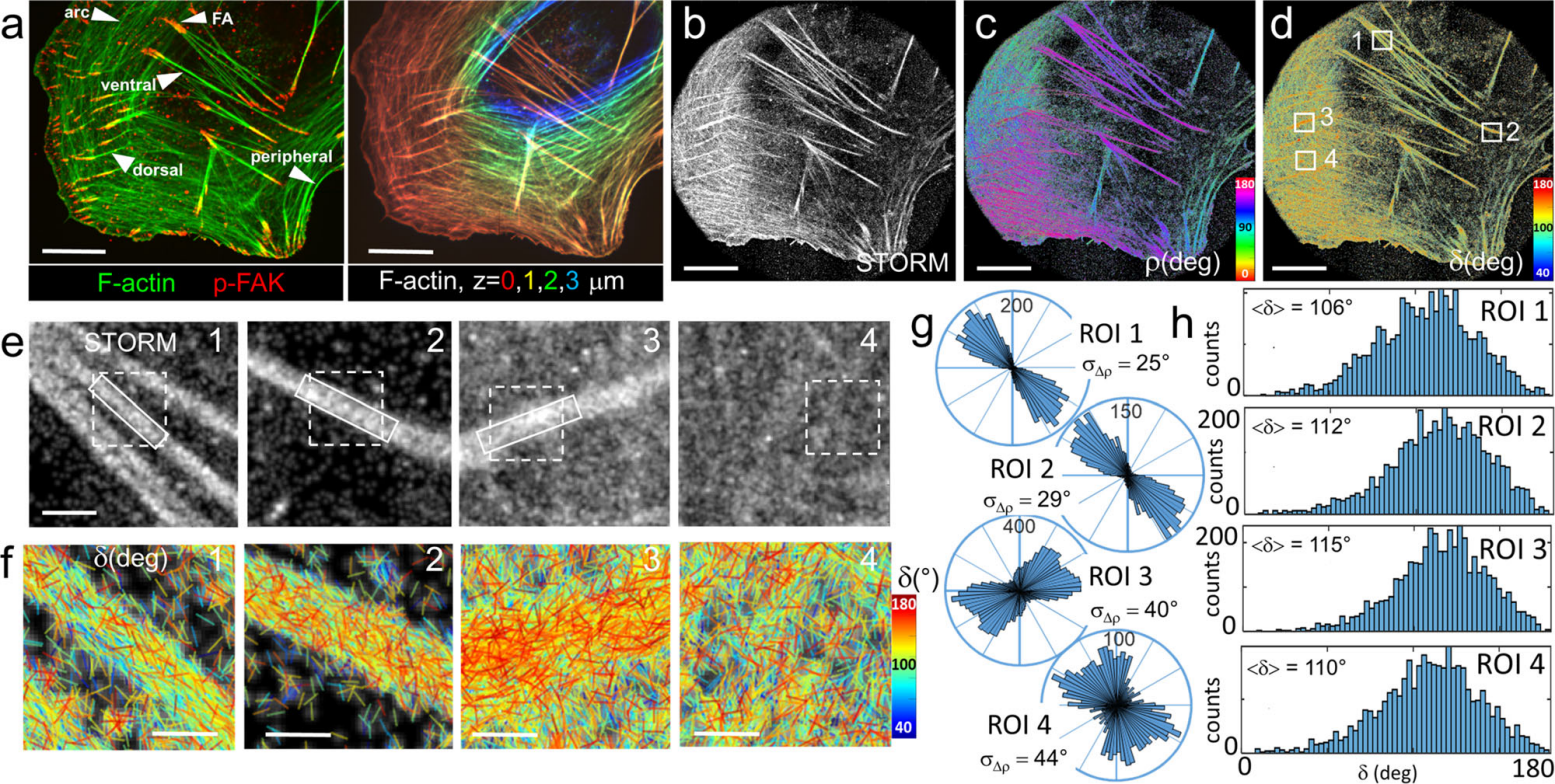
4polar-STORM imaging of actin filament organization in fixed U2OS cells. **a** Left: Spinning-disk fluorescence image of a U2OS cell (green, AF488-phalloidin-labeled F-actin; red, p-FAK). White arrows indicate focal adhesions (FAs) and the types of stress fibers (SFs) of interest. Right: z-stack mage of F-actin, z is color-coded as indicated. **b** Large field of view single-molecule (AF488) localization STORM image of the same cell. **c** Corresponding 4polar-STORM ρ stick image with color-coded orientation measurements. **d** Corresponding 4polar-STORM δ stick image with color-coded wobbling angle measurements. **e** STORM of zoomed regions of interest (ROI) (squares in (**d**)). ROI 1, ventral SF; ROI 2, FA; ROI 3, dorsal SF; ROI 4, meshwork. **f** δ stick images of zoomed regions (dashed squares in (**e**)). 4polar-STORM images were repeated independently on three different samples (3–5 cells per sample) with similar results. **g** Polar-plot histograms of ρ in the rectangle region indicated in (**e**) (whole region for ROI 4), with corresponding standard deviation σ_Δρ_ values of Δρ = ρ − <ρ>. **h** Histograms of δ values for ROIs 1–4, with corresponding values of <δ> over all measured molecules. For all images, intensities are thresholded above 1100 photons and localization precisions are thresholded below 0.15 pixels (σ_loc_ < 20 nm). Scale bars (**a**–**d**), 7 μm; (**e**), 800 nm; (**f**), 260 nm. Source data are provided as a Source Data file.

Using the same labeling approach, we next investigated the organization of actin filaments in structures that are expected to be highly organized in cells, notably actin stress fibers (SFs). We focused on ventral stress fibers, both ends of which associate with focal adhesions (FAs) on the ventral surface of the cell; on dorsal stress fibers, with one end associating with FAs on the ventral surface and the other end extending upwards toward the dorsal cell surface; and on meshworks (Fig. 2a). 4polar-STORM images of an actin-stained cell are shown in Fig. 2b–d, which depict, respectively, the single-molecule localization image (STORM), the ρ and the δ images from the same cell. A minimum intensity threshold of 1100 photons and a maximum localization precision value σ_loc_ of 20 nm are applied to all data in order to exclude estimates that lead to low precision and inaccuracy in the parameters’ determination. While the distributions of the retrieved (ρ,δ) parameters are not considerably modified without this threshold (Supplementary Fig. S16), it ensures that statistical analyses on ρ are performed with a few degrees precision. In the measured cells, this threshold keeps about 50–60% of the total number of detected molecules, allowing statistics on a few thousands of molecules per region selected (Supplementary Fig. S16). In well isolated thin ventral SFs, AF488 is oriented predominantly along the SF direction (ROI 1 in Fig. 2e–g), similarly to what was observed in single actin filaments (Fig. 1). This observation confirms that these SFs are made of highly parallel filaments, which is expected from the tight crosslinking of these structures^32^. Many molecules exhibit however larger δ values than the ones measured in single filaments. This is even more pronounced in dorsal SFs and ventral SF parts close to FAs (ROI 2,3 in Fig. 2e, f). In these regions, ρ distributions are wider (Fig. 2f, g) and δ distributions are enriched in larger δ values (Fig. 2h, compare ROIs 2–3 with ROI 1). This trend was observed for multiple SFs in all measured cells (Supplementary Figs. S17, S18). At last, in seemingly homogeneous meshworks, AF488 orientations ρ exhibit much larger distributions (ROI 4 in Fig. 2e–g).

**Fig. 3:**
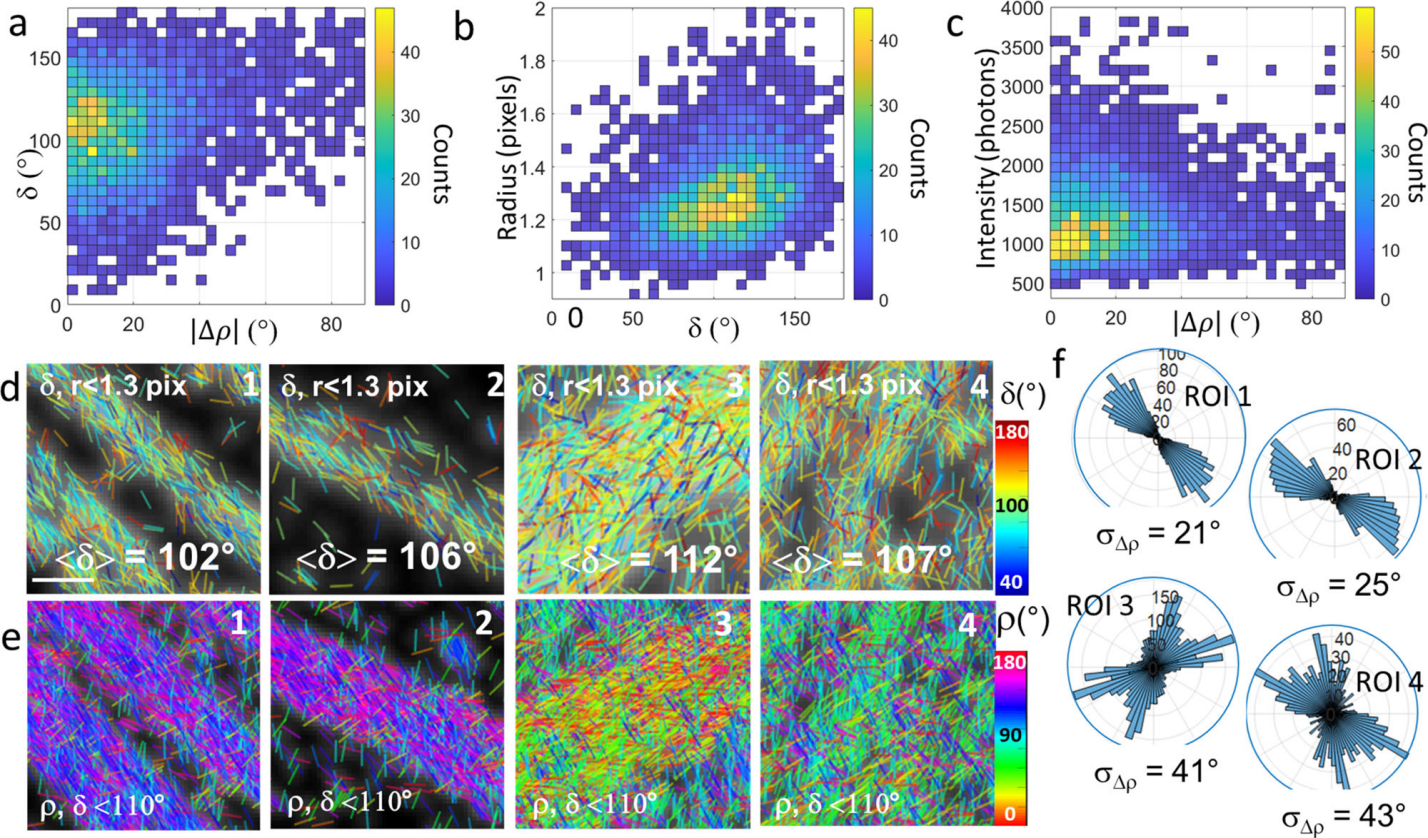
Influence of the detection parameters on 4polar-STORM imaging. **a** 2D histogram of (|Δ*ρ*|, δ) values obtained in single AF488 molecules (all detected intensities) present in ROI 1 (rectangle in Fig. 2e), with $\triangle \rho =\rho -\left\langle \rho \right\rangle$. **b** 2D histogram of (PSF radius, δ) values of the same region. **c** 2D histogram of (intensity, |Δ*ρ*|) values of the same region. **d** Same 4polar-STORM images as in Fig. 2e, f, depicting δ sticks only for molecules for which *r* < 1.3 pixels (169 nm). The corresponding average <δ> over all measured molecules (present in the rectangle for ROIs 1–3 or the whole region for ROI 4 as in Fig. 2e) are shown. 4polar-STORM analyses were repeated independently on SF ROIs of three different samples (about 3–5 cells per sample) with similar results. **e** Same ROIs as in (**d**) showing ρ stick images only for molecules with δ < 110° (all PSF radii). **f** Corresponding polar-plot histograms of ρ for regions indicated in (**e**). The corresponding σ_Δρ_ values are shown. Scale bar (**d**, **e**), 800 nm. Source data are provided as a Source Data file.

To understand the presence of large δ values and wide ρ distributions in SFs, we investigated possible correlations between these two parameters. We found first that in all observed SFs, large δ values generally correlate with a wide distribution of ρ values, as illustrated in Fig. 3a. The measured ρ angles seem to deviate most from their mean when δ is the highest. This trend is observed not only at the single-molecule level, but also in data averaged over several ROIs of SFs in cells (Supplementary Fig. S19a). This behavior could physically correspond to molecules that are freely, isotropically rotating, but we exclude such an effect. First, no free phalloidin-fluorophore conjugates are expected given the high affinity of phalloidin for actin filaments. Second, higher wobbling angles are not present in single actin filaments and thin actin bundles. We exclude also a sensitivity of wobbling to actin filament packing within bundles, considering the small size of phalloidin-AF488; single actin filaments do not present packing-related constraints and did not exhibit high wobbling angles. Our hypothesis for the observed high δ values is the presence of actin filaments oriented in 3D out of the sample plane (off-plane η angle in Fig. 1a), which are expected to lead to an overestimation of δ (Supplementary Figs. S11a, S12a). The use of a relatively low detection NA of 1.2 minimizes the bias induced by 3D orientations as compared to higher NA conditions, but does not entirely exclude this effect for highly tilted molecules. Typically, wobbling molecules with δ_3D_ = 100°, η = 45° lead to a measured δ ~ 130° (with no significant variations due to noise and background) (Supplementary Figs. S11a). 3D oriented molecules also naturally introduce a bias on ρ at low intensity/high background conditions, especially at high δ (Supplementary Figs. S11b, S12b). In addition to this intrinsic bias, the 2D geometrical projection of molecular distributions along a tilted filament increases the estimated width σ_Δρ_. Typically a distribution of σ_Δρ_ = 10° for an in-plane filament increases up to a measured σ_Δρ_ ∼ 33° (40° in the presence of noise) when this filament is tilted off-plane by 40° (Supplementary Fig. S20). We also simulated how off-plane orientations affect the precision on the (ρ,δ) parameters. The presence of noise and background decreases the precision on ρ for off-plane orientations; however, this error stays below the limits of the induced biases (Supplementary Figs. S11d, S12d). The precision on δ is not affected by off-plane orientations (Supplementary Figs. S11c, S12c).

To test the hypothesis of possible filaments oriented off-plane in the observed ROIs, we investigated the correlation of δ and ρ with the single-molecule detection parameters, in particular their intensity, which is expected to decrease when fluorophores are tilted off plane due to their lower photo-excitation, and the PSF radius, which is expected to increase when molecules are either tilted off-plane or out of focus. Highest δ values are seen to correspond mostly to high PSF radii populations (Fig. 3b and Supplementary Figs. S19b, S21), while high-Δρ values correlate essentially with low intensity and high PSF radii (Fig. 3c and Supplementary Figs. S19c, S22). Thresholding intensities and σ_loc_ values as above and keeping PSF radii below 1.3 pixels (169 nm) leads to a net reduction of the populations of high δ values (Fig. 3d; compare with Fig. 2f) (see also Supplementary Figs. S17, S21, and S22). Since the high PSF-radius populations likely correspond to molecules tilted off plane and/or possibly positioned at slightly different heights, we therefore attribute a large part of the observed populations with high δ (and wide distributions of Δρ) to filaments that are tilted off-plane with respect to the sample coverslip. Such tilted filaments are found, as expected, more frequently at the FA sites and in dorsal SFs, both of which are expected to contain off-plane filament populations, in contrast to ventral SFs, which lie in the plane of the substrate (Fig. 2a). Note that the PSF radius analysis described above helps understanding the origin of the observed high δ population in SFs, it is not used as a thresholding parameter in what follows.

Following the relation between measured δ and off-plane angles, we can estimate the single-molecule’s off-plane angle η qualitatively from the theoretical δ vs η bias dependence, which is not significantly sensitive to noise and background (Supplementary Figs. S11, S12). Typically an initial wobbling angle of δ_3D_ = 100° would lead to a measured value of δ = 110° for η = 65°, which represents a tilt of 25° relative to the sample plane (Supplementary Figs. S11a). For actin labeled with AF488-phalloidin, δ values measured in in-plane single filaments give an estimate of the in-plane wobbling angle of about δ_3D_~ 90° (Fig. 1f). It is therefore a reasonable assumption to state that populations with δ > 110° observed in Fig. 3a is attributed to filaments tilted off plane by more than 30° from the sample plane (Supplementary Fig. S12a). Thus, selecting δ values with δ < 110° is a way to select in-plane actin filaments, which organization in SFs can be estimated without a strong bias on δ and on σ_Δρ_ (Supplementary Fig. S11 and simulated tilted distribution in Fig. S20). Using this criterion, the parameter σ_Δρ_ can be used as a quantitative estimate of in-plane actin filament organization in selected regions (Fig. 3e). σ_Δρ_ is seen to decrease in SFs for δ < 110° (Fig. 3f, ROIs 1 and 2, compare with Fig. 2f, h), reaching in some regions the σ_Δρ_ values of single actin filaments (Fig. 1g) (see also Supplementary Fig. S18), thus showing now highly aligned actin filaments in ventral SFs and FAs. Regions in dorsal SFs and meshworks depict now more clearly populations of actin filaments crossing each other, as expected (Fig. 3e, f, ROIs 3 and 4, compare with Fig. 2f, g). This behavior has been observed in all cell regions (see Supplementary Fig. S23). Importantly for the population δ < 110°, the estimated σ_Δρ_ is found to be of similar values independently of the PSF radius size and of low sensitivity to the intensity and σ_loc_ thresholding (Supplementary Figs. S18, S24). This is due to the intrinsic low bias on the measured parameters induced by the selection of the in-plane population, and to the correlation between δ and the detection parameters described above. In general, the detection parameters do not strongly influence the in-plane distribution of ρ as long as the population δ < 110° is considered.

Our analysis emphasizes two possibilities that the 4polar-STORM method offers for the investigation of actin filament organization. First, the wobbling of single fluorophores can be used for the quantification of their off-plane tilt orientations, exploiting the degree of bias even at a slightly reduced detection NA. Second, the lowest wobbling values obtained in a population of fluorophores can be used to select in-plane populations of actin filaments and study their organization in a quantitative manner. The selection of populations with δ < 110° is the condition for a quantitative retrieval of accurate and precise molecular organization estimation in the sample plane. The downside is a reduction of the number of selected molecules; nevertheless, thousands of molecules are still detected for statistical analysis within μm-size ROIs (Supplementary Fig. S18). In what follows, we exploit these considerations to quantitatively compare actin filament organization in different structures of the cell.

### Actin filament organization imaging by 4polar-STORM in-plane selection

**Fig. 4:**
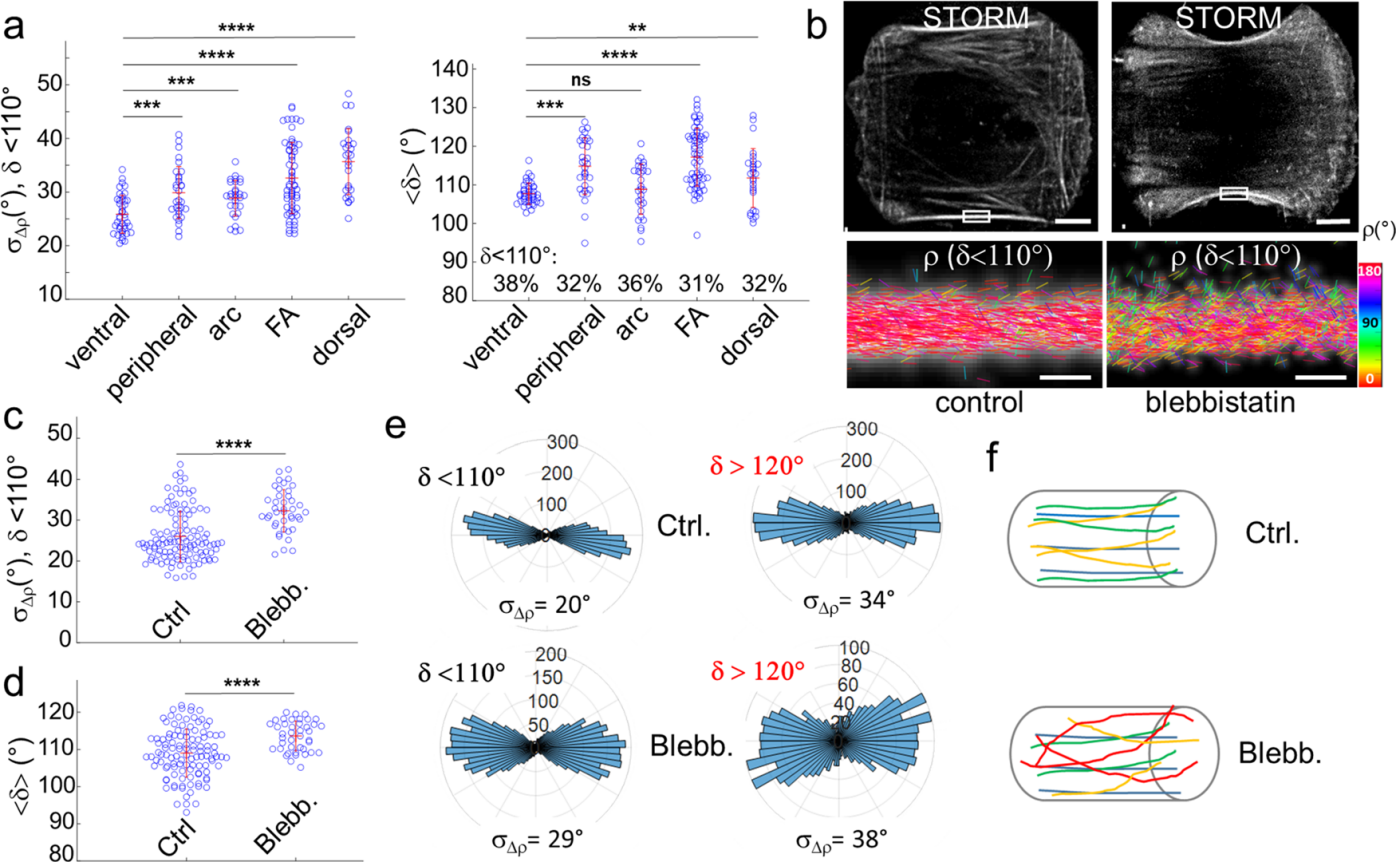
4polar-STORM imaging of actin filament organization in different types of stress fibers. **a** (left) σ_Δρ_ (δ < 110°) and (right) <δ> (all molecules, the percentage of the δ population with δ < 110° is indicated), measured in ROIs on different types of SFs. Eight cells were examined, giving a total number of measured ROIs of: *n* = 42 (ventral), *n* = 32 (peripheral), *n* = 30 (arc), *n* = 63 (FA), *n* = 28 (dorsal). The red bars represent mean values ± standard deviation. Statistical significance: ns (*p* > 0.05); ** (*p* < 0.01); *** (*p* < 0.001); **** (*p* < 0.0001) (two-sided unpaired two-sample T-test). *p* values from top bar to bottom, compared to the ventral SF population: (Left) *p* = 6.3e−12, 3.6e−8, 6.2e−4, 1.2e−5, 1055 molecules measured on average per ROI; (right) *p* = 2.6e−3, 8.2e−12, 0.3, 2.6e−7, 2994 molecules measured on average per ROI. **b** top: STORM images of control and blebbistatin-treated U2OS cells on micropatterns (see Methods). Scale bars, 6.5 μm; bottom: 4polar-STORM ρ stick images of zoomed regions (rectangles in the STORM images). Scale bars, 500 nm. Images were repeated on four different cells per condition, with similar results. **c**, **d** σ_Δρ_ and <δ> measured in four cells per condition (control, blebbistatin-treated), using in total *n* = 87 ROIs in control cells and *n* = 43 ROIs in treated cells. **c** σ_Δρ_ values (δ < 110°), *p* = 3.5e−8, 2333 molecules measured on average per ROI, (**d**) <δ> values (all δ selected), *p* = 4.1e−5, 6376 molecules measured on average per ROI. **e** Polar-plot histograms of ρ in the zooms shown in (**b**), for δ < 110° (left) and δ > 120° (right). The corresponding σ_Δρ_ values are shown. **f** Schematic representations of the SFs observed in control and blebbistatin-treated conditions, showing highly aligned actin filaments in 2D (top) and disorganized filaments in 3D (bottom). Source data are provided as a Source Data file.

We first analyzed actin filament organization in different types of SFs in cultured U2OS cells on unpatterned or micropatterned coverslips (see Methods), quantifying σ_Δρ_ for molecules with δ < 110°. In what follows, the PSF radius of single molecules is not thresholded, for the reasons explained above. Regions defining ventral, peripheral, dorsal SFs as well as transverse arcs and FAs are measured by 4polar-STORM. We combine phalloidin stainings with immunostainings for a FA protein, the phosphorylated form of focal adhesion kinase, p-FAK, in order to define the different types of SFs based on their association with FAs^43,44^ (Fig. 2a). A z-stack of the cell further allows us to visualize off-plane tilted structures (Fig. 2a). While ventral, peripheral and arc SFs are lying in the sample plane, dorsal SFs are the most tilted ones. σ_Δρ_ values were measured in rectangular ROIs of typically (0.5–1 μm) × 100 nm in size. σ_Δρ_ exhibits very large distributions, due to variations in actin filament organization among SFs within a given cell and across cells (Fig. 4a). Among the measured SFs, ventral SFs exhibit the highest filament alignment (lowest σ_Δρ_ value), with the average σ_Δρ_ close to that measured in single filaments (<σ_Δρ_> ~ 25°). σ_Δρ_ values for transverse arc SFs are slightly higher, suggesting the presence of less well-aligned actin filaments or/and filaments crossing each other. Such organization is fully consistent with the proposed mechanisms of transverse arc assembly, involving both the progressive fusion and alignment of actin filament fragments and myosin filament stacks, and connections of forming arcs with dorsal SFs^45–47^. σ_Δρ_ values for peripheral SFs are, surprisingly, even larger, suggesting that peripheral SFs contain a larger population of actin filaments in various directions. This observation correlated with the fact that the measured peripheral SFs were often thicker than the measured ventral ones, which suggests that thicker SFs contain more various actin filaments directions. This hypothesis is in line with observations of actin bundles fusing with or splitting from peripheral fibers and with recent work showing that peripheral SFs and the cortical meshwork form a continuous contractile network^48^. FAs, regardless their association with ventral or dorsal SFs, depict a much wider distribution of σ_Δρ_ values, containing both highly aligned actin filaments and actin filaments in mixed orientations. We hypothesize that this organization reflects the dynamical nature of FAs, whose precise assembly and maturation depend on the local mechanical environment^49,50^. At last, dorsal SFs exhibit the highest σ_Δρ_ values with < σ_Δρ_> ~ 35°. We attribute these high values to the very nature of dorsal SF assembly involving extensive interconnections along their length with transverse arc SFs^43,44^. Depicting all < δ> values measured in all ROIs confirms that peripheral SFs, FAs and dorsal SFs contain the largest population of filaments tilted off plane, with the lowest proportion of single molecules with δ < 110° (Fig. 4a).

Our measurements show that ventral SFs exhibit the highest actin filament alignment among the different SF types. Peripheral SFs have been reported to be under a larger mechanical strain than ventral SFs^51^; however, parameters other than actin filament alignment, such as the macroscopic morphology of these two SFs types (diameter, length, shape) and the 3D expansion of their organization could play a role in their mechanical properties. The precise relation between the molecular-scale actin organization and the macroscopic scale of mechanical properties is still poorly understood. To bring quantitative elements to this question, we probed how actin filament organization directly depends on contractility and thus on the mechanical tension of SFs. To this end, we treated U2OS cells with blebbistatin, a drug that inhibits myosin II activity and therefore acto-myosin bundle contractility. To minimize dispersions due to SF type and morphology heterogeneities, we performed this experiment on cells adhering to H-shaped micropatterned substrates of a well-defined size, concentrating on peripheral SFs (see Methods). Blebbistatin treatment leads eventually to the dissociation of the contractile peripheral SFs^52^ (also seen in our data). Blebbistatin concentration and incubation time were thus kept low enough to induce a loss in contractility, evidenced by the concave shape of relaxed SFs, while preserving the apparent macroscopic integrity of the SFs (Fig. 4b). The single-molecule localization image alone (STORM, Fig. 4b) cannot inform us on the underlying actin filament organization in the relaxed SFs. 4polar-STORM measurements, however, show a slight decrease of actin filament alignment, with a statistically significant increase of <σ_Δρ_> from 26° to 32° (Fig. 4c), and also an enrichment of 3D oriented filaments as seen from the increase of <δ> (Fig. 4d). Contractility loss induced by myosin II inactivation is therefore correlated to a decrease of actin filament organization which is also visible from the 4polar-STORM ρ images (Fig. 4b). Representative distributions of single-molecule orientations ρ in control and blebbistatin-treated SFs are shown in Fig. 4b and quantified in Fig. 4e, for both in-plane (δ < 110°) and off-plane (δ > 120°) filament populations. The spreading of the distributions induced by blebbistatin confirms a decrease in actin filament alignment and a concomitant increase in 3D oriented filament populations as schematically represented in Fig. 4f. 4polar-STORM measurements thus reveal that actin filament organization in SFs is sensitive to acto-myosin contractility, with myosin II inhibition inducing a loss in actin filament alignment at the nanometer-scale. These results show that actin filament organization is an important parameter in the build-up of mechanical tension in SFs.

**Fig. 5:**
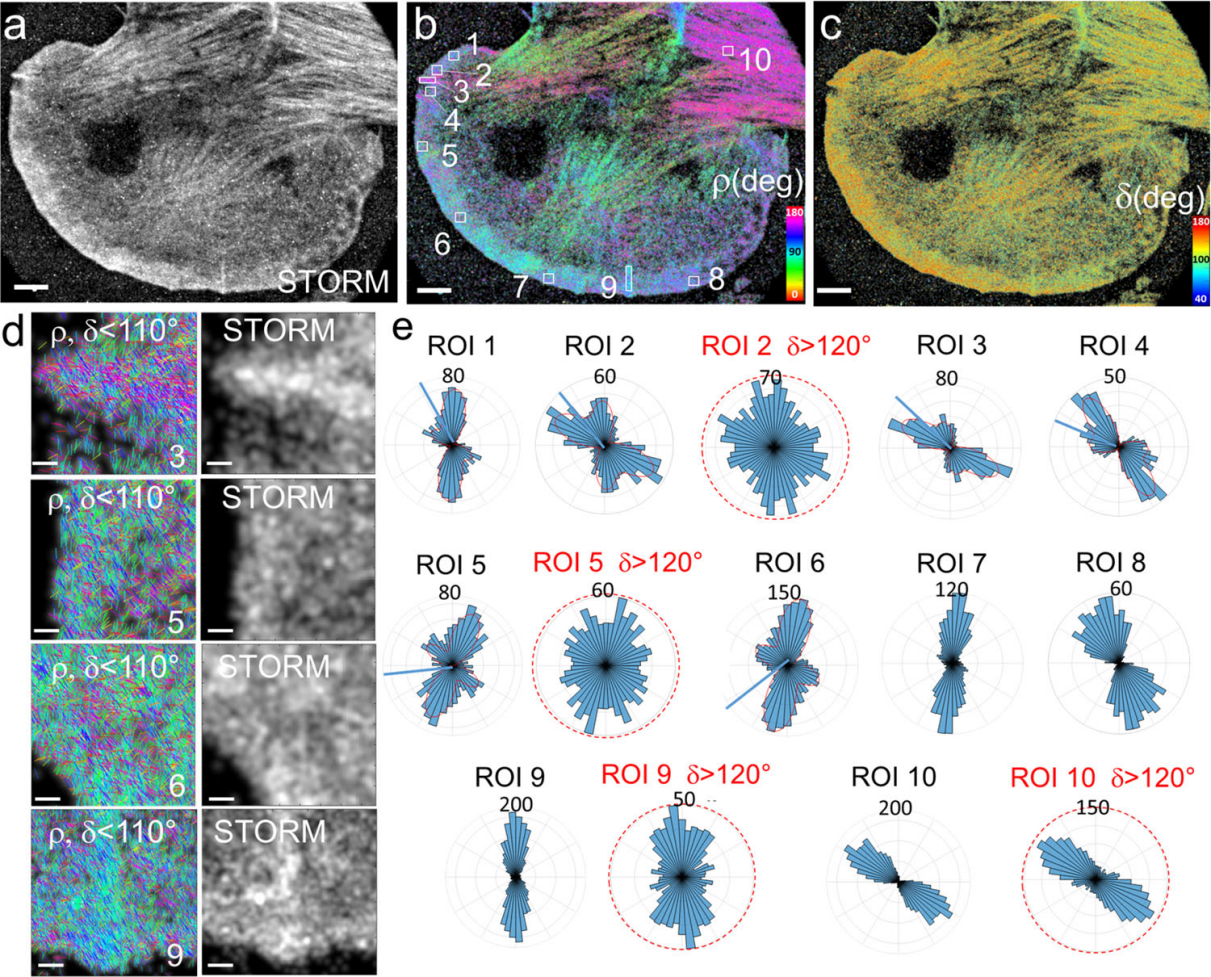
4polar-STORM imaging of actin filament organization in lamellipodia. **a** Single-molecule localization STORM image of a B16 cell labeled with AF488-phalloidin. **b** Corresponding 4polar-STORM ρ stick image with color-coded orientation measurements. **c** 4polar-STORM δ stick image with color-coded wobbling angle measurements. 4polar-STORM images were repeated independently on four different cells with similar results. **d** Examples of ρ stick images showing molecules with δ < 110° and corresponding STORM images in selected ROIs (squares in (**b**)). ROIs 1–9, regions in the lamellipodium; ROI 3,9, microspikes; ROI 10, SF. **e** Polar-plot histograms of ρ for the regions shown in (**b**). The condition δ < 110° is used, except for red-circled histograms for which δ >120° molecules are selected. The red lines in ROIs 1–6 are fits of the ρ histograms that resemble bimodal distributions. The blue lines in these same ROIs correspond to the normal direction of the cell membrane in the respective ROI. Angle between the two peaks around the membrane normal direction, obtained from the fits: ROI1 (60°), ROI2 (59°), ROI3 (50°), ROI4 (52°) ROI5 (70°), ROI6 (73°). Scale bars (**a**–**c**), 4 μm; (**d**), 260 nm. Source data are provided as a Source Data file.

At last, we investigated actin filament organization in dense networks at the leading edge of B16 melanoma cells (Fig. 5a). We concentrate in particular on the lamellipodium, which spans a few micrometers at the cell border^36^. Again, the single-molecule localization images alone (STORM in Fig. 5a, d) do not provide any information on how single AF488-phalloidin molecules orient with respect to one another and thus on the nanometer-scale organization of the labeled actin filaments; the very high filament density in these networks makes it even more challenging to decipher the precise arrangement of filaments. Remarkably, the contour of the cells imaged by 4polar-STORM shows different (ρ,δ) distributions than in the rest of the cell (Fig. 5b, c). Within the first hundreds of nanometers from the cell contour, single molecules exhibit wide distributions of orientations (ρ) and high wobbling (δ) values, signatures of more disorganized, off-plane actin filament populations. Selecting only in-plane filaments (δ < 110°) shows that actin filaments in these dense networks are not oriented isotropically, but that there are visible preferred orientations which cannot be detected in the single-molecule localization images alone^24^ (Fig. 5d and quantification in Fig. 5e). Single-molecule orientation distributions at the cell border (ROIs 1–8 in Fig. 5b) reveal in general the presence of two main populations with different contributions (Fig. 5e). Generally, one of the two orientation populations appears to predominate (ROIs 1,6–8). However, in some regions (ROIs 2,5), the two populations resemble a bimodal distribution, which becomes more isotropic when off-plane molecules are considered (δ > 120°). This behavior has been observed in all cells measured (see another example in Supplementary Fig. S25). We note that these distributions are very different from those found in SFs, which exhibit much narrower distributions with a preferred orientation, even when considering off-plane molecules (ROI 10). At last, narrow distributions are found in microspikes (ROIs 3,9), which is a signature of parallel actin bundles within the lamellipodium, despite their close proximity to bimodal distributions (see ROIs 2 and 4 which surround ROI 3).

The angle between the two peaks of the observed bimodal distributions is close to 50–70°, with some variations along the cell contour, and points towards the normal to the membrane contour (Fig. 5e). This is reminiscent of observations made by EM where actin filaments display a bimodal angular distribution, with filament orientations peaking at 35° and −35° with respect to the direction of membrane protrusion^34–36^. This so-called dendritic organization was attributed to the angle imposed by the Arp2/3 complex involved in actin filament branching in those regions^36,53^. Bimodal orientation distributions in 4polar-STORM images were present in ROIs from hundreds of nanometers to micrometers sizes, and were variable depending on the region of the cell contour, emphasizing the importance of a large field of view observations.

The variations in the precise distribution of actin filament orientations at the cell edge, and the non-negligible presence of 3D orientations, are consistent with recent findings in the literature based on EM and modeling^54^. A range of angular distributions of actin filament orientations has been evidenced, which appears to be more complex and heterogeneous than a pure bimodal distribution with peaks positioned at the 70° branching angle^55^. This angular spread depends on the cell mechanical load^56^, and the precise geometry of filament assemblies, not necessarily pointing towards the membrane normal, depends on the modulation of the protrusion rate^57,58^. Additionally, actin filament organization within the lamellipodium sheet is known to extend in 3D, with possibly different organizations at the cell surface and in the upper layer as evidenced recently in studies using 3D super-resolution localization microscopy^24^ or cryo-tomography EM^58^. The 4polar-STORM results, which do not differentiate between specific actin layers, suggest the co-existence of both preferred angular distributions of actin filament orientations and more isotropic populations of 3D oriented filaments.

## Discussion

The 4polar-STORM permits to evaluate quantitatively the organization of single molecules in 2D. A detailed theoretical analysis of the dependence of the orientation and wobbling parameters on different sources of bias, either originating from SNR conditions or physical features, such as 3D molecular orientations, is used to define appropriate detection conditions and analysis procedures for the minimally biased determination of molecular organization at the nanometer scale. It is important to note that the majority of polarization-split-based single-molecule experiments have been performed under high NA conditions so far. This prevents the physical interpretation of wobbling values and biases the determination of the underlying molecular organization^21,22^. Other alternatives such as PSF engineering necessitate more complex implementation and signal processing to achieve quantitative measurements, and can be challenged in dense samples where the spot-density is high^14,59^. With polarization splitting, we show that 4polar-STORM is performant at spot densities as high as 2.4 PSFs/μm^2^ in experimental signal and background conditions. Recently developed methods based on prior knowledge of sparsity and PSF modeling^59,60^, deep learning^61^, or spatial pooling^62,63^, show that there is room for optimization in this direction.

To highlight the potential of 4polar-STORM to measure molecular organization in complex protein assemblies, we measured the nanometer-scale organization of actin filament-based structures involved in the adhesion and motility of mammalian cells. We exploited the sensitivity of orientation and wobbling parameters to molecules lying off-plane to evidence the non-negligible contribution of 3D orientations in different populations of actin filaments, both in SF bundles and in the flat lamellipodium. Selecting in-plane filament populations evidenced the very high actin filament alignment in all types of SFs, in line with EM studies^30,31^, but also revealed differences in their nanometer-scale organization, consistently with their different modes of assembly and function in the cell. Thin 2D ventral and transverse arc SFs are made of highly aligned actin filaments, while thick peripheral SFs, off-plane oriented dorsal SFs, and FAs containing filament populations of mixed orientations, were seen to contain a non-negligible population of filaments with 3D off-plane orientations. Low doses of blebbistatin that inhibited myosin II activity in contractile peripheral SFs, while preserving their macroscopic integrity, resulted in a perturbation of the nanometer-scale organization of actin filaments, emphasizing the key role of myosin II in the organization of actin filaments in contractile SFs. Importantly, in-plane measurements of actin filament organization permitted us to investigate the organization of dense assemblies that is not accessible by single-molecule localization imaging alone, in particular in the lamellipodium at the leading edge of motile cells. 4polar-STORM imaging revealed that the actin filaments in the lamellipodial meshwork are not oriented randomly but that they organize in preferred angular distributions, including bimodal distributions previously observed by electron microscopy.

These results show altogether the added value of combining localization and orientation measurements in dense actin structures, and highlight the potential of 4polar-STORM to investigate the nanometer-scale organization of actin filaments in complex arrangements which are hardly accessible in standard optical super-resolution microscopy. In particular, the complex geometries of actin filaments close to the cell membrane and their link with local contractile and protrusive activity are poorly understood. Additionally, little is known on the organization of actin filaments in cell adhesion-mediating structures which play a large role in cell mechanics, in particular focal adhesions. Assessing the nanometric organization of actin filaments, and the contribution of 2D vs 3D oriented populations of filaments, at various stages of FA formation and maturation promises to permit us to understand how such complex molecular machineries assemble in order to sense, respond and adapt to mechanical stimuli. We note that while AF488-phalloidin is used in the present study, our method is fully compatible with a wide variety of labels as long as the orientational flexibility of the fluorophore is not very high, including in other spectral regions such as Atto633-phalloidin^5^ or silicon rhodamine-jasplakinolide (SiR-actin)^22,64^. Genetically encoded probes for actin filament orientation studies are also currently being developed^65^, using strategies which could be extended to other proteins of interest. Such approaches which are amenable to live-cell measurements promise to open new directions for orientational dynamics studies, combining for instance single-molecule orientation measurements with single-particle tracking PALM^66^. Wobbling measurements in the case of fluorescent proteins, whose size is typically comparable to the size of the labeled protein of interest, might also reveal packing-related constraints and could thus provide additional protein organization readouts. The methodology of 4polar-STORM is also compatible with two-color localization and orientation measurements and thus can provide insights into the functional interplay between the nanometric organizations of interacting partners; the link between conformational changes in activated integrins and actin filament remodeling is such an example. At last, 4polar-STORM is compatible with 3D localization schemes, including astigmatism^67^ or multiplane^68,69^ strategies, and can therefore be adapted for exploring the full 3D organization of a large variety of biological structures^70^.

## Methods

### 4polar-STORM optical setup

Measurements are carried out on a custom total internal reflection (TIRF) fluorescence microscope, whose detection path is adapted to retrieve four-polarization states of the single-molecule fluorescence images. The excitation light source is a continuous laser emitting at 488 nm (Sapphire 488LP-200, Coherent), whose beam is expanded by a telescope and polarized by a quarter waveplate (AQWP10M-580, Thorlabs) oriented such as to obtain close-to isotropic excitation in the sample plane. A set of mirrors reflect the beam towards the microscope, followed by a large focal length lens (*f* = 400 mm) to focus the beam in the back focal plane of the objective, to provide an illumination field of view with a diameter of about 100 μm. After the reflection on a dichroic mirror (DI02-R488, Semrock Rochester NY), the excitation light is focused onto the sample by an oil immersion objective lens (Plan Apo ×100, NA = 1.45, Nikon). The emitted fluorescence is collected back by the same objective lens, passes through the dichroic mirror and a band pass emission filter (FF01-525/40, Semrock Rochester NY). At the microscope exit a non-polarizing beam splitter separates the beam into two paths, each of them being built up with a 1x relay imaging telescope that uses two (f 150 mm) lenses. In the first path, a Wollaston prism (separation angle 5°, CVI Laser Optics) is placed at the back focal intermediate image plane, aligned for 0–90° polarization split. In the second path, a similar Wollaston prism is placed just after an achromatic half-wave plate (AHWP05M-600, Thorlabs), to provide 45–135° polarized images. The two beams are recombined by a mirror reflection of the first path, and refocused on the EMCCD camera detection plane (iXon Ultra 888, Andor, 13 μm pixel size), such as to fill the CCD chip with four polarized images. The size of the images is set by a diaphragm placed in the first image plane at the exit of the microscope (typical image field of view, 40 μm × 40 μm). In addition, two diaphragms are placed in intermediate planes conjugated to the back focal plane of the objective in order to reduce the detection numerical aperture to NA_det_ = 1.2. The imaging lens provides a total magnification of ×100, corresponding to a pixel size of 130 nm on the EMCCD. The stability of the focus throughout the measurement is ensured by a commercial system (Perfect Focus System, Nikon). For initial positionning, the sample is mounted on a *XYZ* piezo stage (Physik Instrumente). The acquisition parameters are controlled by a commercial imaging software (AndorSolis, Andor). For STORM imaging, a first fluorescence image is recorded with low intensity (~500 W/cm^2^, below STORM blinking conditions), ensuring the identification of relevant parts of the sample. The intensity is then raised to 5–8 kW/cm^2^, which is a typical level to provide a good compromise between signal level and blinking rate. The images are acquired at a rate of 100 ms/image, camera gain 300, with a total of about 30,000 images depending on the molecular density. The polarized path of the 4polar-STORM setup was calibrated as detailed in Supplementary Note 2. We ensured that all directions in the sample plane could be detected with identical efficiency and precision by rotating a polarizer at the back focal plane of the collection objective, analyzing the emission from isolated nanobeads (100 nm size yellow-green Carboxylate-Modified FluoSpheres, ThermoFisher Scientific F8803, immobilized on the surface of a poly-L-lysine coated coverslip and covered with a mounting medium Fluoromount, Sigma F4680). On average, ρ values were pointed with a few degrees precision with respect to the expected polarizer direction, and δ values range from 20° to 33°.

### Cell culture

4polar-STORM measurements were made in U2OS osteosarcoma cells (Figs. 1–4) and B16-F1 mouse melanoma cells (Fig. 5). Naive U2OS cells (gift from Flavio Maina, IBDM, France) were used for assessing the effect of blebbistatin. U2OS CA-MLCK cells (gift from Sanjay Kumar, UC Berkeley, USA) cultured in 0 ng/mL doxycycline were used for all other experiments. U2OS cells were originally from ATCC (HBT-96). U2OS cells were maintained in McCoy’s 5 A medium (ThermoFisher Scientific, 26600-080) supplemented with 10% fetal bovine serum (Biowest, S181H), 100 U/mL penicillin and 100 μg/mL streptomycin (Sigma, P4333) in a humidified incubator at 37 °C and 5% CO2. B16-F1 cells (gift from Klemens Rottner, Technische Universität Braunschweig, Germany) were cultured in DMEM (ThermoFisher Scientific, 41966-029) supplemented with 10% fetal bovine serum (PAA Laboratories, A15-102), 100 U/mL penicillin and 100 μg/mL streptomycin (Sigma, P4333) in a humidified incubator at 37 °C and 5% CO2. B16-F1 cells were originally from ATCC (CRL-6323).

### Cell preparation for 4polar-STORM. U2OS cells

24 mm-diameter high-precision (170 μm ± 5 μm) glass coverslips (Marienfeld, 0117640) were cleaned with base piranha (Milli-Q water, 30% ammonium hydroxide, 35% hydrogen peroxide at a 5:1:1 volume ratio) for 15 min, rinsed with Milli-Q water for 2 × 5 min in a bath sonicator, sonicated in 70% ethanol for 5 min, and air-dried before coating with fibronectin (SIGMA F1141) for 2 h at room temperature (RT) and at a final fibronectin concentration of 20 μg/mL in PBS. For experiments with micropatterned substrates, medium-size (1100 μm^2^) H-shaped patterns from CYTOO (10-900-00-18) were similarly coated with 20 μg/mL fibronectin. U2OS cells were seeded onto fibronectin-coated coverslips and allowed to spread for 5 h on micropatterned substrates or overnight on nonpatterned ones. Cells were fixed for 15 min with 4% formaldehyde (Electron Microscopy Sciences 15714) in cytoskeleton buffer (10 mM MES pH 6.1, 150 mM NaCl, 5 mM EGTA, 5 mM MgCl_2_, 5 mM glucose), washed for 2 × 5 min in PBS, then permeabilized and blocked in phosphate-buffered saline (PBS) containing 0.1% saponin and 10% bovine serum albumin (BSA) for 1 h at RT. Cells were incubated successively with primary rabbit anti-phospho-FAK antibodies at 1:200 (ThermoFisher Scientific 44-624G) and secondary donkey anti-rabbit Alexa Fluor 647-conjugated IgG secondary antibodies at 1:1000 (ThermoFisher Scientific A-31573) each for 1 h at RT and with three 10-min washes in-between antibody incubations. After five 6-min washes, cells were incubated with 0.5 μM Alexa Fluor 488 (AF488)-phalloidin (ThermoFisher Scientific A12379) in 0.1% saponin/10% BSA/PBS overnight at 4 °C in a humidified chamber. For 4polar-STORM measurements, coverslips with stained cells were mounted in an Attofluor cell chamber (ThermoFisher Scientific A7816) with freshly prepared STORM imaging buffer (see composition below) and the chamber covered with a glass coverslip to minimize contact with oxygen. To visualize focal adhesions in order to define the types of stress fibers measured, AF488-phalloidin and phospho-FAK-co-stained cells were imaged before each STORM acquisition on an optical setup employing a confocal spinning-disk unit (CSU-X1-M1 from Yokogawa) connected to the side-port of an inverted microscope (Eclipse Ti-U from Nikon Instruments), using a Nikon Plan Apo ×100/1.45 NA oil immersion objective lens, 488- and 641-nm laser lines (Coherent) and an iXon Ultra 888 EMCCD camera (1024 × 1024 pixels, 13 × 13 μm pixel size, Andor, Oxford Instruments). z-stacks were acquired with a Δz interval of 0.5 μm.

### B16-F1 cells

24 mm-diameter high-precision (170 μm ± 5 μm) glass coverslips (Marienfeld, 0117640) were sonicated in 70% ethanol for 5 min and air-dried before coating with mouse laminin (SIGMA L2020) for 1 h at RT and at a final laminin concentration of 25 μg/mL in coating buffer (50 mM Tris-HCl pH 8, 150 mM NaCl). B16-F1 cells were seeded onto laminin-coated coverslips and allowed to spread overnight. To stimulate lamellipodia formation, cells were treated with aluminum fluoride for 15 min by adding AlCl_3_ and NaF to final concentrations of 50 μM and 30 mM, respectively, in prewarmed, full growth medium. Cells were fixed for 20 min with a mixture of prewarmed (37 °C) 0.25% glutaraldehyde (Electron Microscopy Sciences 16220) and 4% formaldehyde (Electron Microscopy Sciences 15714) in cytoskeleton buffer, and treated with fresh sodium borohydride (1 mg/mL) in PBS for 2 × 5 min to reduce background fluorescence. Cells were washed in PBS for 3 × 5 min before an overnight incubation with 0.5 μM AF488-phalloidin in 0.1% saponin/10% BSA/PBS at 4 °C in a humidified chamber. For 4polar-STORM measurements, coverslips were mounted as for U2OS cells.

### Blebbistatin treatment

Blebbistatin from Sigma (B0560) was prepared at 10 mM in DMSO. U2OS cells were seeded onto fibronectin-coated medium-size H-shaped patterns from CYTOO and allowed to spread for 5 h, as detailed above. Cells were incubated for 15 min with 50 μM blebbistatin (i.e. in medium also containing 0.5% DMSO due to the blebbistatin stock dilution), or with medium containing 0.5% DMSO (control cells). Cells were fixed with 4% formaldehyde in cytoskeleton buffer for 15 min, and washed in PBS for 2 × 5 min before an overnight incubation with 0.5 μM AF488-phalloidin in 0.1% saponin/10% BSA/PBS at 4 °C in a humidified chamber. Cells were mounted for 4polar-STORM measurements as detailed above.

### Reconstitution of single actin filaments for 4polar-STORM

Lyophilized rabbit skeletal muscle G-actin (Cytoskeleton, Inc. AKL99) was resuspended to 5 mg/mL (119 μM) in G-buffer (5 mM Tris-HCl pH 8, 0.2 mM Na_2_ATP, 0.1 mM CaCl_2_, 1 mM DTT), aliquots snap-frozen in liquid nitrogen and stored at −80 °C. Frozen aliquots were thawed and centrifuged for 30 min at 120,000 g in a benchtop Beckman air-driven ultracentrifuge (Beckman Coulter Airfuge, 340401) to clear the solution from aggregates. Clarified G-actin was kept at 4 °C and used within 3–4 weeks. For reconstitution experiments, G-actin was polymerized at 5 μM final concentration in actin polymerization buffer (5 mM Tris-HCl pH 8, 50 mM KCl, 1 mM MgCl_2_, 0.2 mM Na_2_ATP, 1 mM DTT) in the presence of 5 μM AF488-phalloidin for at least 2 h at RT. Flow cells for measurements on reconstituted actin filaments were prepared as follows. Microscope glass slides and coverslips were cleaned for 15 min in base-piranha solution, rinsed twice, 5 min each, with Milli-Q water in a bath sonicator, and stored in ethanol up to one month. To assemble flow cells, slides and coverslips were dried with synthetic air, and ~10 μL channels were assembled by sandwiching ~2-mm wide and ~2.5-cm-long strips of Parafilm between a cleaned glass slide and coverslip and melting on a hot plate at 120 °C. The chambers were incubated for 45 min with 1 M KOH, rinsed with actin polymerization buffer, incubated for another 15 min with 1 mg/mL poly-L-lysine (PLL; Sigma P8920), and rinsed with actin polymerization buffer. Reconstituted AF488-phalloidin-labeled actin filaments were diluted to 0.1–0.2 μM, loaded into the PLL-coated flow channels and left for 15 min to immobilize actin filaments. Actin polymerization buffer was then exchanged with STORM imaging buffer (see composition below), and flow channels sealed with VALAP (1:1:1 vasoline:lanoline:paraffin). The typical experimental conditions were TIRF illumination, laser power 150 mW, camera gain 300 and 200-ms integration time. A stack of 5000 images was used for 4polar-STORM imaging. The materials and chemicals for glass cleaning were as follows. Glass slides (26 × 76 mm) from Thermo Scientific (AA00000102E01FST20). Glass coverslips (24 × 60 mm) from Thermo Scientific (BB02400600A113FST0). Ammonium hydroxide solution from SIGMA (221228). Hydrogen peroxide solution from SIGMA (95299).

### STORM imaging buffer preparation

The final composition of the buffer for 4polar-STORM measurements was 100 mM Tris-HCl pH 8, 10% w/v glucose, 5 U/mL pyranose oxidase (POD), 400 U/mL catalase, 50 mM β-mercaptoethylamine (β-MEA), 1 mM ascorbic acid, 1 mM methyl viologen, and 2 mM cyclooctatetraene (COT). D-(+)-glucose was from Fisher Chemical (G/0500/60). POD was from Sigma (P4234-250UN), bovine liver catalase from Calbiochem/Merck Millipore (219001-5MU), β-MEA from Sigma (30070), L-ascorbic acid from Sigma (A7506), methyl viologen from Sigma (856177), and COT from Sigma (138924). Glucose was stored as a 40% w/v solution at 4 °C. POD was dissolved in GOD buffer (24 mM PIPES pH 6.8, 4 mM MgCl_2_, 2 mM EGTA) to yield 400 U/mL, and an equal volume of glycerol was added to yield a final 200 U/mL in 1:1 glycerol:GOD buffer; aliquots were stored at −20 °C. Catalase was dissolved in GOD buffer to yield 10 mg/mL, and an equal volume of glycerol was added to yield a final 5 mg/mL (230 U/μL) of catalase in 1:1 glycerol:GOD buffer; aliquots were stored at −20 °C. β-MEA was stored as ~77 mg powder aliquots at −20 °C; right before use, an aliquot was dissolved with the appropriate amount of 360 mM HCl to yield a 1 M β-MEA solution. Ascorbic acid was always prepared right before use at 100 mM in water. Methyl viologen was stored as a 500 mM solution in water at 4 °C. COT was prepared at 200 mM in DMSO and aliquots stored at −20 °C. After mixing all components to yield the final buffer composition, the buffer was clarified by centrifugation for 2 min at 16,100 g, and the supernatant kept on ice for 15 min before use. Freshly prepared STORM buffer was typically used within a day.

### Monte Carlo simulations

The single molecules’ images are generated from intensities that follow the model of Supplementary Note 1. The starting parameters are the detection NA = 1.2, total intensity ${I}_{T}={I}_{0}+{I}_{45}+{I}_{90}+{I}_{135}$, (from 1000 to 10 000 photons), background pixel value (from 0 to 60 photons/pixel) and the angular (δ,ρ,η) parameters. This defines a set of intensities $({I}_{0},{I}_{45},{I}_{90},{I}_{135})$ which are used for the generation of Gaussian point spread functions (PSF) to which noise is added using the model of Supplementary Fig. S4. For each molecule centered at coordinates $\left({i}_{n},{j}_{n}\right)$, a 2D PSF shape is calculated as ${I}_{{0,45,90,135}}\left(i,j\right)={I}_{{0,45,90,135}}\left(\delta ,\rho ,\eta \right).{G}_{{ij}}\left({i}_{n},{j}_{n},r\right)+N({I}_{{0,45,90,135}})+b$ with *b* the background/pixel value, and ${G}_{{ij}}\left({i}_{n},{j}_{n},r \right)=1/ \big(\sqrt{\pi }r\big).{{\exp }}\big(\!-\!{\big(\left(i-{i}_{n}\right)}^{2}+{\big(\left(i-{i}_{n}\right)}^{2}\big)/{2r}^{2}\big)$ the Gaussian 2D shape of radius *r*. *N*(*I*) is the noise added to the intensity *I*, which follows the experimental noise (see Supplementary Fig. S4). The detection parameters are identical to experimental ones (see Supplementary Note 2). Different settings can be used in this simulation such as total intensity, background level, PSF radius, calibration factors (see Supplementary Note 2), possibly PSF anisotropy along two orthogonal directions (in this case the Gaussian function is set with two different sizes *r*_*x*_ and *r*_*y*_ along *x* and *y*, respectively). In total, 500 to 1000 molecules with identical initial settings are typically generated, distributed in 5 to 10 molecules per images to simulate a 4polar-STORM detection process. The 4polar-STORM detection code is run on a set of 100 to 1000 images, following the procedure of Supplementary Note 3.

### Reporting summary

Further information on research design is available in the Nature Research Reporting Summary linked to this article.

## Supplementary information

Supplementary Information

Reporting Summary

## Data Availability

The 4polar-STORM raw image stacks are available on request from the corresponding author, for their large size reason. A subset of data (2Go) as well as processed localization data in polarized channels are available at 10.6084/m9.figshare.17167004. Processed data of example ROIs generated in this study are available for download at https://gitlab.fresnel.fr/mosaic/4polarSTORM (TestData folder) with explanations of use in the README.md file. All processed orientation/detection parameters from single-molecule data generated in this study are provided in the Source Data file and are available at 10.6084/m9.figshare.17167001. Source data are provided with this paper.

## Source data

Source Data

## Code availability

The code used to analyze the data is available in the Gitlab webpage https://gitlab.fresnel.fr/mosaic/4polarSTORM, which contains a manual and installation instructions.
