# Supplementary material for: 4polar-STORM polarized super-resolution imaging of actin filament organization in cells
Source: Nat Commun. 2022 Jan 13;13:301. doi: 10.1038/s41467-022-27966-w (PMC8758668; doi:10.1038/s41467-022-27966-w)
Supplement: Supplementary file 1 — Supplementary Information [file 41467_2022_27966_MOESM1_ESM.pdf]

4polar-STORM polarized super-resolution imaging of actin filament organization in cells

Caio Vaz Rimoli[1,&], Cesar Augusto Valades Cruz[2,3,&], Valentina Curcio[1], Manos Mavrakis[1,*], Sophie Brasselet[1,*]

1 Aix Marseille Univ, CNRS, Centrale Marseille, Institut Fresnel, F-13013 Marseille, France

2 Institut Curie, PSL Research University, UMR144 CNRS, Space-Time imaging of organelles and Endomembranes Dynamics Team, F-75005, Paris, France

3 Inria Centre Rennes-Bretagne Atlantique, SERPICO Project Team, F-35042, Rennes, France

[&] these authors contributed equally to the work

* corresponding authors contact : sophie.brasselet@fresnel.fr, manos.mavrakis@univ-amu.fr

Content

Supplementary Notes

Supplementary Note 1. Model and retrieval of orientation parameters
Supplementary Note 2. Calibration factors in 4polar-STORM
Supplementary Note 3. Data processing algorithm of the 4polar-STORM method

Supplementary Figures

Figure S1. Notations for dipole radiation coordinates.
Figure S2. Principle of the retrieval of δ from 4polar-STORM polarization factors.
Figure S3. Principle of the retrieval of ρ from 4polar-STORM polarization factors.
Figure S4. Camera noise estimation and Monte Carlo simulation parameters.
Figure S5. Simulations of the effect of variations of the G factors on the accuracy of (δ,ρ).
Figure S6. Fit of typical point spread functions in simulated and experimental images.
Figure S7. Effect of the detection of zero-intensities molecules on the (δ,ρ) retrieval.
Figure S8. 4polar-STORM algorithm flowchart.
Figure S9. 4polar-STORM stick representations of (δ,ρ).
Figure S10. Monte Carlo simulations of the effect of the total intensity and presence of background on the accuracy of (δ,ρ) at η=90°.
Figure S11. Monte Carlo simulations of the effect of the intensity and background on the accuracy and precision on (δ,ρ) for δ=100°, ρ=30°, at variable η.
Figure S12. Monte Carlo simulations of the effect of the total intensity and background on the accuracy of (δ,ρ) at variable δ for η = 60°.
Figure S13. Monte Carlo simulation of the effect of the total intensity and background on the localization precision and retrieved radius of single molecule PSFs.
Figure S14. Effect of the proximity of single molecules on the orientation retrieval efficiency.

Figure S15. Effect of the deformation of single molecule PSFs on the orientation retrieval efficiency.
Figure S16. Statistics on detection parameters in 4polar-STORM imaging of F-actin in stress fibers in cells.
Figure S17. 4polar-STORM δ images of F-actin in fixed U2OS cells labelled with AF488-phalloidin.
Figure S18. Comparison of δ and ρ values in single filaments and stress fibers in fixed cells.
Figure S19. Correlation between the different detection parameters and the δ and ρ parameters.
Figure S20. Retrieval bias on $\sigma_{\Delta\rho}$.
Figure S21. Effect of the measured PSF radius on the δ and ρ statistics.
Figure S22. Effect of the intensity, σ_{loc} and *r* on the δ and ρ statistics.
Figure S23. 4polar-STORM imaging of F-actin in cells, selecting in-plane actin filament populations.
Figure S24. Effect of the detection-parameter filtering on the $\Delta\rho$ statistics for the population $\delta < 110°$.
Figure S25. 4polar-STORM imaging of actin filament organization in lamellipodia.

Supplementary Notes

Supplementary Note 1. Model and retrieval of orientation parameters

A single molecule orientation is determined by its absorption dipole $\vec{\mu}_a$ and emission dipole $\vec{\mu}_e$, which lie along transition dipole moment directions for the respective absorption and emission transitions. Dipole orientations (θ, φ) (Fig. S1) are defined in the frame of the distribution angles that the molecule explores during the integration time of the image (typically tens to hundreds ms, which is much longer than the rotational time of the molecules). We consider a molecule wobbling within a distribution $f(\theta, \varphi)$ during the integration time of the detector. $f(\theta, \varphi)$ is defined here as a cone function, of value 1 for $(0 \leq \theta \leq \delta/2)$ and 0 elsewhere (a Gaussian function would lead to very similar results). ρ is the orientation of the projection of the cone in the sample plane (x, y), and η is the out-of-plane orientation of the cone, relative to z (see Fig. S1).
We suppose that the molecular absorption and emission dipoles lie along the same direction (θ, φ). This assumption is valid in the case where the rotational time is slower than the fluorescence lifetime (still being faster than the integration time of each image). The absorption and emission dipoles, denoted $\vec{\mu}_a(\theta, \varphi)$ and $\vec{\mu}_e(\theta, \varphi)$, are expressed in the macroscopic frame (x, y, z) of the sample as:

$$\vec{\mu}_{a,e}(\theta, \varphi, \rho, \eta) = \mu_{a,e}\, R \cdot \begin{bmatrix} \sin\theta\cos\varphi \\ \sin\theta\sin\varphi \\ \cos\theta \end{bmatrix}$$

$$R = \begin{bmatrix} \sin\eta\cos\rho & -\sin\rho & \cos\eta\cos\rho \\ \sin\eta\sin\rho & \cos\rho & \cos\eta\sin\rho \\ -\cos\eta & 0 & \sin\eta \end{bmatrix}$$

Eq. S1

Where $\mu_{a,e}$ is the dipole amplitude and R is the rotation matrix from the molecular to the macroscopic frame.

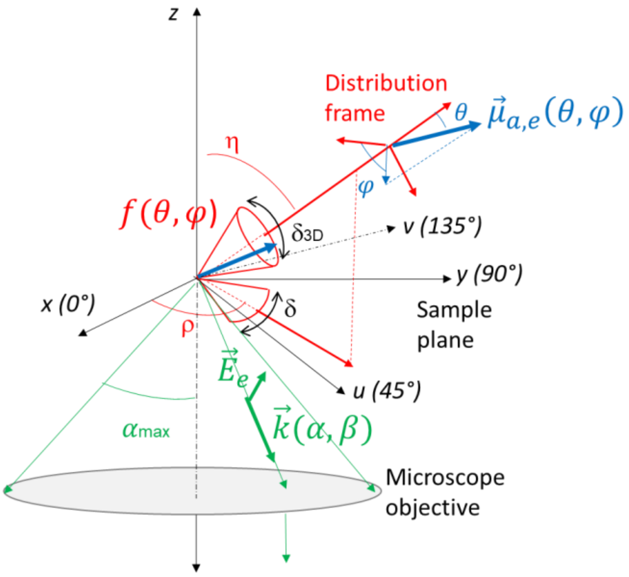

Figure S1. Notations for dipole radiation coordinates.

The fluorescence signal is the result of an excitation step, whose efficiency is quantified by the absorption probability, and an emission step, in which the emission dipole radiates. A complete derivation of the fluorescence signal from wobbling dipoles can be found for instance in[1]. Here we provide the expressions used in this work to extract the orientation parameters from single dipoles, from polarized intensities measured in the 4polar-STORM method.

Excitation. The absorption probability is proportional to[2]:

$$P_{abs}(\theta,\varphi,\rho,\eta) \propto \left|\vec{\mu}_a(\theta,\varphi,\rho,\eta)\cdot\vec{E}\right|^2 \quad \text{Eq. S2}$$

with $\vec{E}$ the excitation field.

In the case of normal incidence circular polarization, $\vec{E} = E_0(1,1,0)$ with E_0 the field amplitude.

In the case of total internal reflection (TIRF) illumination, $\vec{E} = (E_x, E_y, E_z)$ takes a more complex form that can be found in[3], which involves a contribution along z. In the present work, the incident polarization in TIRF is set such as the in-plane (E_x, E_y) contributions are balanced, in order to minimize any in-plane photoselection.

Emission. The emission field radiated from the emission dipole $\vec{\mu}_e$ along the propagation direction $\vec{k}$ writes, in free space:

$$\vec{E}_e(\theta,\varphi,\rho,\eta,\vec{k}) \propto (\vec{k}\times\vec{\mu}_e(\theta,\varphi,\rho,\eta))\times\vec{k} \quad \text{Eq. S3}$$

In reality the dipole is placed within a medium of refractive index supposedly close to water, above an interface with glass. To account for these interfaces, expressions derived in[4] are used.

Fluorescence intensity. The fluorescence intensity detected along a polarization direction $\vec{\varepsilon}$ and a propagation vector $\vec{k}$ (see Fig. S1) is deduced from the absorption and emission probabilities product[2,5,6]:

$$I_\varepsilon(\vec{k}) \propto \int_0^{2\pi} d\varphi \int_0^{\delta/2} d\theta\ \sin\theta\, P_{abs}(\theta,\varphi)\left|\vec{E}_e(\theta,\varphi,\rho,\eta,\vec{k})\cdot\vec{\varepsilon}\right|^2 \quad \text{Eq. S4}$$

Where $\propto$ encompasses all collection/excitation efficiency factors that do not affect the present analysis.

To account for the detection numerical aperture (NA), it is necessary to integrate all vector fields directions $\vec{k}(\alpha,\beta)$ over the collected NA, with $(0 \le \alpha \le \alpha_{max}, 0 \le \beta \le 2\pi)$. The total detected intensity writes therefore as:

$$I_\varepsilon = \int_0^{2\pi} d\beta \int_0^{\alpha_{max}} d\alpha\ \sin\alpha\, I_\varepsilon(\vec{k}(\alpha,\beta)) \quad \text{Eq. S5}$$

Which means that at high NA integration, the detection is affected by a mixture of polarization contributions.

In this work, the detection is performed along the polarization directions (0°, 90°, 45°, 135°), e.g. $\vec{\varepsilon} = \vec{x}, \vec{y}, \vec{u}, \vec{v}$ with $\vec{u} = (\vec{x} + \vec{y})/\sqrt{2}$ and $\vec{v} = (\vec{y} - \vec{x})/\sqrt{2}$. We denote $(I_0, I_{90}, I_{45}, I_{135})$ the measured intensities along these polarization directions. Equations S4, S5 can be used to express the four measured intensities in a matrix form[1] :

$$I_\varepsilon = \int_0^{2\pi} d\beta \int_0^{\alpha_{max}} d\alpha \, \sin\alpha \left[E_\varepsilon^{\mu_x}(\vec{k})^* \quad E_\varepsilon^{\mu_y}(\vec{k})^* \quad E_\varepsilon^{\mu_z}(\vec{k})^*\right] \langle P_{abs} \cdot \vec{\mu} \cdot \vec{\mu}^T \rangle \begin{bmatrix} E_\varepsilon^{\mu_x}(\vec{k}) \\ E_\varepsilon^{\mu_y}(\vec{k}) \\ E_\varepsilon^{\mu_z}(\vec{k}) \end{bmatrix}$$

Eq. S6

$\vec{\mu}_e$ is simplified into $\vec{\mu}$ in what follows. The average $\langle \quad \rangle$ is performed over all molecular orientations, and $E_\varepsilon^{\mu_j}(\vec{k})$ is the projection along $\vec{\varepsilon} = \vec{x}, \vec{y}, \vec{u}, \vec{v}$ of the field radiated by the dipole component μ_j, whose full expression can be found in[1] and [4].

So far we have considered the case where the absorption and emission dipoles have an identical orientation. Two situations can differ from this situation: (i) the molecular structure in its ground and excited states is structurally different, which causes an intrinsically different angle considering transitions involved in the absorption and emission events. This angle is fixed and can be accounted for. In the case of the Alexa fluorophores studied in this work, this angle is likely to be very small and will thus be ignored. (ii) The rotation time of the molecule is much faster than its fluorescence lifetime (which is of the order of several ns). In this case, the absorption and emission orientations are decorrelated in time, therefore the absorption probability averages-out in Eq. S6, which means that $\langle P_{abs} \cdot \vec{\mu} \cdot \vec{\mu}^T \rangle$ is changed into $\langle P_{abs} \rangle \langle \vec{\mu} \cdot \vec{\mu}^T \rangle$ into this equation. With such a fast rotational diffusion behaviour, the emission probability is homogeneous within the diffusion cone, while in the slow rotational diffusion case, the excitation field induces an asymmetry of this emission probability from[7].

In what follows we derive the relation between the dipolar quantities in Eq. S6, and the orientation parameters of single molecules. Both $\langle P_{abs} \cdot \vec{\mu} \cdot \vec{\mu}^T \rangle$ and $\langle P_{abs} \rangle \langle \vec{\mu} \cdot \vec{\mu}^T \rangle$ (in the case of a fluorophore rotating slower versus faster than the fluorescence lifetime scale) can be expressed in a matrix form:

$$M = \begin{bmatrix} M_{xx} & M_{xy} & M_{xz} \\ M_{yx} & M_{yy} & M_{yz} \\ M_{zx} & M_{zy} & M_{zz} \end{bmatrix}$$ Eq. S7

where $M_{jk} = \langle P_{abs} \cdot \mu_j \cdot \mu_k^* \rangle$, with $i = 0,90,45,135$ and $j, k = x, y, z$, for molecules rotating slower than the ns-time scale and $M_{jk} = \langle P_{abs} \rangle \langle \mu_j \cdot \mu_k^* \rangle$ for molecules rotating faster than the ns-time scale.

Following Eqs. S6 and S7, the 4polar channel intensities can be related to the dipoles' dependent M matrix coefficients, in a matrix form:

$$\begin{pmatrix} I_0 \\ I_{90} \\ I_{45} \\ I_{135} \end{pmatrix} = K \cdot \begin{pmatrix} M_{xx} \\ M_{yy} \\ M_{zz} \\ M_{xy} \\ M_{xz} \\ M_{yz} \end{pmatrix} = \begin{pmatrix} K_{0(xx)} & \cdots & K_{0(yz)} \\ \vdots & \ddots & \vdots \\ K_{135(xx)} & \cdots & K_{135(yz)} \end{pmatrix} \cdot \begin{pmatrix} M_{xx} \\ M_{yy} \\ M_{zz} \\ M_{xy} \\ M_{xz} \\ M_{yz} \end{pmatrix}$$

with

$$K_{i(jk)} = \int_0^{2\pi} d\alpha \int_0^{\alpha_{max}} d\beta \ \sin\beta \ 2 \ Re\left(E_i^{\mu_j}(\alpha,\beta)^* E_i^{\mu_k}(\alpha,\beta)\right) \qquad \text{Eq. S8}$$

α_{max} is the limit angle of integration with a maximum value reached at the NA of the objective: $\alpha_{max} = \text{asin}(NA/n)$ with n the immersion medium refractive index.

In Eq. S8, the K matrix contains terms only related to the propagation setup while the M elements relate to the orientation parameters of the molecular dipoles. The goal of the 4polar-STORM experiment is to retrieve orientation information from the measured M elements. An analysis of the K matrix is first performed, to deduce which M elements can be retrieved in 4polar-STORM.

The calculation of the $K_{i(jk)}$ elements using Eq. S8 ultimately gives rise to the following form of K:

$$K = \begin{bmatrix} a & b & c & 0 & 0 & 0 \\ b & a & c & 0 & 0 & 0 \\ d & d & c & e & 0 & 0 \\ d & d & c & -e & 0 & 0 \end{bmatrix} \qquad \text{Eq. S9}$$

The coefficients (a, b, c, d, e) depend on the numerical aperture used in the detection path. These coefficients are not totally independent since by construction of this matrix, $(a + b) = 2d$ and $(a - b) = e$. Importantly, for NAs above the critical angle of the sample interface (for instance NA 1.45), these coefficients will also depend on the distance of the dipole from the interface, making the experiment sensitive to another parameter that is uncontrolled.

The form of K is consistent with a 2D projection measurement of the dipoles angular information : it does not allow to retrieve M_{xz}, M_{yz} (the related matrix elements being 0), nor M_{zz} which is an additive term to all measured intensities. cM_{zz} appears here as a leakage factor from the 3D dipole's orientation, which affects similarly all measured intensities, with c increasing with the numerical aperture NA. From the form of the matrix, it therefore appears that the only independent retrievable parameters from the 4 intensity measurements, through a normalization by the total intensity, are: (M_{xx}, M_{yy}, M_{xy}).

An analysis of the form of the K matrix shows that simple combinations of (M_{xx}, M_{yy}, M_{xy}) can be retrieved using two independent normalized factors:

$$P_0 = \frac{I_0 - I_{90}}{I_0 + I_{90}} = \frac{(a-b)(M_{xx} - M_{yy})}{I_T/2}$$

$$P_{45} = \frac{I_{45} - I_{135}}{I_{45} + I_{135}} = \frac{(a-b) \cdot 2M_{xy}}{I_T/2}$$

Eq. S10

Here we used $e = (a - b)$, and $I_T = I_0 + I_{90} + I_{45} + I_{135}$ the total intensity.

We show now that the forms measurable from Eq. S10 are sufficient to retrieve 2D molecular orientational parameters from a 4polar-STORM measurement.

In the case of a molecules rotating slower than their fluorescence lifetime, which is probable for molecules attached to a protein in a viscous cell environment, the full expression of the terms (M_{xx}, M_{yy}, M_{xy}) is:

$$M_{jk}(\rho, \delta, \eta) = \frac{1}{N} \int_0^{2\pi} d\varphi \int_0^{\delta/2} d\theta \ \sin\theta \, P_{abs}(\theta, \varphi, \rho, \eta) \, \mu_j(\theta, \varphi, \rho, \eta) \cdot \mu_k^*(\theta, \varphi, \rho, \eta)$$

$$P_{abs}(\theta, \varphi) = \left| \overrightarrow{E_{exc}} \cdot \vec{\mu}(\theta, \varphi, \rho, \eta) \right|$$

Eq. S11

with (j, k)= (x, y, z) and N the normalization factor $N = \int_0^{2\pi} d\varphi \int_0^{\delta/2} d\theta \ \sin\theta$.

The dipole's $\mu_{j,k}$ components are expressed in Eq. S1. Accounting for the excitation field polarization (here, in TIRF condition), it can be shown that M is written :

$$M = R \begin{bmatrix} \lambda_{11}(\delta) & \lambda_{12}(\delta) & 0 \\ \lambda_{12}(\delta) & \lambda_{22}(\delta) & \lambda_{23}(\delta) \\ 0 & \lambda_{23}(\delta) & \lambda_{33}(\delta) \end{bmatrix} R^T$$ Eq. S12

With the rotation R matrix expressed in Eq. S1. In the present conditions, it has been found that $(\lambda_{12}(\delta), \lambda_{23}(\delta)) \ll (\lambda_{11}(\delta), \lambda_{22}(\delta), \lambda_{33}(\delta))$, therefore the off-diagonal terms will be neglected. Accounting for a TIRF excitation at a water/glass interface (refractive indexes 1.33 and 1.5, respectively), $\overrightarrow{E_{exc}}$ is well determined and the $\lambda_{11}(\delta), \lambda_{22}(\delta)$, $\lambda_{33}(\delta)$ coefficients can therefore be calculated as numerical forms that depend only on δ.

In the case of a molecules rotating faster than their fluorescence lifetime, the form of M is purely diagonal as demonstrated in[1]. The term $\langle P_{abs} \rangle$ is a common factor to all coefficients and the diagonal terms of the function $\langle \vec{\mu} \cdot \vec{\mu}^T \rangle$ in M take simple forms which depend only on δ [1]:

$$\lambda_{11}(\delta) = \lambda_{22}(\delta) = \frac{\left(1 - \cos\left(\frac{\delta}{2}\right)\right)\left(2 + \cos\left(\frac{\delta}{2}\right)\right)}{6}$$

$$\lambda_{33}(\delta) = \frac{\left(\cos^3\left(\frac{\delta}{2}\right) - 1\right)}{3\left(\cos\left(\frac{\delta}{2}\right) - 1\right)}$$

Eq. S13

Finally in both cases (fast and slow with respect to the fluorescence lifetime), the expression of the measurable M components is therefore :

$$M_{xx} = \lambda_{11}(\delta)\ \cos^2\eta\cos^2\rho + \lambda_{22}(\delta)\sin^2\rho + \lambda_{33}(\delta)\ \sin^2\eta\cos^2\rho$$
$$M_{yy} = \lambda_{11}(\delta)\ \cos^2\eta\sin^2\rho + \lambda_{22}(\delta)\cos^2\rho + \lambda_{33}(\delta)\ \sin^2\eta\sin^2\rho$$
$$M_{xy} = \sin\rho\cos\rho\,[\lambda_{11}(\delta)\ \cos^2\eta - \lambda_{22}(\delta) + \lambda_{33}(\delta)\ \sin^2\eta]$$

Eq. S14

Eq. S14 evidences two independent retrievable parameters:

$$M_{xx} - M_{yy} = \cos 2\rho\ \cdot\ g(\delta,\eta)$$

$$M_{xy} = \sin 2\rho\ \cdot\ g(\delta,\eta)$$

Eq. S15

With $g(\delta,\eta) = \lambda_{11}(\delta)\ \cos^2\eta + \lambda_{33}(\delta)\ \sin^2\eta - \lambda_{22}(\delta)$.

Note that η cannot be retrieved independently from a 4polar-STORM measurement, which is by nature a 2D projection. Supposing the distribution in the sample plane (e.g. $\eta = 90°$), $g(\delta,\eta)$ becomes a function of δ only :

$$g(\delta) = \lambda_{33}(\delta) - \lambda_{22}(\delta) \qquad \text{Eq. S16}$$

To retrieve independently ρ from δ, it is therefore necessary to use combinations of $\left(M_{xx} - M_{yy}\right)$ and M_{xy} :

$$\tan 2\rho = \frac{M_{xy}}{M_{xx} - M_{yy}}$$
$$g(\delta)^2 = \left(M_{xx} - M_{yy}\right)^2 + M_{xy}^2 \qquad \text{Eq. S17}$$

As seen above, the two independent key elements $\left(M_{xx} - M_{yy}\right)$ and M_{xy} are extractable from the measured 4polar-STORM intensities, through the polarization factors P_0 and P_{45} defined in Eq. S10. It is therefore possible to retrieve independently the parameters (ρ,δ) from a ratiometric analysis of the 4polar-STORM data.

The data analysis of 4polar-STORM can be based on the calculation or pre-calibration of the K matrix, followed by a measurement of the polarization factors P_0 and P_{45} defined in Eq. S10 to extract $\left(M_{xx} - M_{yy}\right)$ and M_{xy}, and finally a numerical resolution of Eq. S17 to extract (ρ,δ). We propose in what follows a simplification of the analysis based on the intrinsic 2D projection nature of the 4polar-STORM experiment. Assuming a paraxial approximation, the measured intensities polarized along the $\vec{\varepsilon}$ direction reduce to:

$$I_{\varepsilon}(\theta,\varphi,\rho,\eta) \propto |\vec{\mu}(\theta,\varphi,\rho,\eta)\cdot\vec{\varepsilon}|^2 \qquad \text{Eq. S18}$$

Simplifying the cone distribution function into a 2D-flat cone ($\theta = \frac{\pi}{2}$), the integration over the distribution function simplifies the detected intensities into :

$$I_0 = \frac{I_T}{2\delta}\int_{\rho-\delta/2}^{\rho+\delta/2}\cos^2\varphi d\varphi = \frac{I_T}{2}(\cos 2\rho \operatorname{sinc}\delta + 1)$$
$$I_{90} = \frac{I_T}{2\delta}\int_{\rho-\delta/2}^{\rho+\delta/2}\sin^2\varphi d\varphi = \frac{I_T}{2}(1 - \cos 2\rho \operatorname{sinc}\delta)$$
$$I_{45} = \frac{I_T}{2\delta}\int_{\rho-\delta/2}^{\rho+\delta/2}\cos^2\left(\varphi - \frac{\pi}{4}\right)d\varphi = \frac{I_T}{2}(\sin 2\rho \operatorname{sinc}\delta + 1)$$
$$I_{135} = \frac{I_T}{2\delta}\int_{\rho-\delta/2}^{\rho+\delta/2}\sin^2\left(\varphi - \frac{\pi}{4}\right)d\varphi = \frac{I_T}{2}(1 - \sin 2\rho \operatorname{sinc}\delta) \qquad \text{Eq. S19}$$

With $\operatorname{sinc}\delta = \frac{\sin\delta}{\delta}$ and I_T the total intensity.
Under these conditions,

$$P_0(\rho,\delta) = \frac{I_0 - I_{90}}{I_0 + I_{90}} = \cos 2\rho \operatorname{sinc}\delta$$

$$P_{45}(\rho,\delta) = \frac{I_{45}-I_{135}}{I_{45}+I_{135}} = \sin 2\rho \operatorname{sinc}\delta \qquad \text{Eq. S20}$$

In this situation, the orientation parameters can be easily deduced from the polarization factors P_0 and P_{45} with

$$\rho = \frac{1}{2}\operatorname{atan}\left(\frac{P_{45}}{P_0}\right)$$
$$\operatorname{sinc}\delta = \sqrt{P_0^2 + P_{45}^2} \qquad \text{Eq. S21}$$

These expressions will be used to analyse the 4polar-STORM results, and their validity in real situations (high detection NA, TIRF excitation, tilted wobbling distribution) is discussed below. Importantly, Eq. S21 confirms that the 4polar-STORM method permits to estimate ρ and δ independently, which is not the case of polarization schemes that use only two polarization projections[8]. Note that the forms obtained in Eq. S20 are reminiscent of the expression of the quantities M_{xx}, M_{yy} and M_{xy} ignoring the 3D expansion of the cone distribution (e.g. this distribution becomes a 2D-flat cone) and the z contribution of the molecular excitation. In this situation, Eq. S11 becomes:

$$M_{xx} = \frac{\mu^2}{2\delta}\int_{\rho-\delta/2}^{\rho+\delta/2}\cos^2\varphi d\varphi = \frac{\mu^2}{2}(\cos 2\rho \operatorname{sinc}\delta + 1)$$
$$M_{yy} = \frac{\mu^2}{2\delta}\int_{\rho-\delta/2}^{\rho+\delta/2}\sin^2\varphi d\varphi = \frac{\mu^2}{2}(1 - \cos 2\rho \operatorname{sinc}\delta)$$
$$M_{xy} = \frac{\mu^2}{2\delta}\int_{\rho-\delta/2}^{\rho+\delta/2}\sin\varphi.\cos\varphi\, d\varphi = \mu^2\,(\sin 2\rho \operatorname{sinc}\delta) \qquad \text{Eq. S22}$$

It is visible that the P_0 and P_{45} factors are still related to $\left(M_{xx} - M_{yy}\right)$ and M_{xy}, as expressed in Eq. S10 from a fully rigorous propagation derivation.

Equation S21 is valid for wobbling molecules lying in a flat cone in the sample plane and in the paraxial approximation. When molecular distributions resemble a full cone, possibly tilted off-plane ($\eta < 90°$), radiating through a high NA imaging microscope, the retrieval of the (ρ, δ) parameters follows Eqs. S10,S15. To test the validity of the simplified model on a real situation assuming a known detection NA and excitation field, we simulated the dependence of the P_0 and P_{45} polarization factors in the two models. We denote δ_{3D} the ground-truth wobbling angle in 3D, and δ its value measured from a 4polar-STORM measurement. Figure S2 shows the δ_{3D}-dependence of the polarization factor $P = \sqrt{P_0^2 + P_{45}^2}$ for the approximated 2D model used for the parameter retrieval ($P(\delta) = \mathrm{sinc}\,\delta$, Eq. S21) compared to a complete model for NA = 1.2 and NA = 1.45 (Eq. S10). For wobbling distributions lying in the sample plane and at the coverslip interface, the dependence shows that with the approximate model, δ is estimated at NA = 1.2 with a slight bias only for low (< 40°) and high (> 160°) δ values (Fig. S2b). At NA = 1.45, the retrieval of δ is highly biased for all δ values. These dependences show that the sensitivity of P to δ is higher at low NA. For a fully reliable determination of δ, exact non-approximate models could be used, however for NA 1.45, $P(\delta)$ is highly sensitive to the distance z from the dipole emitter to the coverslip interface (Fig. S2b), which would require in addition the knowledge of z, which is not feasible in practice. In contrast, the low NA situation (e.g. below the critical angle) is independent on z. $P(\delta)$ at NA = 1.2 is also seen to be less affected by the off-plane tilt angle η than at NA = 1.45 (Fig. S2c). At NA = 1.2, $P(\delta)$ varies up to ~ 25% at $\eta = 60°$ (55% at $\eta = 45°$), while at NA = 1.45, it varies up to 40% and 65% at $\eta = 60°$ and 45° respectively.

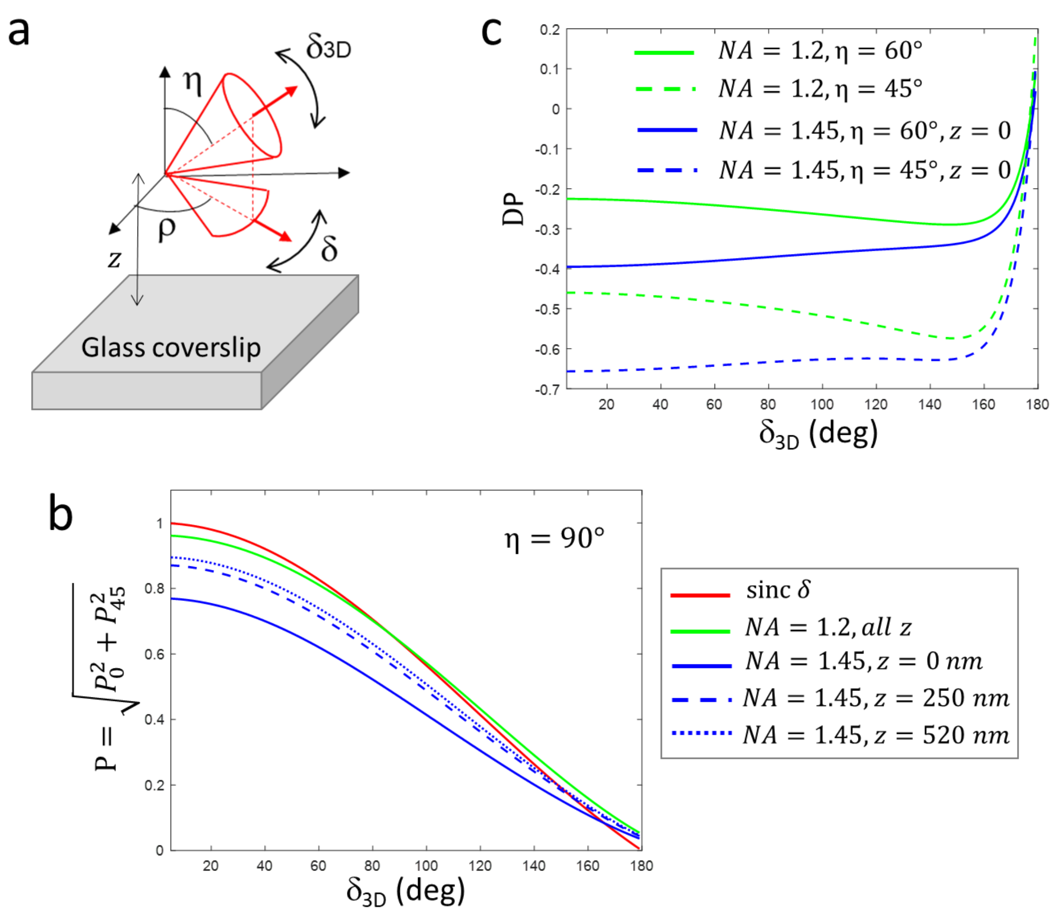

Figure S2. Principle of the retrieval of δ from 4polar-STORM polarization factors. (a) Schematic representation of a single molecule oriented in 3D by the mean orientation angles (ρ,η) and a wobbling cone angle δ_{3D}. The measured wobbling in 2D is δ. z is the distance from the dipole emitter to the water-glass interface. The wavelength used for the model is 520 nm, the medium refractive index is 1.33, the coverslip and immersion medium refractive index is 1.5. (b) δ_{3D}-dependence of the polarization factor $\mathrm{P} = \sqrt{P_0^2 + P_{45}^2}$ for the approximated 2D model used for the parameter retrieval ($P(\delta) = \mathrm{sinc}\,\delta$), for NA = 1.2 and NA = 1.45. The distribution is considered lying in the sample plane ($\eta = 90°$) and at the sample surface ($z = 0$) and for different distances z of the dipole emitter to the water-glass interface. (c) Sensitivity of P(δ) to the tilt angle η of the cone distribution, represented as the variation to its value for an in-plane distribution: $DP = [P(\delta, \eta) - P(\delta, \eta = 90°)]/[P(\delta, \eta = 90°)]$.

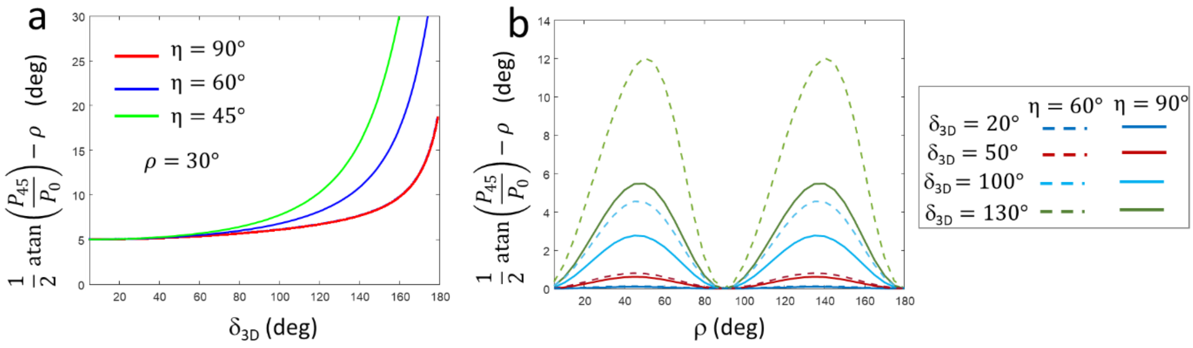

Figure S3. Principle of the retrieval of ρ from 4polar-STORM polarization factors. Dependence of the bias quantity $\left(\frac{1}{2}\ \mathrm{atan}\left(\frac{P_{45}}{P_0}\right) - \rho\right)$ in different (ρ, δ, η) conditions: (a) dependence on δ for different off-plane tilt angles η; (b) dependence on ρ for different wobbling and off-plane (δ, η) conditions.

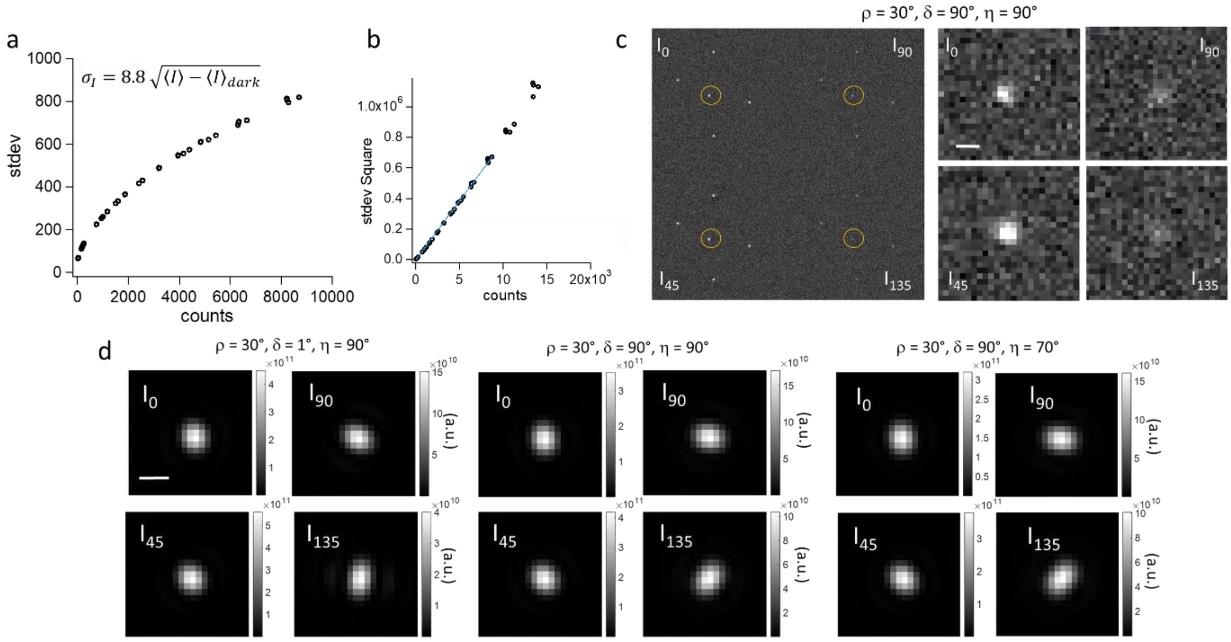

Figure S4. Camera noise estimation and Monte Carlo simulation parameters. (a) Dependence of the standard deviation σ_I of the measured signal with respect to its mean value $\langle I \rangle$, measured on 500 samples (image regions of interest), using a camera gain of 300. The measurement was performed on homogeneous images produce by white light illumination of a piece of paper. Markers: experimental data. (b) Same data represented as σ_I^2. Blue line: linear fit, found to be of slope 77.5. The equation $\sigma_I = 8.8\sqrt{I - I_{dark}}$, is thus used as a noise model for the used camera in the present working conditions. An offset of $I_{dark} = 480$ counts is systematically removed to account for the camera electronic dark counts, irrespectively of the gain and exposure time. This offset is subtracted from all data acquisitions, including calibrations. The linearity of the camera response was moreover validated in the range of signals typically measured. (c) Simulated single molecule 4polar-STORM images generated for the Monte Carlo simulations (left : full-field image, right : zooms on the single molecule

circled in the full-field image, represented in the four channels, normalized to the same signal value). The parameters used here are total intensity 1500 photons, background 60 photons/pixel (1 pixel = 130 x 130 nm^2), (δ = 90°, ρ = 30°, η = 90°). The simulation, which principle is detailed in the Methods section of the main text, is based on Gaussian-shape PSFs. Scale bar : 500 nm. d) Full simulation of single molecule images based on the propagation through the microscope considering a detection numerical aperture NA = 1.2, for different orientation parameters (δ,η) and ρ = 30°. The simulation uses models derived in[9] and[4] and includes the presence of a water-glass interface. Some of the modelled PSFs exhibit a non-Gaussian oval shape, in particular in the lowest intensity-channel. Simulations show that their shape anisotropy (ratio between largest to smallest lateral size) is lower than 1.3 as long as δ is larger than 5° (e.g. not-fixed dipoles) and η is lower than 60° (e.g. mostly in-plane molecules). These deviations are expected to lead to an increase of the retrieved averaged radius by the detection algorithm (see Supplementary Fig. S9).

Supplementary Note 2. Calibration factors in 4polar-STORM

Due to imperfections of the beam splitters as well as possible polarization leakages introduced by the optics of the detection path, correction factors need to be introduced in the estimation of the polarization factors P_0 and P_{45}. We denote G_{BS} the factor accounting for the imperfect 50:50 reflection:transmission ratio of the first non-polarizing beam splitter. We also denote G_0 and G_{45} the factors accounting for the unbalanced polarization split efficiency in the 0:90 and the 45:135 polarization channels respectively. (G_{BS}, G_0, G_{45}) are measured using a depolarized image (fluorescent solution) in which the ratios write:

$$G_{BS} = \frac{I_{45}+I_{135}}{I_0+I_{90}} \, ; \, G_0 = \frac{I_0}{I_{90}} \, ; \, G_{45} = \frac{I_{45}}{I_{135}} \qquad \text{Eq. S23}$$

We also account for possible polarization leakages between the 0:90 and 45:135 channels. The contributions are measured by polarizing the previous un-polarized image using a polarizer of known direction at the position of the back focal plane of the objective, before propagation through the dichroic filter and other propagation optics. This is performed using a controlled polarizer direction at the back focal objective plane (thin polarizer used, LPVISE100-A, Thorlabs). Denoting G_1 (resp. G_2) the leakage proportion factor of polarization channel 135° (resp. 45°) into the 45° channel (resp. 135°), and G'_1 (resp. G'_2) for the 0°/90° leakage, the final corrected expressions are:

$$P_0 = G_{BS} \cdot \frac{(1+G'_1-G'_2)\cdot I_0 - (1-G'_1+G_2)\cdot G_0 \cdot I_{90}}{(1-G'_1-G'_2)\cdot(I_0+G_0.I_{90})}$$

$$P_{45} = \frac{(1+G_1-G_2)\cdot I_{45} - (1-G_1+G_2)\cdot G_{45}\cdot I_{135}}{(1-G_1-G_2)\cdot(I_{45}+G_{45}.I_{135})}$$

Eq. S24

In an ideal optical system, $G_{BS} = G_0 = G_{45} = 1$ and $G_1 = G_2 = G'_1 = G'_2 = 0$. Deviations up to 0.2 from these numbers can be found in a real setup. In the setup used, the measured values are $G_{BS} = 1.13, G_0 = 0.97$ $G_{45} = 1.12$ and $G_1 = 0.13, G_2 = 0.15, G'_1 = 0.02, G'_2 = 0.03$. The repeatability of the measurement of these factors from day to day is found to be better than 5%. To estimate the influence of possible deviations from the true G factors in the retrieval of the angular parameters (ρ, δ), we run Monte Carlo simulations (see Fig. S4). The simulations on generated images show that even in presence of noise and background, a variation of 10% of G factors does not influence the accuracy on ρ and leads to variation of δ by 10°.

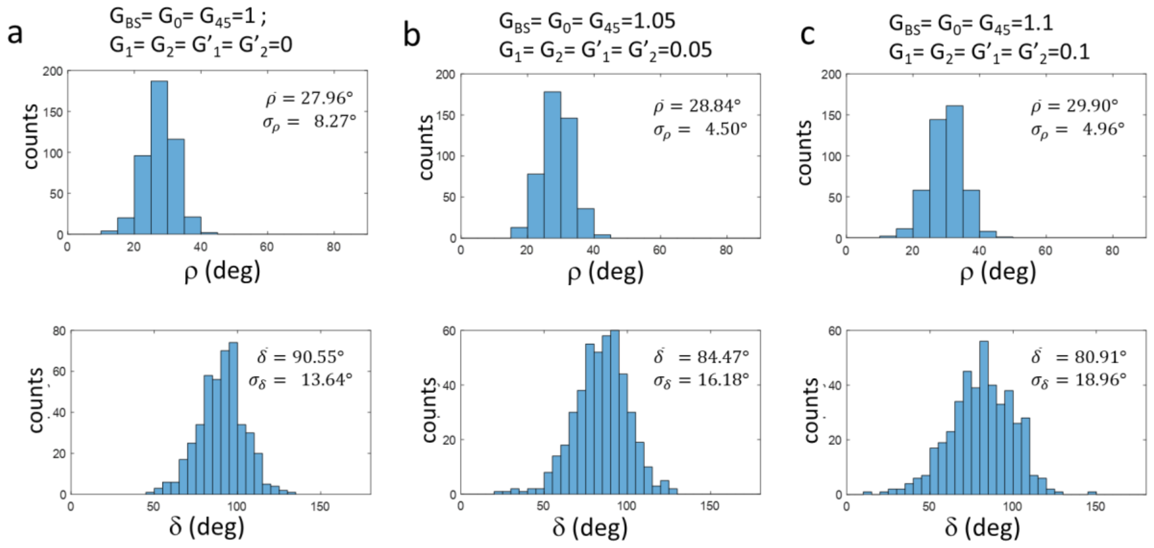

Figure S5. Simulations of the effect of variations of the G factors on the accuracy of (δ,ρ). Monte Carlo simulations are run over 500 realizations (initial conditions: 1500 photons,60 photons/pixel, η=90°, ρ=30°, δ=90°, $G_{BS} = G_0 = G_{45} = 1$, $G_1 = G_2 = G'_1 = G'_2 = 0$). (a) Retrieval under true conditions (no variation of the G factors). (b) Retrieval for a variation by 5% of the G factors. (c) Retrieval for a variation by of 10% of the G factors. The simulations are made on variations of G that influence the most the retreived parameters. The variations obtained on the retreived parameters do not strongly depend on the initial values of (ρ, δ).

Supplementary Note 3. Data processing algorithm of the 4polar-STORM method

The data processing is achieved by custom detection and analysis scripts written in Matlab, following a scheme summarized below. The scripts are based on previous work published in[8]. The previous polar-STORM algorithm, written for a two-image polarization split, has been adapted to a split into four images named (img_0, img_{45}, img_{90}, Img_{135}). Its goal is to estimate the polarization factors P_0 and P_{45} for each detected single molecule, build a super-resolved image of these polarization factors, and reconstruct an orientation super-resolved image from them. The purpose of the 4polar-STORM algorithm is thus to retrieve, per molecule, its center point spread function (PSF) position coordinates in the 4 detected images (i_0, i_{45}, i_{90}, i_{135}), (j_0, j_{45}, j_{90}, j_{135}), its PSF radius (r_0, r_{45}, r_{90}, r_{135}), its localization precision ($\sigma_{loc,0}$, $\sigma_{loc,45}$, $\sigma_{loc,90}$, $\sigma_{loc,135}$) and its PSF amplitude (α_0, α_{45}, α_{90}, α_{135}), used to calculate the

intensities (I_0, I_{45}, I_{90}, I_{135}) and thus the polarization factors P_0 and P_{45} (see Eq. S20). Once these two factors are calculated, we deduce the orientation parameters for each single molecule (in-plane orientation angle ρ and wobbling angle value δ) using Eq. S21. This algorithm offers advantages as compared to a pure ratiometric calculation based on image registration, since the pairing is realized at the molecular level for each STORM stack recorded, without any requirement of pre-calibration experiment which may add additional positioning errors and therefore bias in the polarization factor estimation. This algorithm contains several sequential steps detailed below.

Distortion correction. A calibration sample (fluorescent nanobeads) is used to correct images from distortions. Fluorescent nanobeads of 100 nm in size (yellow-green Carboxylate-Modified FluoSpheres, ThermoFisher Scientific F8803) are immobilized on the surface of a poly-L-lysine coated coverslip and covered with a mounting medium (Fluoromount, Sigma F4680). A Fluorescent nanobeads image is used to estimate the spatial transformation (*tform*) to be used between the different polarization projections to correct any possible geometrical distortion, due mainly to the use of Wollaston prisms. We selected the quadrant img_{90} as the reference image for the registration. The function *imregtform* (Matlab Imaging Processing toolbox) was used to retrieve the *tform* function for the other three quadrants (img_0, img_{45} *and* Img_{135}). This function relies on an affine transformation and is performed on typically a 10-beads image, using a point control with a minimum of 3 points/beads per image. Then we correct these images using the transformation previously estimated. The spatial transformation was re-calculated only if the optical setup was modified. The correction was performed using the Matlab function *imwarp* under a linear interpolation (Matlab Imaging Processing toolbox). Note that the transformation mentioned here is used to correct for image distortions, as a preliminary step before image analysis: it is not used to register the images since the registration procedure is part of the image analysis code.

Detection procedure. Briefly, single molecules are detected by a test of hypotheses. Each single molecule PSF is then characterized by estimating various parameters (position, background, PSF radius and amplitude, thus intensity). Maximum likelihood (ML) is used for the PSF peak detection and to evaluate the respective likelihood of having either a peak or only noise (comparison between two hypotheses assuming Gaussian noise) within the search window. The detection uses a generalized likelihood ratio test (GLRT) corresponding to a likelihood ratio test from which the parameters are analytically estimated by ML and then reintroduced in the parameter estimation. Background, intensity and variance of the noise are then retrieved from a log-likelihood of the hypotheses using partial derivatives. For the final estimation of peak parameters, optimization is performed by Gauss-Newton regression and minimization of a least squares analysis assuming a Gaussian shape PSF. For detection, the coordinates of the Gaussian center are integer pixel values and the PSF radius is fixed to a default value, while for estimation, the values of all parameters are refined with sub-pixel accuracy. A constant false alarm rate detection is used (which refers to a form of adaptive algorithm) to detect target molecules against a background of noise. Even with no target, there is always a remaining probability, the PFA (probability of false alarm), for the noise to overpass any threshold above which any signal can be statistically considered not to originate from noise. We choose a PFA inversely related to the number of pixels: this ensures that, in average, less than one spurious detection (noisy peak "accidentally" detected) can arise. At last, computing the Fisher matrix and the associated Cramér-Rao Bound (CRB) allows to estimate the theoretical lowest error expected for each PSF parameter, including background, intensity, position, radius and variance of the noise. This allows in particular estimating the lowest expected localization precision.

Detection and estimation. As in the polar-STORM algorithm[8], single molecule localizations in image quadrants (*img*$_0$*, img*$_{45}$*, img*$_{90}$*, Img*$_{135}$) are based on a first detection step which uses a Generalized likelihood ratio test (GLRT) to identify the single molecule candidates for the STORM image reconstruction, as detailed in[10]. This detection step uses a given fixed Gaussian shape for the theoretical PSF (starting from an initial guess radius of r_0 = 1.3 pixels), a spatial sliding detection window (w_s) and a limit probability of false alarm (PFA)[10], which defines a threshold limit (calculated empirically based on Monte Carlo simulations) above which any signal can be statistically considered as having a different origin than noise. A value PFA ≤ 10^{-6} is set to guarantee a probability of false alarm (PFA) of less than 1 pixel per image, which ensures a fraction of the detected single molecules close to 100% for a signal to noise ratio (SNR) higher than 20dB[10]. The Gaussian function used to model the microscope PSF for the detection step is centered at pixel positions (i_0, j_0) with a radius r_0 (standard deviation), and normalized to a power of one (squared signal):

$$G_{i,j}(i_0, j_0, r_0) = \frac{1}{\sqrt{\pi} r_0} \exp\left(\frac{-(i-i_0)^2-(j-j_0)^2}{2{r_0}^2}\right) \qquad \text{Eq. S25}$$

r_0 is larger than one pixel, which satisfies adequate sampling according to the Nyquist-Shannon theorem.

After the candidates have been detected by the GLRT algorithm, the amplitude, radius and position of their Gaussian PSF are estimated on all four quadrants at the subpixel level, based on a Maximum likelihood (ML) estimation using a Gauss-Newton regression. This regression uses the GRLT obtained values of radius r_0 and position (i_0, j_0) as initial parameters. Following the Gaussian model used in Eq. S25 for the PSF, the signal from each single molecule centered in (i_n, j_n) is written:

$$\alpha_n \times G_{i,j}(i_n, j_n) + N_n + b \qquad \text{Eq. S26}$$

Where b is the background, N_n the noise, α_n the amplitude of the PSF. The parameters (i_n, j_n), α_n , b and the standard deviation of the noise N_n are all estimated from the ML estimates.
Note that due to the normalized squared power form used for Gaussian function in Eq. S25, the integrated intensity over the PSF surface is, for a PSF or radius r:

$$I_n = 2\sqrt{\pi} r \alpha_n \qquad \text{Eq. S27}$$

Typical fits from experimental and Monte Carlo-generated PSFs are shown in Figure S6, under different background and signal conditions.

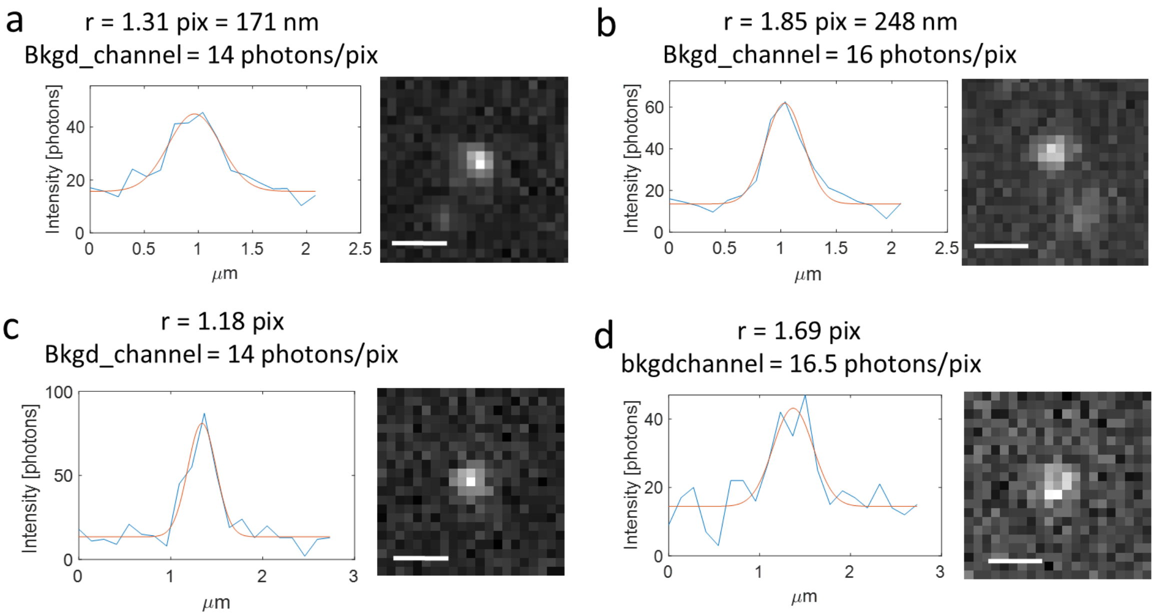

Figure S6. Fit of typical point spread functions in simulated and experimental images. (a,b) Experimental PSFs selected in the I_{45} image, taken from a fixed actin-labelled cell. The profiles represent the Gaussian fit obtained in one dimension, for two cases of relatively standard (a) and large (b) radius. (c,d) Monte Carlo generated PSFs and their 1D Gaussian fits. (c) low radius fit (initial parameters: total intensity 1500 photons, background 60 ph/pixel, radius 1.3 pixels). (d) large radius fit (same parameters except radius, radius 1.7 pixels). The parameters of the fits are given above the fit graph, with bkgd_channel indicating the background value in the specific image/channel. Scale bar 780 nm.

Computing the Fisher matrix and the associated Cramér Rao bond allows to estimate the theoretical lowest error expected for each PSF parameter as detailed in[10], in particular the localization precision σ_{loc}. This quantity is computed for all four images along the four polarization projections.

Estimation of the translation vector between image quadrant pairs: (img_0 - img_{90}), (img_{45} - Img_{135}) and (img_0 - img_{45}). To estimate the translation vector between the different images and associate each detected PSF to a given molecule, it is possible to use the registration from the bead sample used for the distortion-correction step above. This type of registration is however limited by the camera pixel size, image quality and stability of the optical system. It also implies the use of interpolation methods during the image subsampling. The 4polar-STORM software rather directly calculates the translation vector using the detected molecules themselves, which are localized with high precision. The distance of each molecules' images in the quadrant pairs *(img_0 - img_{90}), (img_{45} - Img_{135})*, and *(img_0 - img_{45})* created by the Wollaston polarization beam splitter prisms is represented by three vectors ($\vec{u}_{0-90}$, $\vec{u}_{45-135}$, $\vec{u}_{0-45}$). The knowledge of these translational vectors is required for the detection of the molecule-pairs present in a STORM image stack. To estimate the vectors, we perform a statistical estimation by using the 100-1,000 first frames of the STORM recorded stack[8]. First, all possible vectors joining two molecules of the images are calculated and the squared norms of the differences between all the obtained vectors are calculated. This leads to a statistical distribution, within which only the candidates whose difference is below 4.2 times the obtained standard deviation are kept (pure

significant test assuming that the difference between norms follows a Gaussian distribution, which guarantees a 95% confidence level to have similar directions between the selected pairs of PSF images). Second, an additional selection is performed to determine the most optimal vector. This second step compares the obtained vector squared-norms and keeps the largest ensemble of similar squared-norms in this population. For this a sub-optimal detection is run (the only hypothesis being that the error on position is Gaussian), keeping errors only below a threshold that guaranties a 95% confidence level within the obtained distribution.

Association of molecule pairs. After the three optimal vectors ($\vec{u}_{0-90}$, $\vec{u}_{45-135}$, $\vec{u}_{0-45}$) are calculated, pairs of molecules along these directions are coupled by selecting the nearest neighbor to the expected position at a vector distance from the reference quadrant, within a distance tolerance corresponding to the localization precision. First, molecules from the images pairs (img_0 - img_{90}) and (img_{45} - img_{135}) are coupled separately over the full STORM stack (typically 30 000 – 50 000 images), then the identified couples are associated using $\vec{u}_{0-45}$.

Estimation of molecular parameters. The pairing of all molecules allows the reconstruction of a polarization STORM image based on the parameter position (i_0, i_{45}, i_{90}, i_{135}), (j_0, j_{45}, j_{90}, j_{135}), PSF radius (r_0, r_{45}, r_{90}, r_{135}), localization precision ($\sigma_{loc,0}$, $\sigma_{loc,45}$, $\sigma_{loc,90}$, $\sigma_{loc,135}$) and PSF amplitude (α_0, α_{45}, α_{90}, α_{135}). Only molecules that were presented in all four-quadrants were considered for analysis to avoid bias in the orientation and intensity estimation. Accounting for molecules which exhibit a 'zero-intensity' (e.g. intensity below the detection threshold) in a given channel might artificially enrich the measured ρ population along the direction of the polarized channels (0°, 45°, 90°, 135°) directions, for which δ is badly determined, due to the fact that a much lower intensity along a channel is estimated with less precision. This population of 'zero-intensity' molecules along a given channel is associated to very low intensity in some channels for which the localization precision, PSF size and intensity are poorly estimated. In addition, δ is estimated with potential errors due to the lower precision on the intensity estimation on this 'zero-intensity' channel. Excluding 'zero-intensity' molecules along a given channel permits thus to avoid biases induced by these poor estimations (see Fig. S7). A threshold in intensity and σ_{loc} (see main text) also retrieves similar statistics as excluding 'zero-intensity' molecules (see Fig. S7). Monte Carlo simulations confirm that 'zero-intensity' molecules suffer from a larger bias on δ for the same reasons of poorly estimated intensities, especially at low δ values (δ < 60°) (see Fig. S7). In the present conditions (δ ~ 90°), the statistics on ρ and δ obtained from simulations are not significantly changed if we exclude or include the 'zero-intensity' molecules (see Fig. S7). In situations where δ is low however, it is advised to run a detection which excludes 'zero-intensity' molecules in order to estimate δ.

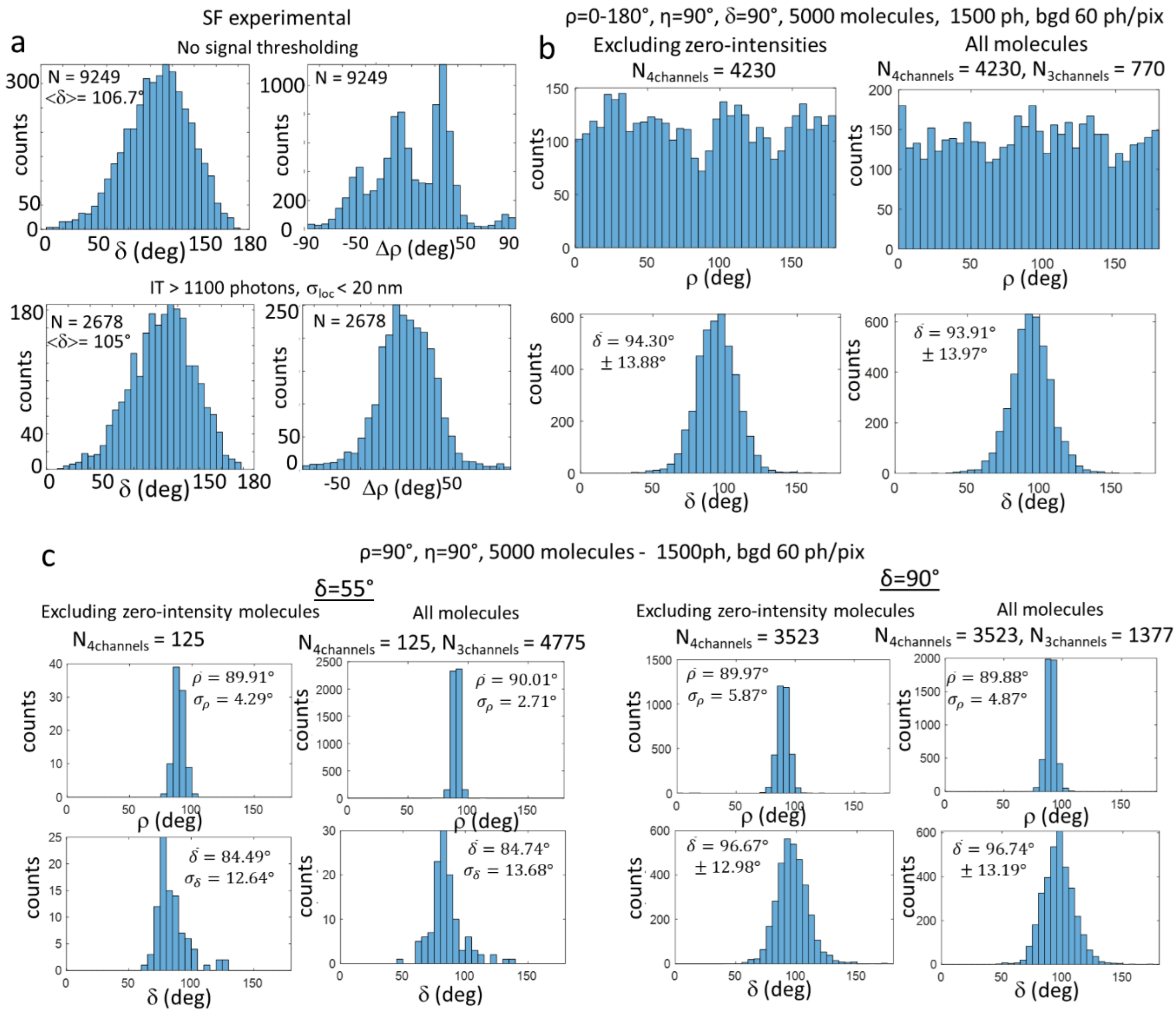

Figure S7. Effect of the detection of zero-intensities molecules on the (δ,ρ) retrieval. (a) Experimental values of (δ,ρ) obtained in a stress fiber region of a fixed cell. The region represented is the ROI1 of Figures 2 and 3 in the main text. Upper graphs : (δ,ρ) histograms including all detected molecules in a given polarized channel. Note that δ can be estimated only in a reduced set of the detected molecules, as compared to ρ. Lower graph : same data, thresholded in intensity and σ_{loc}, . Identical ρ histograms are obtained when excluding molecules which exhibit a zero-intensity in a given polarized channel. On average, averaged numbers found in studied ROIs in stress fibers are ~ 9000-9500 total molecules detected, ~ 4000-5000 molecules detected in all four channels, 2500-3500 of high accuracy and precision (e.g. kept by intensity and localization thresholding). (b) Monte Carlo simulation of an ensemble of random oriented molecules with δ = 90°. Left : not-including zero-intensities molecules. Right : accounting for zero-intensities molecules. (c) Left : Monte Carlo simulation of molecules oriented with ρ = 30° and δ = 90°. Right : Similar situation for δ = 55°. For low δ values, an increased portion of directions are missed along the direction of the polarized channels, but δ is estimated with a high bias (up to 30° bias for δ ~ 55°). At larger δ values (e.g. 90°), there is a ~50% portion of molecules missed and the histograms are unchanged.

Calculation of polarization factors P_0 and P_{45}. Polarization factors P_0 and P_{45} (see Supplementary Note 1) are calculated based on the integrated intensities I_0, I_{45}, I_{90} and I_{135}, which are calculated from (α_0, r_0), (α_{45}, r_{45}), (α_{90}, r_{90}) and (α_{135}, r_{135}) as shown in Eq. S27.

After intensities are estimated, the polarization factors (P_0, P_{45}) are deduced, accounting for the calibration correction factors as detailed in Eq. S24 (see Supplementary Note 2).

Estimation of ρ and δ per molecule. After the polarization factors are calculated, ρ and δ are estimated based on the expressions given in Eq. S12. To solve the determination of δ, an interpolation is performed using the function "*interp1*" of Matlab.

Postprocessing and visualization. Postprocessing is performed in a modified version of PALMsiever[11], a visualization and analysis platform for single-molecule localization microscopy implemented in Matlab. It uses the software package DIPimage (https://diplib.org/DIPimage). PALMsiever includes a plugin for drift correction using cross-correlation. For this work, we used two rendering modalities: histogram + Gaussian filter and Kernel Density Estimation (KDE)[12]. KDE is a smoothing version of histogram, with the smoothing kernel bandwidth estimated from the molecule density. Our version of PALMsiever of the 4polar-STORM modality includes the estimation of ρ and δ, as well as their graphical representation as sticks. In this representation, ρ is depicted as the orientation angle of sticks (with respect to the horizontal axis of the image), and δ or ρ are depicted as the colors of the sticks. The sticks are displayed over a black-and-white image representing the super-resolved STORM image, which uses the localization of all detected molecules. 4polar-STORM allows to use the default parameter filters of PALMsiever for the image representation, filtering for instance molecule populations by their density, intensity and localization precision. An example of 4polar-STORM representation is shown below.

Representation of ρ and δ in STORM images. The final 4polar-STORM representation consists in depicting, over a STORM image background, one stick per single molecule detected, whose orientation relative to the horizontal axis is ρ, and whose color is either encoding ρ or δ. In the chosen representation, we plot sticks with largest δ values above sticks with lowest ones, in order to better visualize the presence of highly wobbling populations in red.

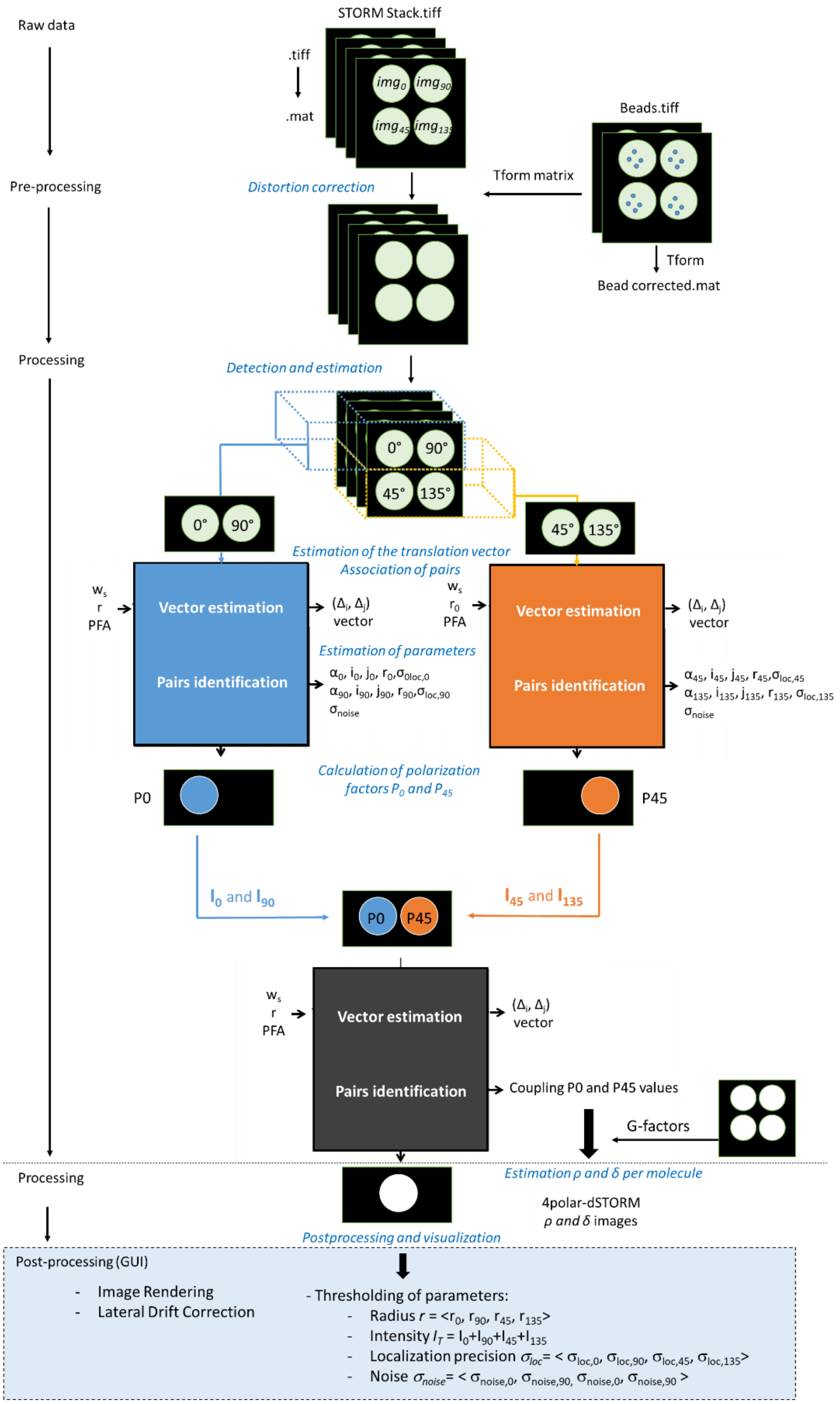

Figure S8. 4polar-STORM algorithm flowchart. In the pre-processing step, the raw data (.tiff image stack files) is transformed into ".mat" files to be further spatially corrected by the bead calibration in Matlab. The distortion-corrected stack files are processed for each Wollaston polarized arm (in blue, the pair 0°-90°, and in orange, the pair 45°-135°). On each arm, the 4polar-STORM algorithm is applied: GLRT is applied to find the best translation vector, which is further used to identify coupled blinking

pair events. A multi-parametric Gaussian-Newton fit is performed to retrieve quantitative parameters of the blinking particles, such as position (i,j), localization precision (σ_{loc}), the amplitude (α), radius (r), and noise (σ_{noise}). A vector estimation and pair identification (coupling) is done to group the information into polarization factor ratios P_0 and P_{45}. The last step of the data treatment is to calculate the orientation parameters for each blinking particle in the reconstructed image. For that, the G-factors (i.e., the intensity calibration correction) are applied. Post-processing is performed using a derivation of PALMSiever[11]. The most common post-processing steps used for 4polar-STORM are: (1) lateral drift correction (based on cross-correlation with the localization themselves), (2) choice of image rendering, (3) parameter thresholding for robust orientation parameter estimation, and (4) choice of the stick representation.

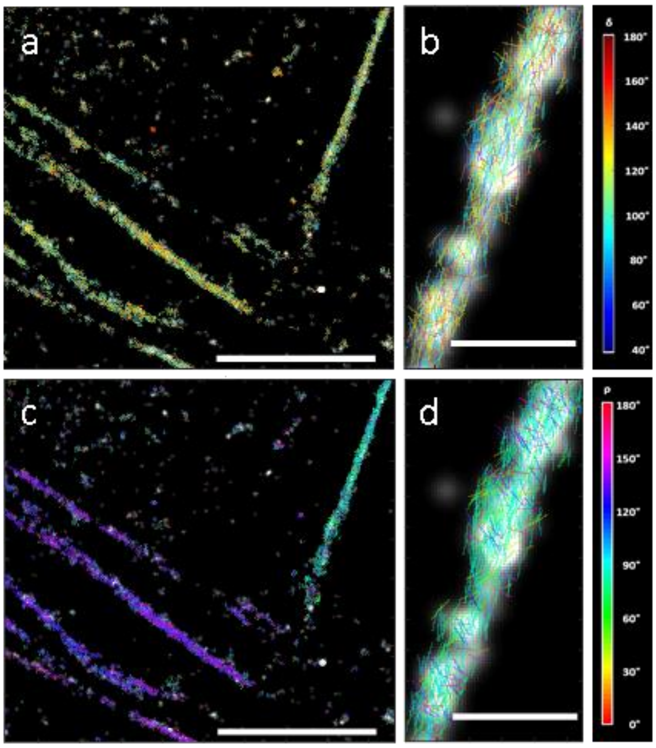

Figure S9. 4polar-STORM stick representations of (δ,ρ). Example of super-resolved image of δ (a,b) and ρ (c,d) using 4polar-STORM on stress fibers of a U2OS cell stained for F-actin (AF488-phalloidin). Stick orientation is based on ρ. Colors of the sticks correspond to δ (a,b) and ρ (c,d). Scale bars: 5 µm (a and c) and 500 nm (b and d). Gaussian blurring size: 39 nm. A density filter was applied to remove isolated spots. The principle of this density filter is based on the PALMsiever algorithm tool https://github.com/PALMsiever/palm-siever/wiki/density_column), to remove isolated PSFs from the image, 'isolated' meaning at a distance larger than the pixel size of the image (here, 24.86 nm). Rendering pixel size: 12.42 nm (b,d), 24.86 nm (a, c).

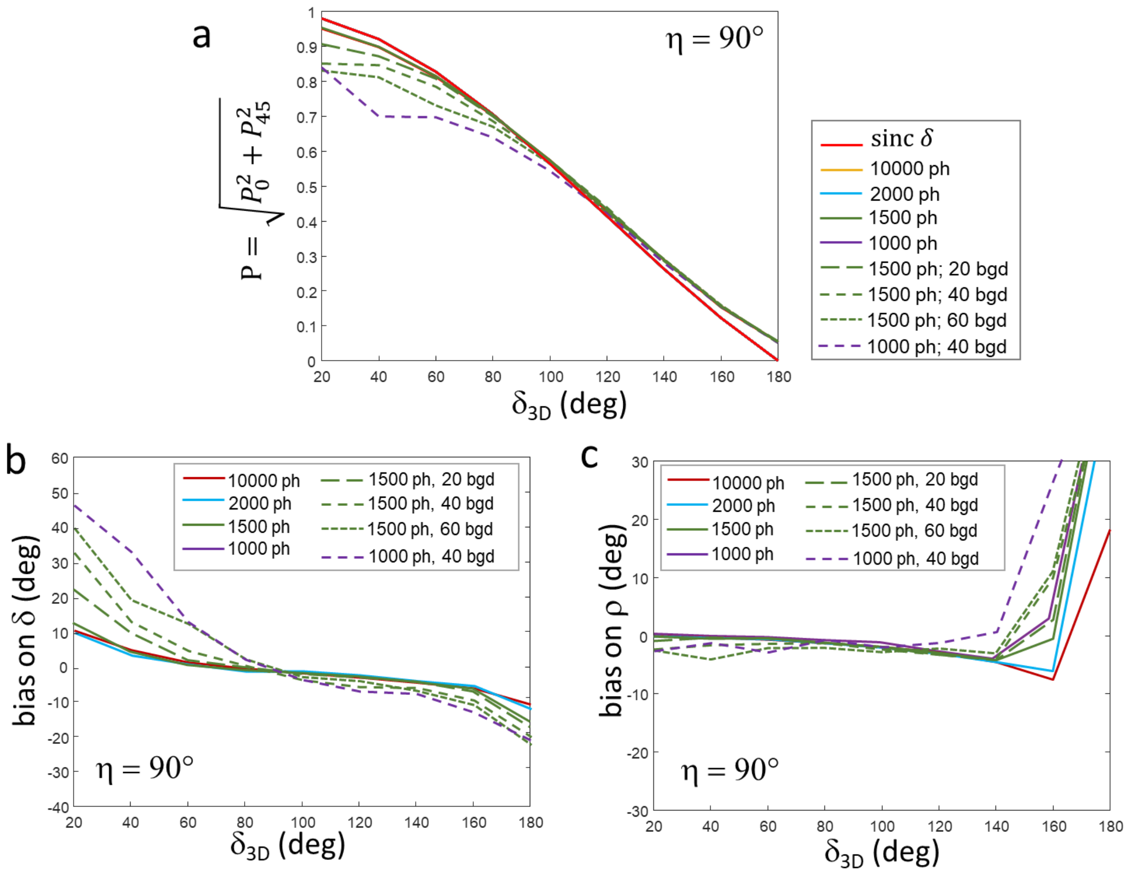

Figure S10. Monte Carlo simulations of the effect of the total intensity and presence of background on the accuracy of (δ,ρ) at η=90°. The Monte Carlo simulations are run on 500 realizations, for $\rho = 30°$. (a) Dependence of $P = \sqrt{P_0^2 + P_{45}^2}$ to intensity and background conditions. (b) Consequent dependence of the bias on δ estimated from P. (c) Dependence of the bias on ρ estimated from $\frac{1}{2}$ atan $\left(\frac{P_{45}}{P_0}\right)$. The intensity/bacground-dependence depicted in these graphs is independent on ρ. The numbers given are in photons for the intensity, and in photon/pixel for the background.

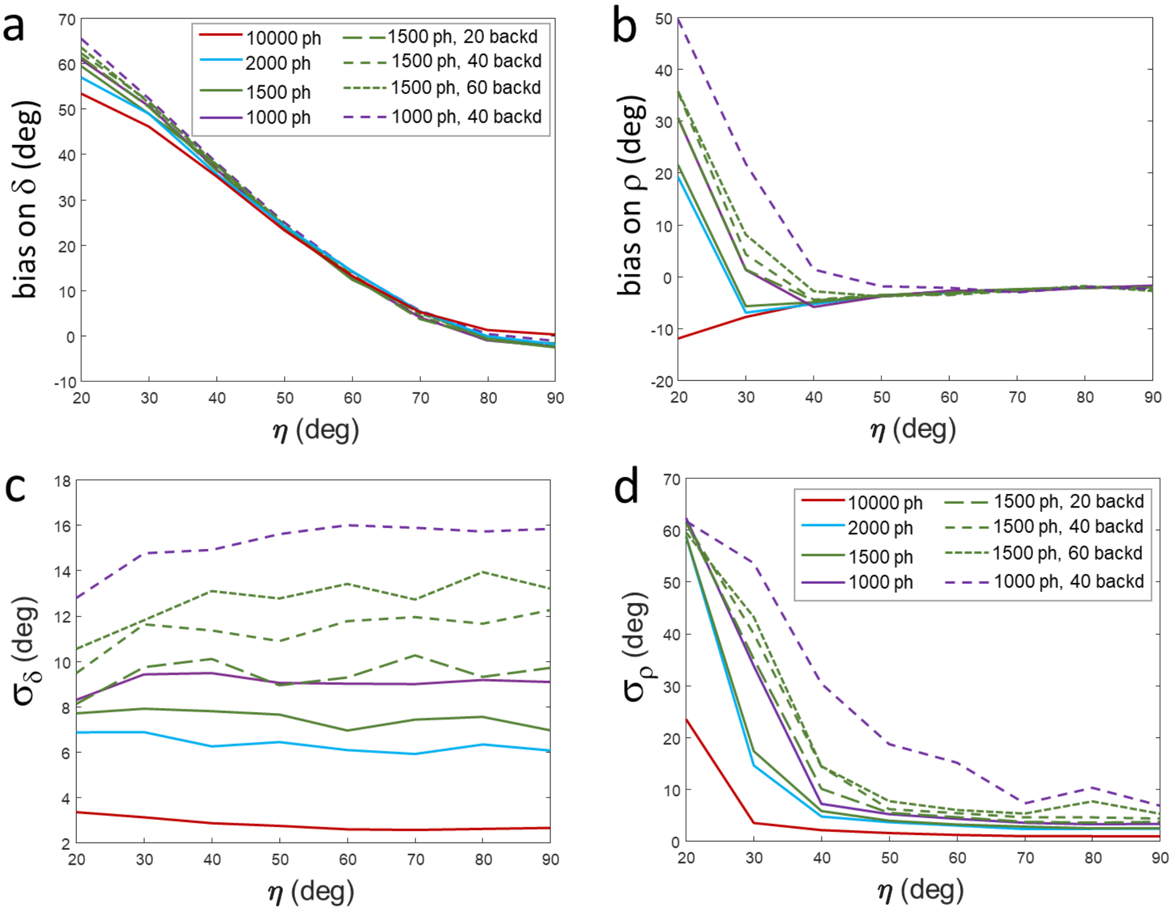

Figure S11. Monte Carlo simulations of the effect of the intensity and background on the accuracy and precision on (δ,ρ) for δ=100°, ρ=30°, at variable η. The Monte Carlo simulations are run on 500 realizations for δ=100° and $\rho = 30°$. (a) Bias on δ. (b) Bias on ρ. (c) Error (standard deviation) on δ. (d) Error (standard deviation on ρ.

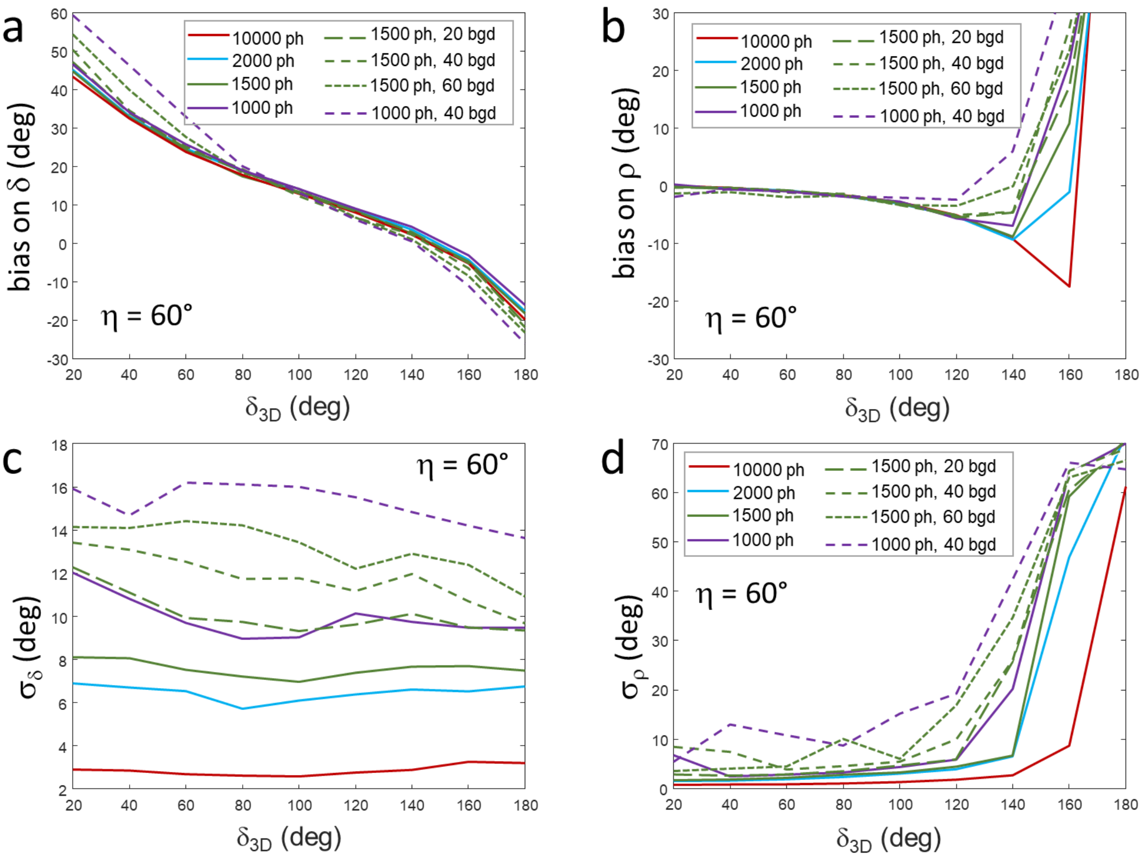

Figure S12. Monte Carlo simulations of the effect of the total intensity and background on the accuracy of (δ,ρ) at variable δ for η = 60°. The Monte Carlo simulations are run on 500 realizations, for $\rho = 30°$. (a) Bias on δ. (b) Bias on ρ. (c) Error (standard deviation) on δ. (d) Error (standard deviation on ρ. The intensity/bacground-dependence depicted in these graphs is independent on ρ.

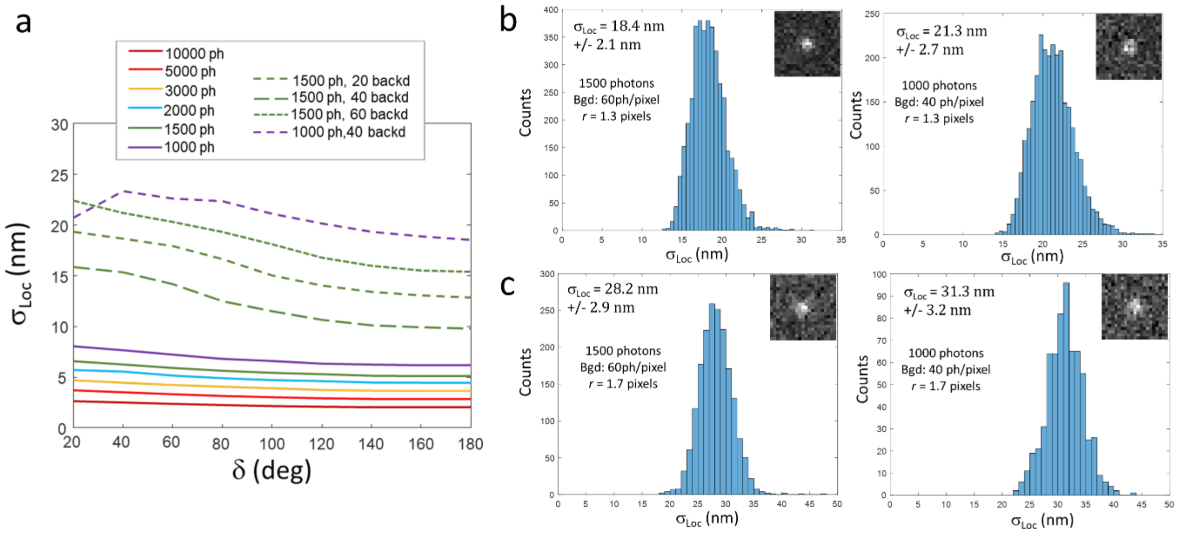

Figure S13. Monte Carlo simulation of the effect of the total intensity and background on the localization precision and retrieved radius of single molecule's PSFs. (a), localization precision (σ_{loc}) for various intensity-background conditions and δ values, at ρ = 30°, η = 90° (Monte Carlo simulations are performed on 500 realizations). (c) Examples of histograms retrieved for σ_{loc} in two signal-background conditions for a PSF radius of 1.3 pixels (Monte Carlo simulations are performed on 5000 realizations, covering all ρ values at δ = 90°, η = 90°). Typical single molecules PSFs generated from the simulations are shown in the insets (a pixel size of 130 nm is used for the calculation of σ_{loc}). (c) Same simulations, for a radius of 1.7 pixels.

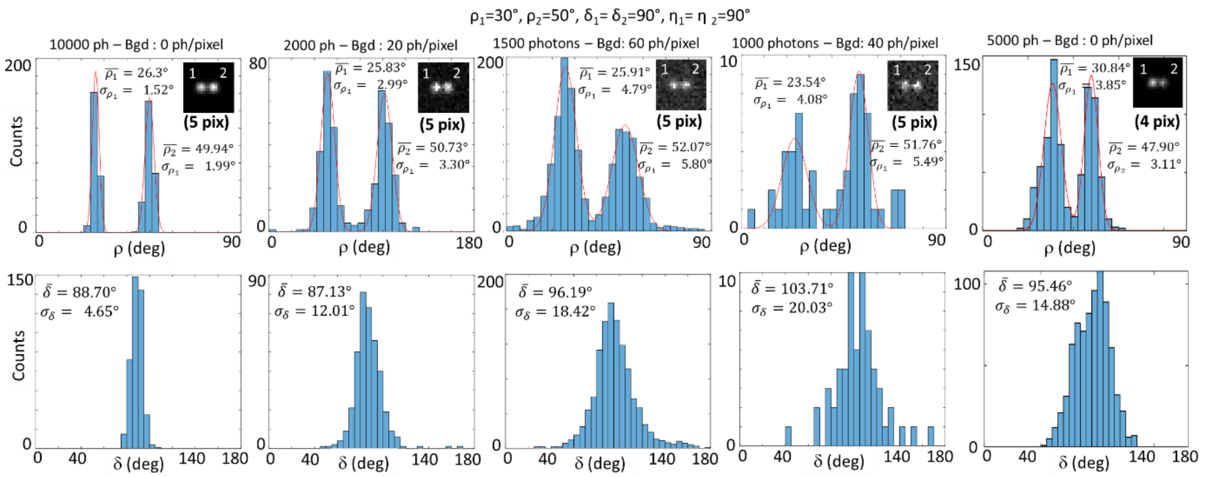

Figure S14. Effect of the proximity of single molecules on the orientation retrieval efficiency. Simulations were performed on two single PSFs (named 1 and 2) whose centers are distant by 5 pixels (650 nm) or 4 pixels (520 nm). Conditions used for molecules 1 and 2: ρ_1=30°, ρ_2=50°, δ_1= δ_2=90°, η_1= η_2=90°. The right panel is depicted for a 4 pixels distance between molecules 1 and 2, where it was necessary to use a smaller detection window than used in this work (7 pixels instead of 13). The insets show typical generated images of molecules 1 and 2 in different signal and background conditions (45° polarized image). The red lines on the ρ histograms are double-Gaussian whose fitting mean and width are given on the graphs.

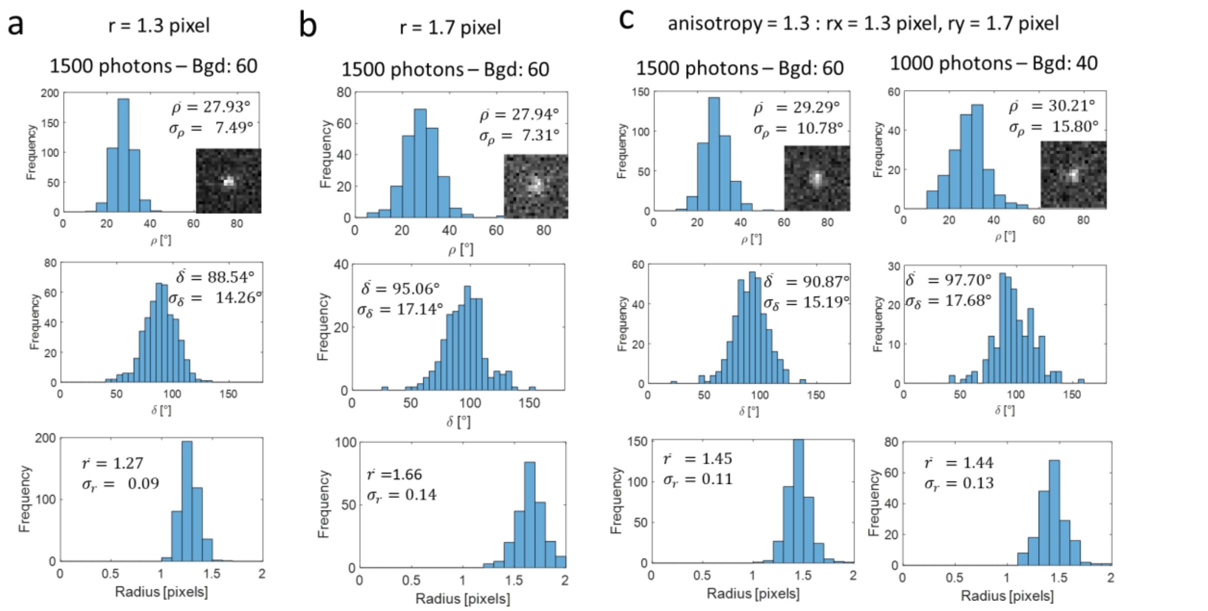

Figure S15. Effect of the deformation of single molecule PSFs, on the orientation retrieval efficiency. Simulations were performed on single PSFs (500 realizations per condition). Conditions used: total intensity 1500 photons, background 60 photon/pixel ; ρ=30°, δ=90°, η=90°. (a) PSF Gaussian shape of initial radius r = 1.3 pixel. (b) PSF Gaussian shape of initial radius r = 1.7 pixel. (c) PSF anisotropic Gaussian shape of different radii in orthogonal directions x and y : rx = 1.3 pixel and ry = 1.7 pixel . For this condition the extreme signal condition 1000 photons; background 40 ph/pixels, is also shown. The insets show typical generated images of single molecules in different conditions. Radii are given in pixels (1 pixel = 130 nm).

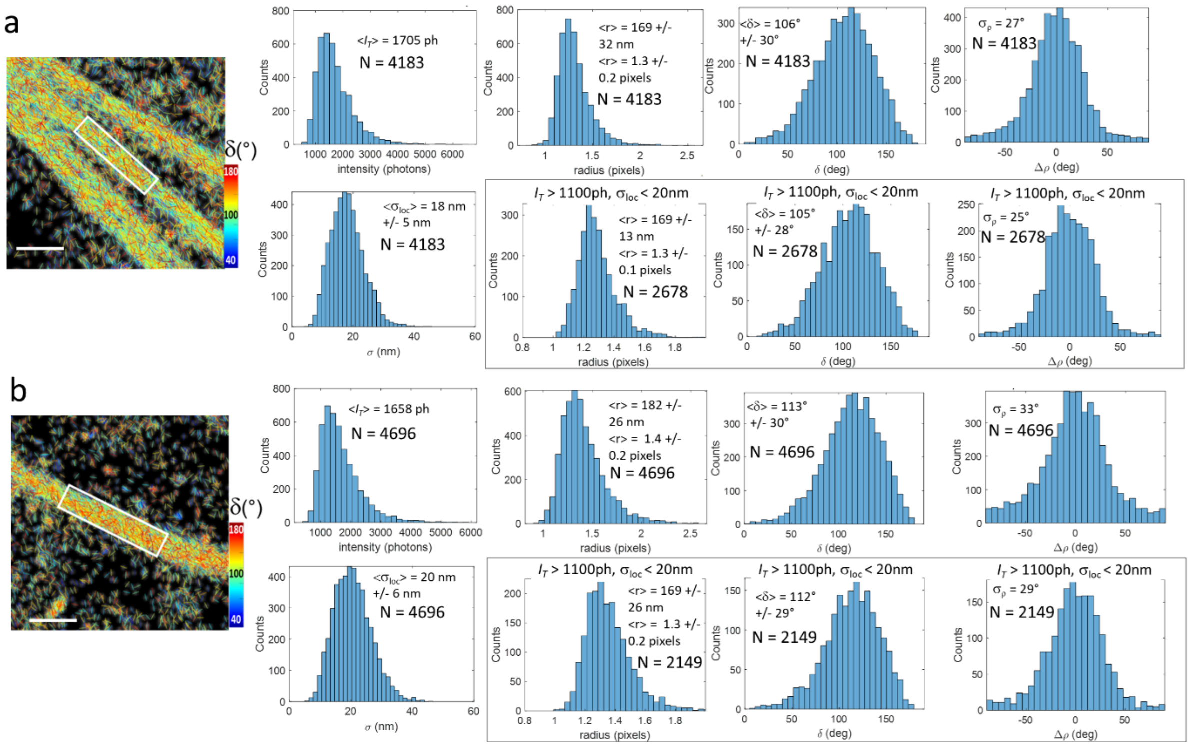

Figure S16. Statistics on detection parameters in 4polar-STORM imaging of F-actin in stress fibers in cells. Histograms of the detection parameters in two types of stress fibers in U2OS cells labelled with AF488-phalloidin (shown as the inside of regions of interest (ROI) marked by a white rectangle contour in the δ-stick 4polar -STORM images). (a) In-plane ventral SF region. (b) Focal adhesion region. Scale bars: 800 nm. The mean and/or standard deviations of the histograms are shown as insets, as well as the number N of detected molecules. Histograms shown in separate boxes are obtained by thresholding intensities higher than 1100 photons and localization precisions lower than 20nm. This is shown to keep about 50-60% of the total number of detected molecules. Source data are provided as a Source Data file (https://doi.org/10.6084/m9.figshare.17167001).

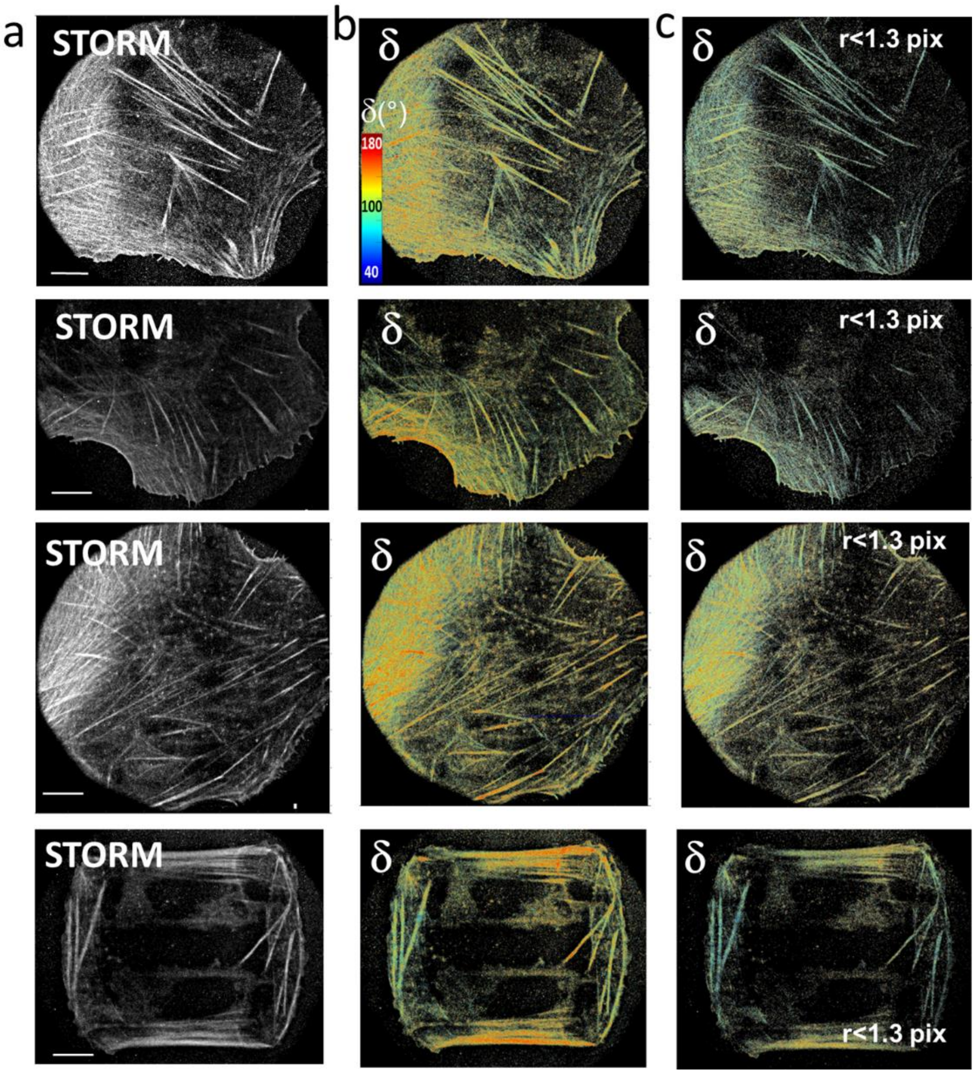

Figure S17. 4polar-STORM δ images of F-actin in fixed U2OS cells labelled with AF488-phalloidin. (a) STORM images. (b) Corresponding δ images with no thresholding of the detection parameters. (c) Corresponding δ images keeping only molecules for which the PSF radius r is below 1.3 pixels (169 nm). δ color scales are the same for all images. Scale bars 6.5 μm.

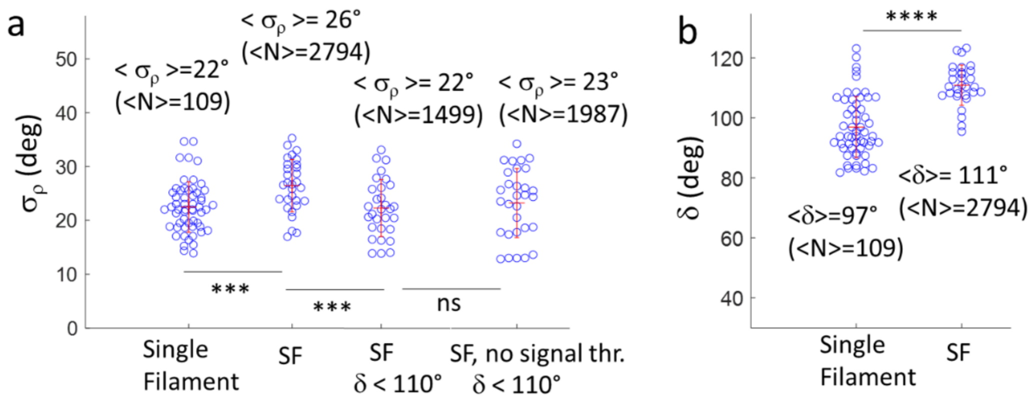

Figure S18. Comparison of δ and ρ values in single filaments and stress fibers in fixed cells. Each marker is a measurement averaged over a single region of interest (ROI). The ROIs are straight lines along the single filaments or stress fibers (SFs) of about one to a few µm length. (a) σ_ρ values using a thresholding of the intensity (> 1100 photons) and localization precision (σ_{loc} < 20 nm). "Single filament": 10 independent filaments were examined, giving a total number n=56 of measured ROIs. On average, <N> = 109 molecules per ROI were measured. "SF": 10 independent cells were examined, giving a total number n=59 of measured ROIs. On average, <N> = 2794 molecules per ROI were measured. "SF δ < 110°": same SF regions as "SF", in which only molecules for which δ < 110° are selected (e.g. filaments lying in the sample plane). On average, <N> = 1499 molecules per ROI were measured. "SF, no signal thr. δ < 110°": same SF regions in which only molecules for which δ < 110° with no intensity and localization precision thresholding. On average, <N> = 1987 molecules per ROI were measured. This category is seen to be quite similar to the one where intensity and localization precision are thresholded. The red bars represent mean values +/- standard deviation. Statistical significance of scatter plots: ns (p>0.05); ** (p < 0.01); *** (p < 0.001); **** (p < 0.0001) (statistical test used : two-sided unpaired two-sample T-test). P values from left to right: p = 2.62e-4, 6.67e-4, 0.62. (b) δ values averaged over all molecules of each ROI, in the "Single Filament" and "SF" populations of (a). The data shown are the average of σ_ρ and δ as well as <N>, the average number of molecules detected in the explored ROIs. P value: p = 7.5e-7. Typical averaged numbers found in studied ROIs in stress fibers are ~ 4000-5000 molecules detected in all four channels, 2500-3500 of high accuracy and precision (e.g. kept by intensity and localization thresholding), and 1500-2500 molecules in 2D populations (e.g. δ < 110°). Source data are provided as a Source Data file (https://doi.org/10.6084/m9.figshare.17167001).

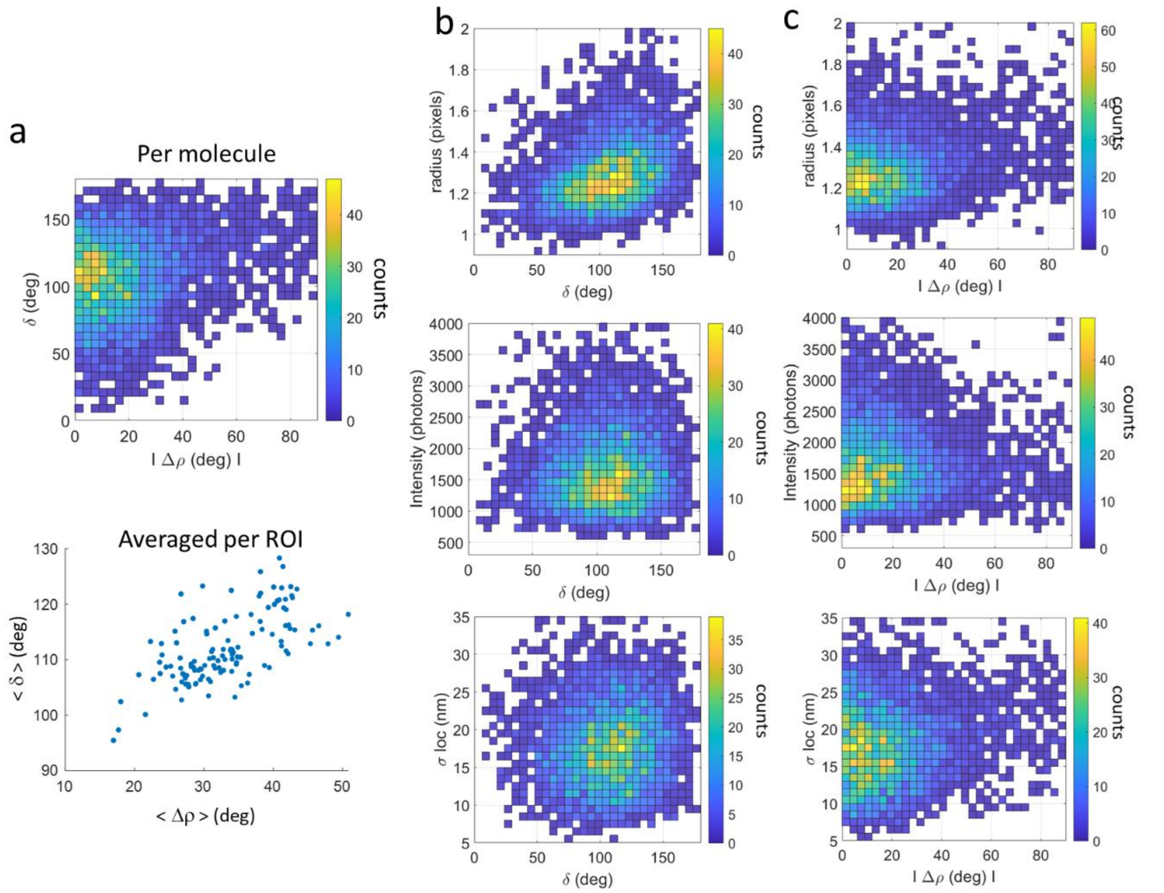

Figure S19. Correlation between the different detection parameters and the δ and ρ parameters. (a) Correlation between δ and $|\Delta\rho|$, in a population made of many single molecules within a single SF ROI Upper graph : ROI1 in the cell represented in figures 2 of the main text. Data are represented as a 2D histogram. Lower graph: population of many SFs ROIs within 10 cells. Data are represented as markers, each measured ROI being represented by its averaged δ and $|\Delta\rho|$, as a marker's coordinates. (b) Correlation between δ and the detection parameters PSF radius (upper graph), intensity (middle graph) and localization precision σ_{loc} (lower graph). Data are represented as 2D histograms. c) Similar graphs showing the correlation between $|\Delta\rho|$ and the detection parameters. Source data are provided as a Source Data file (https://doi.org/10.6084/m9.figshare.17167001).

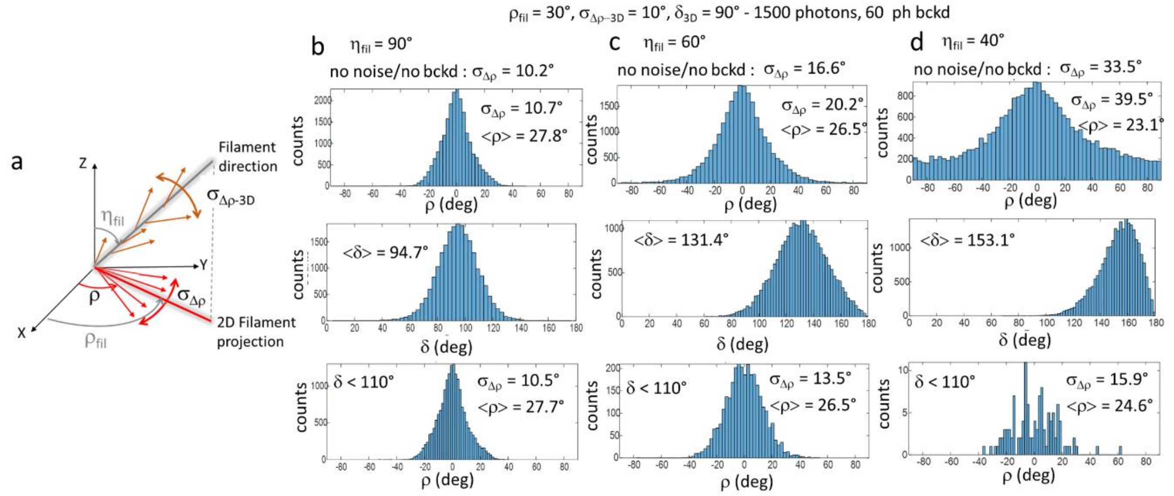

Figure S20. Retrieval bias on $\sigma_{\Delta\rho}$. (a) Schematic representation of an actin filament oriented in plane by an angle ρ_{fil} and tilted off-plane by an angle η_{fil}, with a distribution of single molecule orientations represented by $\sigma_{\Delta\rho\text{-}3D}$. The measured distribution of projected orientations in 2D is represented by $\sigma_{\Delta\rho}$. (b-d) Monte Carlo simulations for an expected cone aperture $\sigma_{\Delta\rho\text{-}3D}$ = 10°, mean orientation of the filament (ρ_{fil} = 30°, η_{fil} variable) and wobbling angle δ = 90° for each single molecule. The simulations are run over 100 realizations of a distribution of 252 single molecules present in the distribution, supposing a total intensity of 1500 photons and background per pixel of 60 photons. (b) η_{fil} =90°. (c) η_{fil} = 60°. (d) η_{fil} = 40°. Top: ρ values (centered with respect to the average $<\rho>$) retrieved from 4polarSTORM. Middle: δ values retrieved from 4polarSTORM. Bottom: ρ values (centered with respect to the average $<\rho>$) retrieved for the population δ < 110°. The standard deviation $\sigma_{\Delta\rho}$, averaged $<\rho>$ and $<\delta>$ values are given in insets for the corresponding histograms. Data from molecules for which δ < 110° show results that are very close to in-plane filaments, whatever their initial orientation η_{fil}.

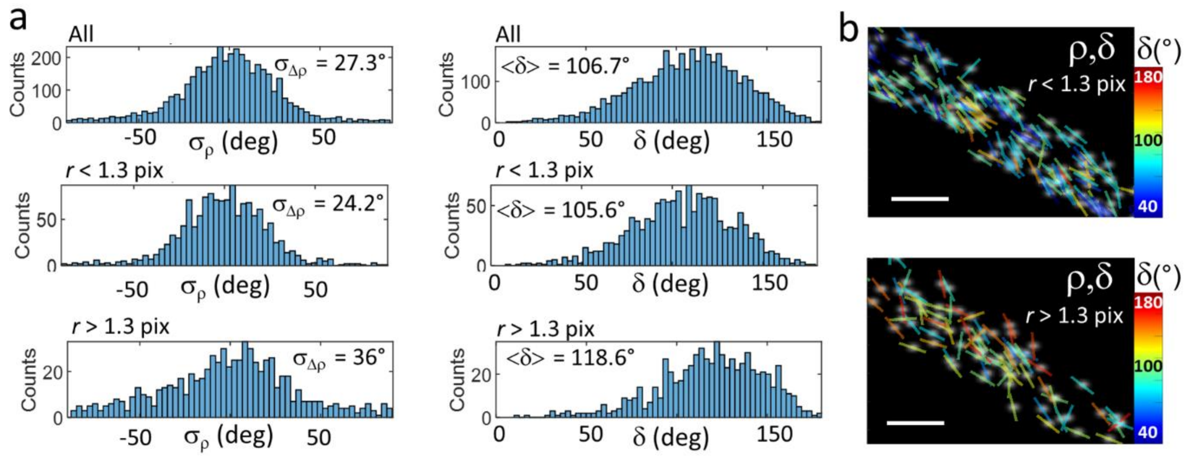

Figure S21. Effect of the measured PSF radius on the δ and ρ statistics. The ROI measured is the ROI1 in the cell represented in figures 2,3 of the main text. (a) Statistics with selection of low PSF radius (r<1.3 pixels) vs high PSF radius (r>1.3 pixels). The standard deviation of ρ and average value of δ are given in the insets. (b) Zoom on the 4polar STORM sick figures obtained with the single molecule's selection mentioned on the graph. Scale bar = 260 nm. The comparison between those statistics is similar when considering or not intensity and localization precision thresholding. Source data are provided as a Source Data file (https://doi.org/10.6084/m9.figshare.17167001).

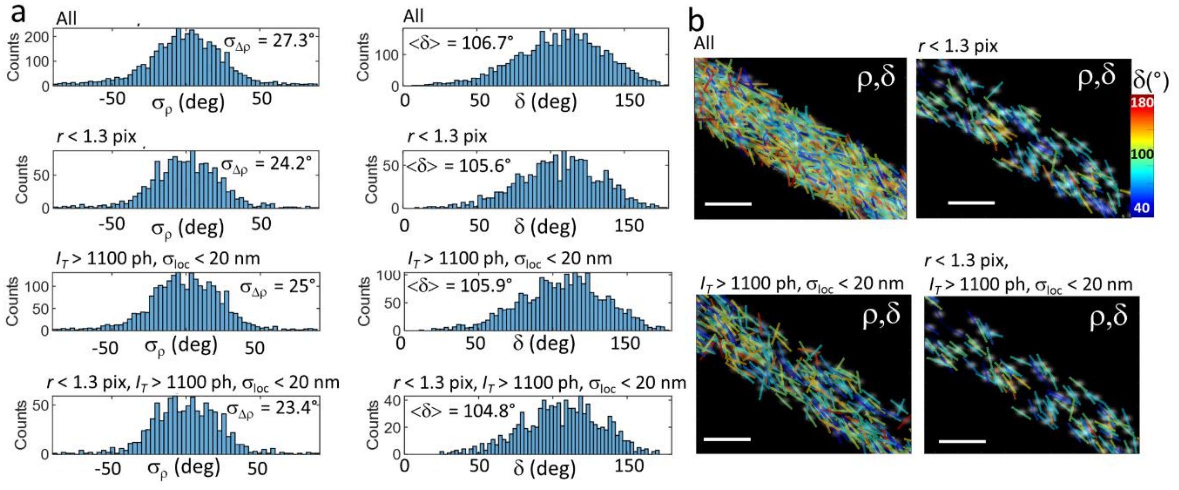

Figure S22. Effect of the intensity, σ_{loc} and *r* on the δ and ρ statistics. The ROI measured is the ROI1 in the cell represented in figures 2,3 of the main text. (a) Statistics with/without selection of total intensity > 1100 photons, σ_{loc} < 20nm with/without low (r<1.3 pixels) PSF radius selection. (b) Zoom on the 4polar STORM sick figures obtained with the single molecule's selection mentioned above the images. Scale bar = 260 nm. Source data are provided as a Source Data file (https://doi.org/10.6084/m9.figshare.17167001).

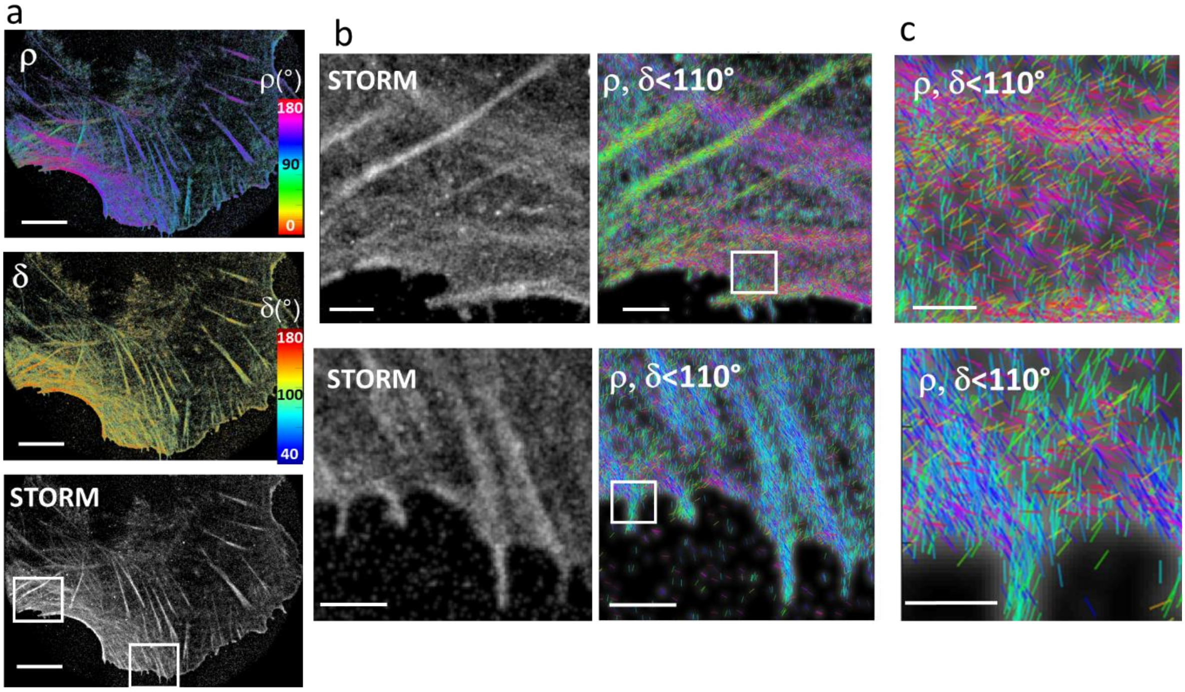

Figure S23. 4polar-STORM imaging of F-actin in cells, selecting in-plane actin filament populations. (a) Large field of view images of ρ and δ sticks as well as the corresponding single molecule localization STORM image of a U2OS cell labelled with AF488-phalloidin. (b) zoomed regions (see squares in (a)) depicting STORM and ρ-stick images for in-plane molecules only (δ < 110°). (c) stronger zoom (see squares in (b)) depicting ρ sticks for in-plane molecules only (δ < 110°). Scale bars (a) 6.5 μm; (b) 1.3 μm; (c) 500 nm.

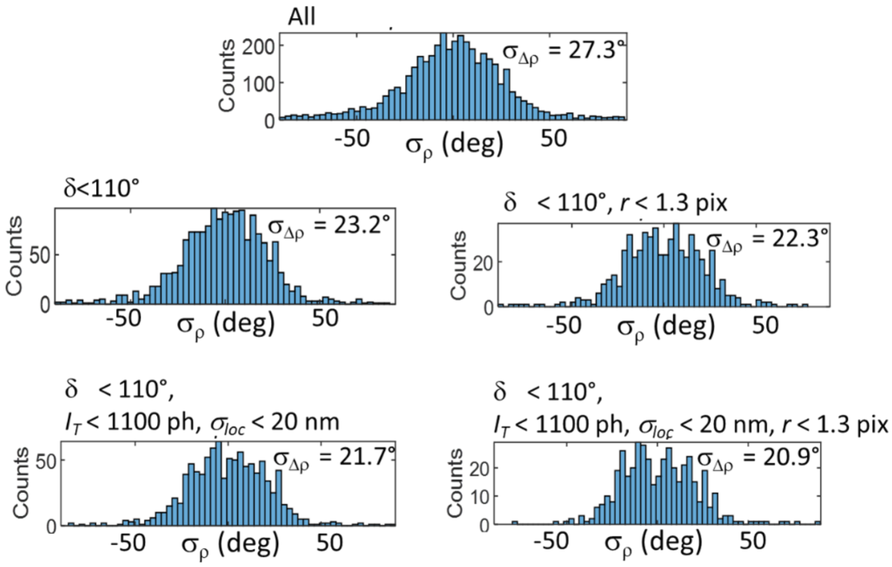

Figure S24. Effect of the detection-parameter filtering on the Δρ statistics for the population δ < 110°. (a) statistics on single molecules in the ROI1 represented in figures 2,3 of the main text. (The detection parameters filtering does not induce a large modification of the Δρ for the population δ < 110° is considered. Source data are provided as a Source Data file (https://doi.org/10.6084/m9.figshare.17167001).

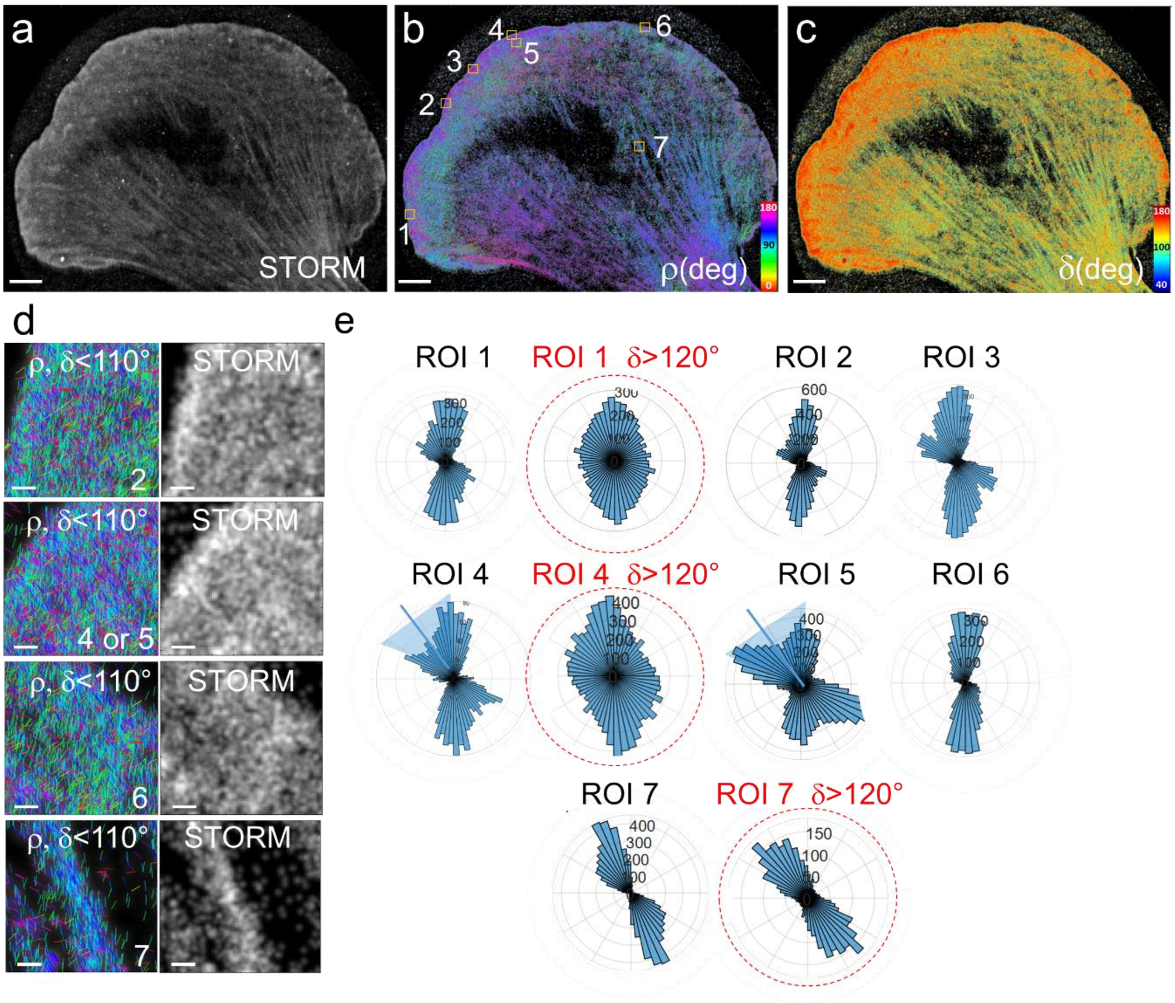

Figure S25. 4polar-STORM imaging of actin filament organization in lamellipodia. (a) Single molecule localization STORM image of a B16 cell labelled with AF488-phalloidin. (b) Corresponding 4polar-STORM ρ stick image with color-coded orientation measurements. (c) 4polar-STORM δ stick image with color-coded wobbling angle measurements. (d) Examples of ρ stick images showing molecules with δ < 110° and corresponding STORM images in selected ROIs (squares in (b)). ROIs 1-6, regions in the lamellipodium; ROI 7, SF. (e) Polar-plot histograms of ρ for the regions shown in (b). The condition δ < 110° is used, except for red-circled histograms for which δ > 120° molecules are selected. The blue line corresponds to the direction of the normal to the membrane at the ROI position, and the blue rectangle is a guideline for an angular aperture of 70°. Scale bars (a-c), 4 μm; (d), 260 nm. Source data are provided as a Source Data file (https://doi.org/10.6084/m9.figshare.17167001).

Supplementary Information References

1. Backer, A. S. & Moerner, W. E. Determining the rotational mobility of a single molecule from a single image: a numerical study. *Opt. Express* **23**, 4255 (2015).
2. Forkey, J. N., Quinlan, M. E. & Goldman, Y. E. Protein structural dynamics by single-molecule fluorescence polarization. *Prog. Biophys. Mol. Biol.* **74**, 1–35 (2000).
3. Axelrod, D. Chapter 7 Total Internal Reflection Fluorescence Microscopy. *Methods in Cell Biology* vol. 89 169–221 (2008).
4. Yan, T., Richardson, C. J., Zhang, M. & Gahlmann, A. Computational correction of spatially variant optical aberrations in 3D single-molecule localization microscopy. *Opt. Express* **27**, 12582 (2019).
5. C. R. Cantor and P. R. Schimmel. *Biophysical Chemistry. Part II: Techniques for the Study of Biological Structure and Function*.
6. Brasselet, S. Polarization-resolved nonlinear microscopy: application to structural molecular and biological imaging. *Adv. Opt. Photonics* **3**, 205 (2011).
7. Lew, M. D., Backlund, M. P. & Moerner, W. E. Rotational Mobility of Single Molecules Affects Localization Accuracy in Super-Resolution Fluorescence Microscopy. *Nano Lett.* **13**, 3967–3972 (2013).
8. Valades Cruz, C. A. *et al.* Quantitative nanoscale imaging of orientational order in biological filaments by polarized superresolution microscopy. *Proc. Natl. Acad. Sci. U. S. A.* **113**, E820–E828 (2016).
9. Petrov, P. N., Shechtman, Y. & Moerner, W. E. Measurement-based estimation of global pupil functions in 3D localization microscopy. *Opt. Express* **25**, 7945–7959 (2017).
10. Sergé, A., Bertaux, N., Rigneault, H. & Marguet, D. Dynamic multiple-target tracing to probe spatiotemporal cartography of cell membranes. *Nat. Methods* (2008) doi:10.1038/nmeth.1233.
11. Pengo, T., Holden, S. J. & Manley, S. PALMsiever: A tool to turn raw data into results for single-molecule localization microscopy. *Bioinformatics* **31**, 797–798 (2015).
12. Botev, Z. I., Grotowski, J. F. & Kroese, D. P. Kernel density estimation via diffusion. *Ann. Stat.* **38**, 2916–2957 (2010).
