# Supplementary material for: 4polar-STORM polarized super-resolution imaging of actin filament organization in cells
Source: Nat Commun. 2022 Jan 13;13:301. doi: 10.1038/s41467-022-27966-w (PMC8758668; doi:10.1038/s41467-022-27966-w)
Supplement: Supplementary file 2 — Reporting Summary [file 41467_2022_27966_MOESM2_ESM.pdf]

nature portfolio

Corresponding author(s): Sophie Brasselet, Manos Mavrakis

Last updated by author(s): Dec 12, 2021

Reporting Summary

Nature Portfolio wishes to improve the reproducibility of the work that we publish. This form provides structure for consistency and transparency in reporting. For further information on Nature Portfolio policies, see our Editorial Policies and the Editorial Policy Checklist.

Statistics

For all statistical analyses, confirm that the following items are present in the figure legend, table legend, main text, or Methods section.

n/a	Confirmed	
[ ]	[x]	The exact sample size (n) for each experimental group/condition, given as a discrete number and unit of measurement
[ ]	[x]	A statement on whether measurements were taken from distinct samples or whether the same sample was measured repeatedly
[ ]	[x]	The statistical test(s) used AND whether they are one- or two-sided<br>*Only common tests should be described solely by name; describe more complex techniques in the Methods section.*
[x]	[ ]	A description of all covariates tested
[x]	[ ]	A description of any assumptions or corrections, such as tests of normality and adjustment for multiple comparisons
[ ]	[x]	A full description of the statistical parameters including central tendency (e.g. means) or other basic estimates (e.g. regression coefficient) AND variation (e.g. standard deviation) or associated estimates of uncertainty (e.g. confidence intervals)
[ ]	[x]	For null hypothesis testing, the test statistic (e.g. F, t, r) with confidence intervals, effect sizes, degrees of freedom and P value noted<br>*Give P values as exact values whenever suitable.*
[x]	[ ]	For Bayesian analysis, information on the choice of priors and Markov chain Monte Carlo settings
[x]	[ ]	For hierarchical and complex designs, identification of the appropriate level for tests and full reporting of outcomes
[x]	[ ]	Estimates of effect sizes (e.g. Cohen's d, Pearson's r), indicating how they were calculated

Our web collection on statistics for biologists contains articles on many of the points above.

Software and code

Policy information about availability of computer code

Data collection	Images were recorded using the software associated to the used CCD camera : Andor Solis 64-bit (v 4.32).
Data analysis	The algorithms are written in Matlab (v2019b). The detection algorithm is published in Valades et al. https://doi.org/10.1073/pnas.1516811113. The post processing algorithm is available with test data and explanations at : https://gitlab.fresnel.fr/mosaic/4polarSTORM. Data image processing use DIPimage (v 2.9). Statistical analyzes use the Matlab tttest function (v2019b).

For manuscripts utilizing custom algorithms or software that are central to the research but not yet described in published literature, software must be made available to editors and reviewers. We strongly encourage code deposition in a community repository (e.g. GitHub). See the Nature Portfolio guidelines for submitting code & software for further information.

Data

Policy information about availability of data

All manuscripts must include a data availability statement. This statement should provide the following information, where applicable:

- Accession codes, unique identifiers, or web links for publicly available datasets
- A description of any restrictions on data availability
- For clinical datasets or third party data, please ensure that the statement adheres to our policy

The 4polar-STORM raw image stacks are available on request from the corresponding author, for their large size reason. A subset of data (12Go) as well as their processed localization data in polarized channels are available at https://amubox.univ-amu.fr/s/d6Mcoyc4SbtRr3Z. Processed data of example ROIs generated in this study are available for download at https://gitlab.fresnel.fr/mosaic/4polarSTORM (TestData folder) with explanations of use in the README.md file. All processed orientation/detection-parameters from single molecule data generated in this study are provided in the Source Data file available at https://amubox.univ-amu.fr/s/d6Mcoyc4SbtRr3Z.

Field-specific reporting

Please select the one below that is the best fit for your research. If you are not sure, read the appropriate sections before making your selection.

- [x] Life sciences
- [ ] Behavioural & social sciences
- [ ] Ecological, evolutionary & environmental sciences

For a reference copy of the document with all sections, see nature.com/documents/nr-reporting-summary-flat.pdf

Life sciences study design

All studies must disclose on these points even when the disclosure is negative.

Sample size	For the study of different stress fiber types, a similar number of regions per type was collected for comparative studies. The number of regions chosen was deduced from the number of independent cells/filaments measured : a test showed that adding extra ROIs did not modify the statistics of the comparative results.
Data exclusions	No data were excluded from the analyses
Replication	All data were found to be reproducible over the different cells / single filaments measured. The statement on how many independent samples and cells/filaments per samples have been added in the figure legends, with statement that similar results were obtained among different cells/filaments.
Randomization	There was no specific allocation to experimental groups in the study
Blinding	There was no need for data allocation/blinding in the study : indeed statistical studies in this work concern comparison of different stress fiber categories that needed first to be identified by the user on the cell images.

Reporting for specific materials, systems and methods

We require information from authors about some types of materials, experimental systems and methods used in many studies. Here, indicate whether each material, system or method listed is relevant to your study. If you are not sure if a list item applies to your research, read the appropriate section before selecting a response.

Materials & experimental systems

n/a	Involved in the study	
x		Antibodies
	x	Eukaryotic cell lines
x		Palaeontology and archaeology
x		Animals and other organisms
x		Human research participants
x		Clinical data
x		Dual use research of concern

Methods

n/a	Involved in the study	
x		ChIP-seq
x		Flow cytometry
x		MRI-based neuroimaging

Eukaryotic cell lines

Policy information about cell lines

Cell line source(s)	U2OS cells from Flavio Maina (IBDM, France); U2OS CA-MLCK cells from Sanjay Kumar (UC Berkeley, USA); B16-F1 cells from Klemens Rottner (Technische Universität Braunschweig, Germany). U2OS cells were originally from ATCC (HBT-96) and B16-F1 from ATCC (CRL-6323)
Authentication	None of the cell lines used were authenticated.
Mycoplasma contamination	The cell lines were not tested for mycoplasma contamination.
Commonly misidentified lines (See ICLAC register)	No commonly misidentified cell lines were used in the study
